# Multiomics analysis reveals signatures of selection and loci associated with complex traits in pigs

**DOI:** 10.1002/imt2.250

**Published:** 2024-12-15

**Authors:** Lei Liu, Guoqiang Yi, Yilong Yao, Yuwen Liu, Jiang Li, Yalan Yang, Mei Liu, Lingzhao Fang, Delin Mo, Longchao Zhang, Yonggang Liu, Yongchao Niu, Liyuan Wang, Xiaolu Qu, Zhangyuan Pan, Lei Wang, Muya Chen, Xinhao Fan, Yun Chen, Yongsheng Zhang, Xingzheng Li, Zhen Wang, Yijie Tang, Hetian Huang, Pengxiang Yuan, Yuying Liao, Xinjian Li, Zongjun Yin, Di Liu, Dongjie Zhang, Quanyong Zhou, Wangjun Wu, Jicai Jiang, Yahui Gao, George E. Liu, Lixian Wang, Yaosheng Chen, Kui Li, Martien A. M. Groenen, Zhonglin Tang

**Affiliations:** ^1^ Shenzhen Branch, Guangdong Laboratory of Lingnan Modern Agriculture, Key Laboratory of Livestock and Poultry Multi‐omics of MARA, Agricultural Genomics Institute at Shenzhen Chinese Academy of Agricultural Sciences Shenzhen China; ^2^ Kunpeng Institute of Modern Agriculture at Foshan, Agricultural Genomics Institute Chinese Academy of Agricultural Sciences Foshan China; ^3^ Yazhouwan National Laboratory Sanya China; ^4^ Guangxi Engineering Centre for Resource Development of Bama Xiang Pig Bama China; ^5^ Biozeron Shenzhen, Inc. Shenzhen China; ^6^ College of Animal Science and Technology Hunan Agricultural University Changsha China; ^7^ Center for Quantitative Genetics and Genomics Aarhus University Aarhus Denmark; ^8^ State Key Laboratory of Biocontrol, School of Life Sciences Sun Yat‐sen University Guangzhou China; ^9^ Institute of Animal Sciences Chinese Academy of Agricultural Sciences Beijing China; ^10^ Faculty of Animal Science and Technology Yunnan Agricultural University Kunming China; ^11^ College of Animal Sciences and Technology Henan Agricultural University Zhengzhou China; ^12^ Guangxi Veterinary Research Institute Nanning China; ^13^ College of Animal Science and Technology Anhui Agricultural University Hefei China; ^14^ Institute of Animal Husbandry Heilongjiang Academy of Agricultural Sciences Haerbin China; ^15^ Institute of Animal Husbandry and Veterinary Medicine Jiangxi Academy of Agricultural Sciences Nanchang China; ^16^ Department of Animal Genetics, Breeding and Reproduction College of Animal Science and Technology Nanjing Agricultural University Nanjing China; ^17^ Department of Animal Science North Carolina State University Raleigh North Carolina USA; ^18^ Animal Genomics and Improvement Laboratory, Henry A. Wallace Beltsville Agricultural Research Center, Agricultural Research Service, USDA Beltsville Maryland USA; ^19^ Animal Breeding and Genomics Group Wageningen University & Research Wageningen The Netherlands

**Keywords:** epigenetics, genomic variation, pan‐genome, phenotype differentiation, pig, skeletal muscle development

## Abstract

The genetic basis of complex traits and phenotypic differentiation remains unclear in pigs. Using nine genomes—seven of which were newly generated, high‐quality de novo assembled genomes—and 1081 resequencing genomes, we built a pan‐genome and identified 134.24 Mb nonredundant nonreference sequences, 1099 novel protein‐coding genes, 187,927 structural variations (SVs) and 30,143,962 single‐nucleotide polymorphisms (SNPs). Analysis of selective domestication revealed *BRCA1* associated with enhanced adipocyte growth and fat deposition, and *ABCA3* linked to an alleviated immune response and reduced lung injury. Integrating 162 transcriptomes and 162 methylomes of skeletal muscle across 27 developmental stages revealed the regulatory mechanism of phenotypic differentiation between Eastern and Western breeds. Artificial selection reshaped local DNA methylation status and imparted regulatory effects on the progression patterns of heterochronic genes such as *GHSR* and *BDH1*, particularly during embryonic development. Altogether, our work provides valuable resources for understanding molecular mechanisms behind phenotypic variations and enhancing the genetic improvement programs in pigs.

## Highlights

- Combining nine high‐quality genomes and 1081 whole‐genome resequencing data, a relatively complete pan‐genome and genetic variation data set of pigs was constructed, laying the foundation for analyzing the complex traits of pigs.
- Abundant signals and genes of selective sweeps related to pig fat deposition, muscle growth, and immune traits were discovered, providing key genetic markers for future breeding programs.
- Integration of transcriptome and methylome data across 27 skeletal muscle development stages revealed the epigenetic and heterochronic gene regulatory mechanisms of skeletal muscle growth differences between Eastern and Western pig breeds.
- Artificial selection has a significant impact on DNA methylation and heterochronic gene regulation, which is closely related to the differences in skeletal muscle growth and meat quality between Eastern and Western pig breeds.

## Graphical abstract

By combining nine high‐quality pig genomes and 1081 resequencing data sets, researchers constructed a comprehensive pan‐genome and genetic variation data set for pigs, laying the groundwork for analyzing complex traits. This data set identified selective sweep signals and genes related to fat deposition, muscle growth, and immune function, providing genetic markers for breeding. Integration of transcriptome and methylome data from 27 muscle development stages revealed epigenetic mechanisms behind muscle growth differences between Eastern and Western pig breeds. Artificial selection influenced DNA methylation and gene regulation, affecting muscle growth and meat quality. Key genes, such as *GHSR* and *BDH1*, regulate skeletal muscle development and meat production, while *ABCA3* impacts immune response and lung injury, and *BRCA1* is linked to adipocyte growth and fat deposition.

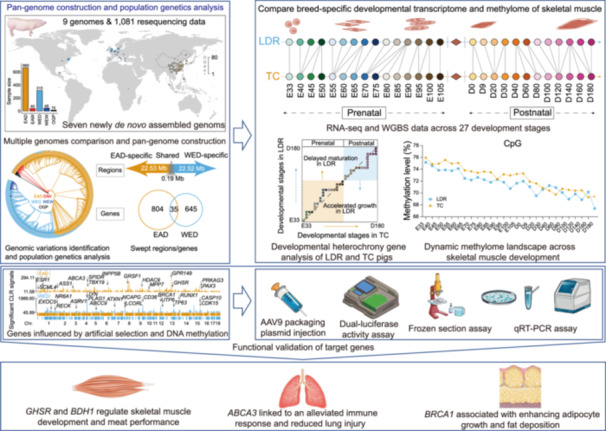

## INTRODUCTION

Pigs (*Sus scrofa*) were independently domesticated as a major global farm animal in Anatolia and South China about 10,000 years ago [1]. Subsequent natural and artificial selection contributed to the formation of approximately 600 breeds with phenotypic diverse and environmental adaptations [2, 3, 4, 5]. Particularly, some Western breeds have been intensively selected over the past 100 years and show remarkable differences in economic traits such as body weight, growth rate, and intramuscular fat percentage compared with Eastern breeds [2, 3]. Besides providing an abundance of agricultural products, pigs can also serve as a valuable biomedical model for human diseases and are promising potential organ donors for xenotransplantation [6, 7, 8]. Therefore, exploring the genetic changes and selection regimes behind pig domestication and breeding will have great value at biological, medical, and economic levels.

In the past decade, several studies have attempted to mine the significant consequences of pig domestication and suggested numerous quantitative trait loci (QTLs) and genes with potential functional implications in phenotypic diversification, such as *EPAS1*, *ESR1*, *KIT*, *LCORL*, *MC1R*, *MUC13*, *NR6A1*, *PLAG1*, *RYR1*, and *VRTN* [1, 4, 9, 10, 11, 12, 13, 14, 15]. Identifying these pivotal genes accelerates the pig breeding process; however, numerous genetic determinants remain undiscovered. Hence, comprehensive genetic survey with larger sample sizes and a broader range of pig breeds is urgently required. Furthermore, an increasing body of evidence has revealed much more prominent roles of regulatory elements in the domestication process due to a lack of detrimental pleiotropic effects compared with coding mutations [16, 17, 18, 19]. Variants in regulatory elements can affect the spatiotemporal expression patterns of key developmental genes, and even subtle transcriptional changes have the potential to impact the developmental timing of specific organs, potentially resulting in phenotypic alterations in the long run [13, 20]. Nevertheless, how artificial selection sculpts phenotypic diversity by reshaping the genome, transcriptome, and epigenome remains largely unknown.

To enhance understanding of (epi)genetic substrates underpinning economic traits and especially their contributions to morphogenesis in pigs, we newly generated seven high‐quality de novo assembled genomes and conducted a joint analysis of whole‐genome resequencing data from 1081 individuals, and time‐course transcriptome and methylome data of skeletal muscle across 27 developmental stages in two representative Eurasian pig breeds. In this study, we established the comprehensive pan‐genome (the sum of the genome collections of all individuals within a certain biological population) and genetic variation repertoire. We revealed abundant selective sweep signatures underlying economically important traits and further highlighted promising heterochronic genes and DNA methylation variations under artificial selection related to myogenesis and meat production traits. Overall, these results provide novel insights into the (epi)genetic and phenotypic divergence between Eastern and Western pigs and nominate some promising determinants for future pig breeding programs and human biomedical research.

## RESULTS

### A pan‐genome sequence and genetic variation data set in pigs

**Figure 1:**
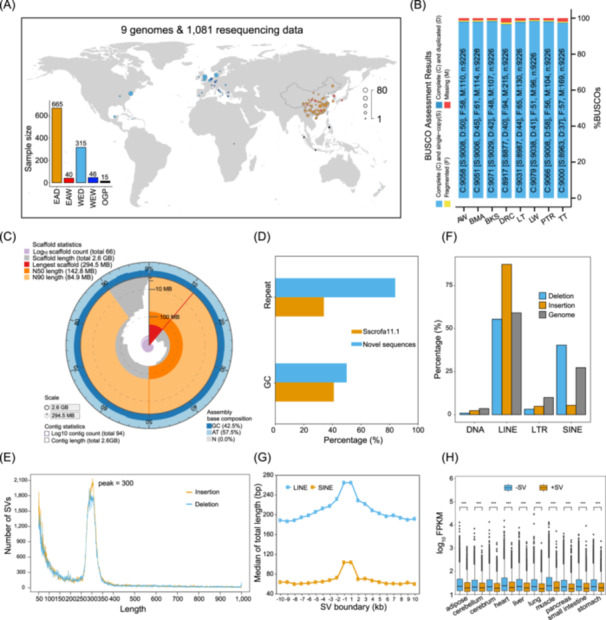
Comprehensive landscape of pig pan‐genome. (A) Geographic locations of all breeds and species for the five categories. The histogram shows the sample size of each category. (B) BUSCO assessment results for genome assemblies of eight pig breeds. The Duroc genome (DRC) is the reference assembly that was downloaded from the Ensembl database. AW: Asian wild boar; BMA: Bama Xiang, BKS: Berkshire; LT: Lantang; LW: Large White; PTR: Piétrain; TT: Tibetan. (C) Snail plots describing the assembly statistics of the Piétrain breed. Other six genomes were displayed in Figure S2. (D) The percentages of repeat sequence and GC content in the reference genome and novel nonredundant nonreference sequences. (E) Size distribution of putative structural variants. (F) Enrichment of structural variants in repeat elements. (G) Distribution of repeat elements around structural variants boundary. (H) The expression difference between genes with and without structural variants in multiple tissues. The statistical method used was the Wilcoxon test. ***p < 0.001. EAD, Eastern domestic pigs; EAW, Eastern wild boars; FPKM, fragments per kilobase of transcript per million mapped reads; LINE, long interspersed nuclear element; LTR, long terminal repeat; OGP, outgroup (other Suids); SINE, short interspersed nuclear element; SV, structural variant; WED, Western domestic pigs; WEW, Western wild boars.

To capture a more comprehensive set of genomic sequences present in pigs than the reference genome Sscrofa11.1, we first performed PacBio High‐Fidelity (HiFi) sequencing on one Asian wild boar (AW), three breeds from China (BMA: Bama Xiang, TT: Tibetan, and LT: Lantang), and three breeds from Europe (LW: Large White, BKS: Berkshire, and PTR: Piétrain) (Figure 1A and Figure S1). An average coverage of 52.8× highly accurate long reads with per‐base accuracy >99.9% per sample was obtained (Table S1). Subsequently, we generated seven chromosome‐level assemblies with an average length of 2.68 Gb by high‐throughput chromosome conformation capture (Hi‐C) based assembly (Tables S2 and S3), which is slightly bigger than Sscrofa11.1 (2.68 vs. 2.50 Gb). Transposable elements (TEs) make up 38.8% of each genome ranging from 35.8% to 40.5% (Table S4). Notably, 82.33% of the telomere sequences and all the centromeric repeat regions were predicted. The average GC content of the centromeric repeat regions was higher than that in the Duroc pig genome (49.5% vs. 41.4%), and the average repeat content exceeded that of the entire genome (65.7% vs. 38.8%) (Tables S5 and S6). The seven genomes have greater completeness and consistency based on the resulting assembly statistics and pair‐wise colinear patterns compared with the reference genome from Duroc pigs. These genomes will serve as a rich resource for future research in pig genetics and genomics (Figure 1B,C, Figures S2 and S3, and Tables S7–S9). We further integrated two chromosome‐level genomes, the Sscrofa11.1 reference, and a previous Luchuan genome reported by our group [21] to build a high‐resolution pan‐genome (Table S8). A total of 134.24 Mb nonredundant nonreference sequences with an N50 of 287,722 bp was obtained (Table S10). Of these, 100.52 Mb came from Chinese native breeds (BMA, TT, LT, and Luchuan), 16.43 Mb came from AW, and 17.29 Mb came from European breed pigs (LW, BKS: Berkshire, PTR). We observed a higher GC content in the novel sequences than in the reference genome (49.5% vs. 41.4%) (Figure 1D). Approximately 83.72% of the nonredundant nonreference sequences comprised repetitive elements, which is higher than the reference genome. In total, 1099 novel protein‐coding genes were detected with a coding sequence (CDS) N50 size of 774 based on the newly constructed pan‐genome. Of these, 94.54% (1039 genes) were supported by transcript evidence, and 77.53% (852 genes) were successfully annotated for ≥1 function term (Table S11). The Sscrofa11.1 assembly together with the putative nonredundant nonreference sequences were used as the final reference genomes for subsequent analyses.

To enable a more complete and equitable understanding of genomic diversity, we anchored these assembled genome sequences onto the reference genome using the AnchorWave program [22]. A total of 187,927 structural variations (SVs) with a peak at a size of 300 bp were found (Figure 1E), which was consistent with previous results [23, 24]. We observed that only 16.6% of base pairs overlapped between our SV discovery results and the SV callsets based on Illumina data [25]. Moreover, 96.8% of the SVs identified in this study were novel, highlighting the sensitivity of our SV detection compared with short‐read sequencing methods. Notably, we found that long interspersed nuclear elements (LINEs) and short interspersed nuclear elements (SINEs) are two major contributors driving SVs, and mainly enriched within SVs and around SVs boundaries (Figure 1F,G). In addition, 7,667,617 small insertions and deletions (INDELs, referring ≤50 bp in this study) were identified (Figure S4). In the sequence fragment distribution plot, the even number was higher than the adjacent odd number (Figure S4). By leveraging multitissue RNA‐seq data, we found that SVs generally exerted negative effects on the expression levels of flanking genes (Figure 1H).

To understand the difference in genetic variation repertoire and phenotype between obese‐ and lean‐type pigs, we gathered whole‐genome sequencing data from 1081 individuals with 313 newly generated (Tables S12 and S13), mainly distributed in Europe and Asia (Figure 1A). We grouped all these individuals into five categories: an Eastern domestic group (EAD) including 665 pigs from 53 Chinese breeds (~60% of all Chinese pig breeds) and one Korean indigenous breed, a Western domestic group (WED) containing 315 pigs from 25 typical Western breeds, an Eastern wild group (EAW) comprising 40 wild boars from Asia, a Western wild group (WEW) including 46 European wild boars, and 15 individuals from five different wild pig species (*Suidae*)—*Sus barbatus*, *Sus cebifrons*, *Sus celebensis*, *Sus verrucosus*, and *Phacochoerus africanus*—that served as the outgroup (OGP). The entire sequencing data of 45.3 Tb (Table S14) was used for short variant discovery, a final set of 30,143,962 single‐nucleotide polymorphisms (SNPs) and 5,496,594 INDELs after removing low‐quality variants was identified, comparing with the single‐nucleotide polymorphism database (dbSNP) of National Center for Biotechnology Information (NCBI) and the variation archive of European Bioinformatics Institute (EBI) in pigs, the results showed that 5,277,943 novel SNPs were identified. It also showed clear enrichment of variants at the end of the chromosomes and a lower degree of variation on the X chromosome (Figure S5A,B). These variants showed a clear depletion within exons but high levels of enrichment within introns and intergenic regions (Figure S5C). Most of the SNPs and INDELs were located in intergenic (59.52% and 59.08%) and intronic (37.55% and 38.47%) regions, whereas only 0.56% of SNPs and 0.10% of INDELs were present in CDS (Table S15).

### Population structure and potential selection signatures

**Figure 2:**
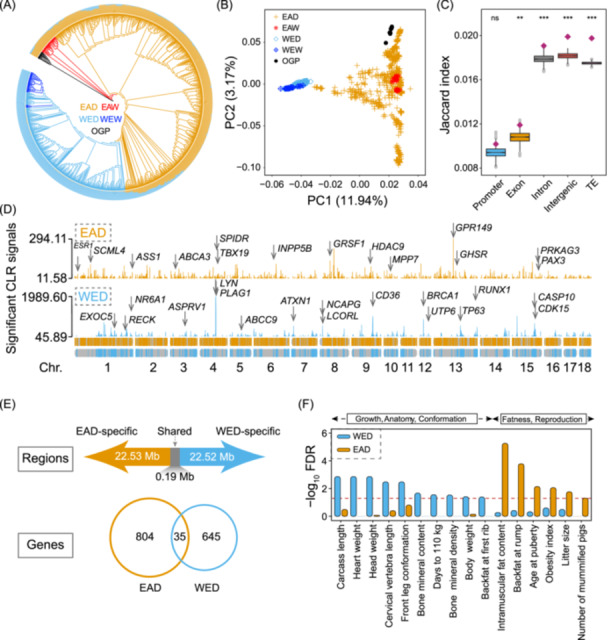
Population structure and selective sweeps in Eastern and Western pig populations. (A) Population structure of all 1081 individuals. The neighbor‐joining phylogenetic tree includes 665 Eastern domestic pigs (EAD), 315 Western domestic pigs (WED), 40 Eastern wild boars (EAW), 40 Western wild boars (WEW), and 15 individuals from 5 other Sus species and *Phacochoerus africanus* (OGP). The circular barplot indicates the individual ancestry coefficient for each sample in which we set the *K* value as 2. (B) Principal component analysis based on all putative autosomal SNPs. (C) Overlap analysis between putative selective sweeps and promoter, exon, intron, intergenic, and transposable elements by Jaccard index score. The statistical method used was the permutation test. **p < 0.01; ***p < 0.001; ns, not significant. (D) Manhattan plots for all significant selective sweeps in Eastern and Western pig groups. Promising candidate genes are marked in the graph. This result implied that Western domestic pigs were subject to stronger selection pressures. Please note that the *y*‐axis range of WED is different from that of EAD and the WED is larger. (E) The shared and unique genomic intervals and genes under selection between Eastern and Western pigs. (F) Functional enrichment analysis of Eastern and Western swept genes against the pig QTL database. TE, transposable element.

Population structure analysis according to the neighbor‐joining method displayed a clear subdivision into five clades, despite the presence of ancestry admixture between certain EAD and WED breeds (Figure 2A). This inferred relationship was broadly consistent with principal component analysis (PCA) patterns, exhibiting an ancestry admixture between Eastern and Western pigs (Figure 2B). By removing admixed individuals, we finally retained 953 individuals for subsequent analysis. By selecting the top 1% of putative selective sweeps based on computed composite likelihood ratios (CLRs), we identified around 227 Mb with significant selection footprints in EAD and WED groups. The permutation test revealed that selection signatures were more enriched in introns, intergenic regions, and TEs compared with promoters and exons (Figure 2C). Moreover, the selection signatures were more enriched in LINE (Jaccard index = 0.020, *p* < 0.001) and SINE (Jaccard index = 0.017, *p* < 0.001) compared with long terminal repeat (LTR) (Jaccard index = 0.014, *p* < 0.001), indicating that LINE and SINE contributed more to selective domestication than LTR.

Selective sweeps in WED showed higher signals and more concentrated locations (Figure 2D) than those in the EAD group. We found 925 and 700 candidate genes in EAD‐ and WED‐specific selective sweep regions (Tables S16 and S17), respectively. We also found 245 regions overlapping between EAD and EAW, with a size of 2,570,028 bp. Additionally, 36 regions overlapped between WED and WEW, with a size of 415,361 bp. Furthermore, we repeated the selective sweep analyses based on two additional approaches—fixation index (FST) and nucleotide diversity (Pi)—to enable the robustness of our results. The pairwise comparisons of different groups are listed in (Tables S18–20). Notably, the number of selection footprints and genes shared between Eastern and Western pigs was limited (Figure 2E), supported by the overlapping results from Pi methods (Tables S21 and S22). Among all 1625 putative genes under selection, we found several candidates reported by previous studies (Figure 2D), including *CASP10*, *ESR1*, *LCORL*, *NCAPG*, *NR6A1*, and *PLAG1* [4, 10, 11, 26].

Besides, we provided a list of novel observations with respect to swept genes that were worth further exploration (Figure 2D and Figure S6A), such as *MPP7* for reproduction traits and *PRKAG3* for meat quality [26, 27, 28]. Many transcription factors (TFs) such as *PAX3*, *RUNX1*, *TP63*, and *TBX19* involved in many vital biological processes were found to be under selection. Several swept intervals were located far from genes of particular functions, such as *E2F2* and *MYOG*, and might act as intergenic or distal functional elements. Gene ontology (GO) enrichment analysis revealed that the swept gene set from EAD was significantly enriched in developmental growth and response to growth factor terms. In contrast, overrepresented terms in the WED group mainly referred to the Wnt signaling pathway, cardiovascular system development, and organelle localization (Figure S6B). We further probed the phenotypic consequences of swept genes by integration with the animal QTL database of pigs [29] and provided more support for notably different selection directions between EAD and WED breeds. The hypergeometric test showed that traits driven by EAD swept genes were involved in fatness and reproduction classes, and genes under selection in the WED group primarily concerned growth, anatomy, and conformation categories (Figure 2F and Tables S23 and S24).

### Functional implication of coding variants linked to selective sweeps

**Figure 3:**
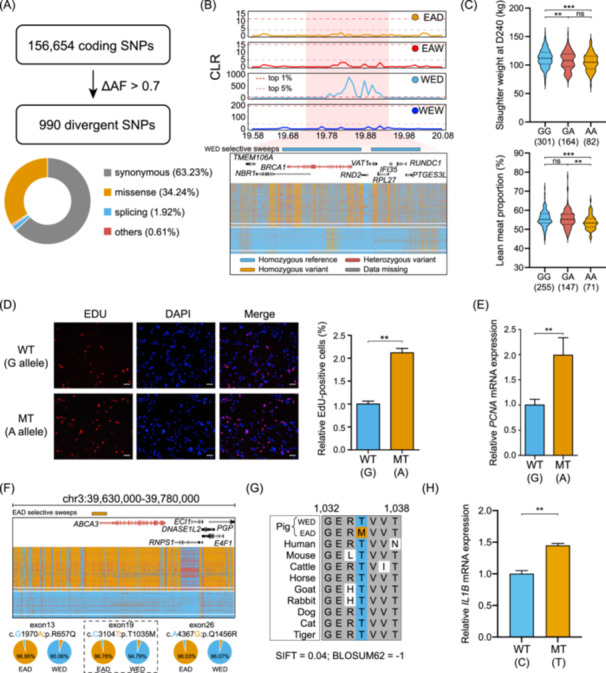
Functional implications of coding variants driven by selective sweeps. (A) Annotation of coding variants with delta allele frequency (∆AF) between EAD and WED groups larger than 0.7. (B) The composite likelihood ratio (CLR) values and genotype patterns in the *BRCA1* region among EAD, EAW, WED, and WEW groups. Dashed purple and red lines represent thresholds of the top 5% and 1% selective sweeps, respectively. (C) Significant differences in slaughter weight at Day 240 and lean meat proportion contributed by a coding SNP of the *BRCA1* gene. The statistical method used was one‐way analysis of variance (ANOVA). **p < 0.01; ***p < 0.001; ns, not significant. (D) Comparison of EdU proliferation assays for porcine adipocyte between the wild‐type and mutant alleles. The Edu‐positive cell of the mutation‐type A allele is significantly higher than that of the wild‐type G allele, indicating that the mutation‐type A allele in *BRCA1* has a higher adipocyte proliferation capacity than the wild‐type G allele. The values were expressed as the mean ± standard deviation (SD). The statistical method used was the Student's *t* test. **p < 0.01. (E) Increased proliferation capacity of mutant allele according to higher expression of *PCNA* marker by quantitative reverse transcription polymerase chain reaction (qRT‐PCR). *PCNA* is an important proliferation marker gene. *PCNA* gene expression in mutation‐type A allele cells was significantly higher than that in wild‐type G allele cells, indicating that the G to A mutation in *BRCA1* increased the proliferation ability of adipocytes. The values were expressed as the mean ± SD. The statistical method used was the Student's *t* test. **p < 0.01. (F) The genotype landscape in the *ABCA3* region among EAD, EAW, WED, and WEW groups. Three nearly fixed coding variants were found in this region. (G) Multiple sequence alignment of amino acids across 10 representative mammalian species. (H) Alleviated immune injury of wild‐type allele according to lower expression of *IL1B* marker by qRT‐PCR. *IL1B* is a commonly used marker gene to evaluate immune damage. There is a positive correlation between immune damage and *IL1B* expression levels. The values were expressed as the mean ± SD. The statistical method used was the Student's *t* test. **p < 0.01. SNP, single‐nucleotide polymorphism.

Given that mutations within CDS might affect protein structure and function, we first explored the functional implications of these genomic variants. In total, we detected 156,654 autosomal coding SNPs at a genome‐wide scale. By removing SNPs that are outside the swept genes or have a delta allele frequency (∆AF) ≤0.7 between EAD and WED groups, we finally kept 990 nearly fixed coding variants (Figure 3A). The Ensembl Variant Effect Predictor (VEP) suite returned 626 SNPs (63.23%) to be synonymous, 339 (34.24%) as missense variants, 19 as splicing mutations, and six as stop‐gain and start‐loss mutations (Table S25). Previous studies proposed that a missense substitution (chr1_265,347,265_A/G, c.T575C, or p.Pro192Leu) in exon 5 of the *NR6A1* gene was the causative site affecting the number of vertebrae [10, 30, 31]. Consistently, we provide compelling evidence for this finding and illustrate the conservation and function of the amino acid substitution (Figure S7).

Besides the *NR6A1* gene, we also highlighted several promising candidates related to traits of economic importance. We found two broad locations with a strikingly high signal of selective sweeps only in the WED group (Figure 3B), which harbored eight functional genes including *BRCA1*, *NBR1*, and *RPL27*. Notably, further inspection of this region uncovered a coding mutation of c.G965A (chr12_19,812,845_G/A) located within exon 10 of the *BRCA1* gene. In this site, the mutant‐type A allele was dominated in the EAD group (AF = 91.67%) but nearly absent in the WED group (AF = 3.27%) (Figure S8A–C). Meanwhile, this variant tends to be mildly conserved and possibly deleterious based on Ensembl SIFT (0.06) and BLOSUM62 (–1) scores as well as multispecies alignment (Figure S8D). To understand the effects of this variant, we compared the phenotypic differences of all three genotypes in 589 F_2_ individuals derived from a cross between 5 Large White (European, WED) boars and 16 Min (Chinese, EAD) sows [32], in which the ratio distribution of mutant type A was 73.33% (11/15, resequencing data of 15 Min sows) and 0% (0/4, resequencing data of four Large White boars) in Min and Large White pigs, respectively. We observed that this SNP has an allele substitution effect of 3.55 kg for slaughter weight at 240 days (SW) and 0.62% for lean meat proportion (LMP). At this locus, the AA genotype presented prominently lower SW and LMP than the GG and GA genotypes (Figure 3C). By in vitro adipocyte proliferation assays, the expression of G322D *BRCA1* displayed enhanced capacity in adipocyte growth and development (Figure 3D,E), corresponding to more significant fat deposition in obese EAD pigs, in contrast to WED breeds with the wild‐type G allele.

Moreover, a swept region overlapping the *ABCA3* gene was of particular interest to us due to its highly distinct genotype distribution between EAD and WED groups and lung‐specific expression of this gene (Figure 3F, Figure S9A,B). We pinpointed three nonsynonymous mutations displaying nearly complete LD among one another. Based on the two values SIFT and BLOSUM62, we prioritized the variant (chr3_39,723,445_C/T) in exon 19 as the top candidate, in consideration of its more deleterious effect on protein structure and function (SIFT = 0.04, BLOSUM62 = –1) (Figure 3G, Figure S9C,D). Almost all individuals in the EAD group carried the alternative allele (T) at this site, whereas WED pigs were nearly fixed for the C allele. The C allele in *ABCA3* was also nearly fixed in WED, suggesting that it predated domestication. Since *ABCA3* mutations can cause surfactant deficiency, alveolar cell injury, and inflammation [33, 34], we hypothesized the *ABCA3* gene to be an intriguing genetic determinant responsible for breed‐specific resistance/susceptibility against the pathogen *Mycoplasma hyopneumoniae* (Mhp) [34, 35]. Experimental data via in vitro Mhp infection showed that the wild‐type C allele was associated with lower expression levels of the *IL1B* gene (Figure 3H), which indicated an alleviated immune response and lung injury for this allele.

### Swept genes regulate developmental heterochrony of skeletal muscle between EAD and WED pigs

**Figure 4:**
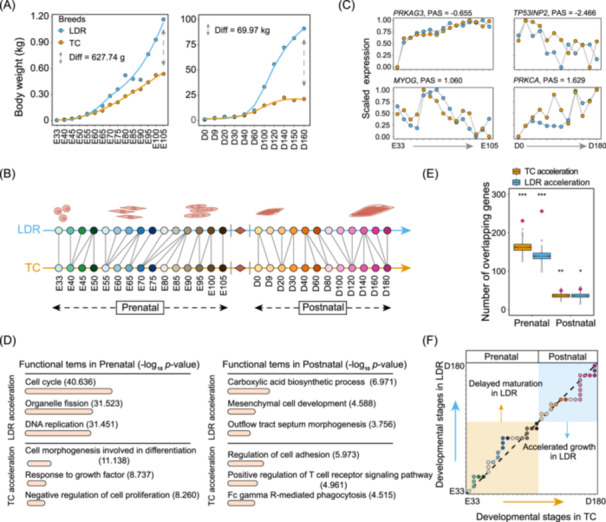
Interbreed heterochrony of porcine skeletal muscle development by swept genes. (A) Differences in body weight of Landrace (LDR) and Tongcheng (TC) pigs at multiple developmental stages. (B) Cross‐breed correspondences of skeletal muscle developmental stages in LDR and TC pigs. (C) Examples of differentially progressing genes (DPGs) in the prenatal and postnatal stages. Based on the definition of TimeMeter, positive progression advanced scores (PAS) indicate a faster expression pattern in TC (TC acceleration genes), while negative means accelerated changes in LDR (LDR acceleration genes). (D) Gene ontology enrichment analysis predicted with LDR and TC acceleration genes in prenatal and postnatal stages. (E) Significant overlaps between DPGs and swept genes. The DPG set was predicted from time‐course RNA‐seq data from LDR and TC pigs. *y*‐axis is the observed (purple diamond) and expected (boxplot) number of overlaps determined by 1000 permutation tests. The statistical method used was the permutation test. *p < 0.05; **p < 0.01; ***p < 0.001. (F) Developmental heterochrony analysis based on only swept genes of LDR and TC pigs. Using LDR as a reference, TimeMeter implements a dynamic time warping algorithm in all RNA‐seq data, to select the best alignment between the time series based on stage transcriptome correlations. This finding manifested that the differences in skeletal muscle development between TC and LDR can be traced back to the very early stages of myogenesis, in which LDR showed more advanced and prolonged timing for myoblast proliferation.

The enrichment analysis of swept genes against the pig QTL database illustrated large differences in growth and carcass characteristics between EAD and WED groups (Figure 2F). To investigate the molecular mechanisms underlying the gradual formation of differences in growth and carcass characteristics between EAD and WED pigs, we compared the developmental trajectories of skeletal muscle across 27 developmental stages from the two representative breeds. Thus, we designated Tongcheng (TC, an Eastern obese‐type breed) and Landrace (LDR, a classical Western lean‐type breed) pigs as research models. Phenotype records revealed that the two breeds showed high divergence in growth rate and muscle mass, and LDR pigs were more than twice as heavy as TC pigs at 160 days of age (Figure 4A). Therefore, we inferred that the growth and development of skeletal muscles of Landrace and Tongcheng pigs should be asynchronous. Subsequently, we newly implemented a time‐series RNA‐seq experiment of 27 time points from skeletal muscle in TC, including 15 prenatal and 12 postnatal stages (Table S26). Comprehensive analyses showed globally consistent transcriptional profiling across skeletal muscle development between TC and LDR (Figures S10 and S11) [36]. We subsequently applied weighted gene co‐expression network analysis (WGCNA) to explore critical functional modules implicated in skeletal muscle development. A total of 18 modules were found (Figure S12A), two of which showed significant overlap with putative swept genes and were primarily concerned with cell division, morphogenesis, and development processes (Figure S12B,C).

Furthermore, we performed a comparative transcriptome analysis based on the dynamic time warping (DTW) algorithm in TimeMeter [37] to dissect time shift patterns during skeletal muscle development between TC and LDR. We provided clues to mild heterochrony throughout skeletal myogenesis, featured by more advanced and prolonged myoblast proliferation in the prenatal stage and faster muscle growth in the postnatal period for LDR, whereas more accelerated myoblast maturation in the prenatal period for TC (Figure 4B, Figure S13A). In total, we detected 4947 and 1184 differentially progressing genes (DPGs) in prenatal and postnatal stages, respectively, including *MYOG*, *TP53INP2*, and *MEF2B* (Figure 4C, Figure S13B and Tables S27 and S28). Based on calculated progression advanced scores (PAS), positive values refer to more advanced expression patterns in developmental progression in TC than those in LDR, which were defined as TC acceleration genes, while negative means accelerated changes in LDR (LDR acceleration genes). Functional enrichment analysis emphasized that LDR acceleration genes in the prenatal stage were significantly overrepresented in cell cycle and DNA replication. In contrast, TC acceleration genes mainly contributed to cell differentiation and maturation (Figure 4D). In the postnatal period, genes with more advanced progressions in LDR were persistently enriched for cell development and biosynthetic process, while TC advanced genes were linked to immune response.

To further elucidate the genetic basis underlying interbreed differences in developmental timing, we evaluated the contributions of swept genes in tuning skeletal myogenesis. Compared with all genes in the pig genome, genes under selection showed higher expression levels at all stages for each breed (Figure S14), implying their essential functions in specific important biological processes. Subsequent permutation tests reported a highly significant overlap between DPGs and swept genes, especially in the prenatal phase (Figure 4E). We conducted the same DTW analysis by focusing on these swept genes and uncovered highly consistent dynamic patterns with results from all expressed genes (Figure 4F).

### Selective sweeps regulated gene expression by reshaping DNA methylation pattern

**Figure 5:**
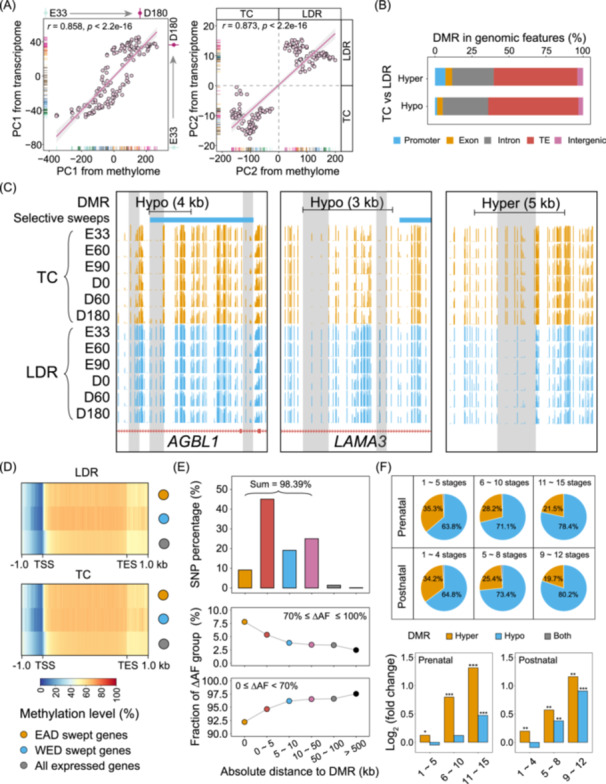
Differences in DNA methylome during pig domestication and breeding. (A) Correlation analysis between methylome and transcriptome for PC1 and PC2. The PC1 and PC2 values were individually calculated from RNA‐seq and whole‐genome bisulfite sequencing (WGBS) data. (B) The stacked graph of the proportion of differentially methylated region (DMR) in five different genomic features including promoter, exon, intron, TE, and intergenic regions. (C) Snapshot of UCSC genome browser showing five significant DMR loci colored by gray and selective sweeps in six representative stages. It suggested that differences in methylation levels in skeletal muscle development of TC and LDR pigs were influenced by domestication and selection. (D) Comparison of DNA methylation levels among EAD selective genes, WED selective genes and all expressed genes. (E) The absolute distance between DMR and the nearest SNPs at a genome‐wide level. (F) Relationships between DNA methylation variation and selective sweeps during prenatal and postnatal skeletal muscle development. In the second panel, we first extracted regions from the genome and counted the number of regions overlapping with DMR that belong to the swept regions. Then we calculated the number of overlapping regions between DMR and the swept regions and conducted significance tests on the results using permutation tests. The fold change represents the ratio between the median obtained from random sampling and the actual count of overlapping regions between DMR and swept regions. The statistical method used was the permutation test. *p < 0.05; **p < 0.01; ***p < 0.001.

DNA methylation is one of the main epigenetic factors regulating gene expression in eukaryotes. To elucidate the contributions of DNA methylation variations in pig domestication and breeding, we performed whole‐genome bisulfite sequencing (WGBS) in skeletal muscle across 27 developmental stages (Table S29), corresponding to the aforementioned samples with RNA‐seq data. We generated a global DNA methylome landscape in TC and LDR and found higher Cytosine–phosphate–Guanine (CpG) methylation levels in TC (Figures S15 and S16A–C). Furthermore, WGBS‐based PCA revealed similar classification patterns with RNA‐seq results but showed a stronger breed effect (Figure S16D). Both the PC1 and PC2 between methylome and transcriptome showed strong curvilinear relationships (*r*_PC1_ = 0.858, *r*_PC2_ = 0.873), suggesting a clear classification of developmental trajectories and breeds (Figure 5A). Functional correlations between these two types of data sets again confirm the results for promoter and gene body regions (Figures S17–S20). By pairwise differential methylation analyses, we obtained 166,265 differentially methylated regions (DMRs) domains (Table S30), approximately 65% of which were hypomethylated in TC compared with LDR. The DMR intersection with five genomic features revealed that both hypermethylated and hypomethylated regions were primarily found in intron and TE regions (Figure 5B). Visualization in the UCSC genome browser exemplified five significant DMR loci with distinct distribution patterns (Figure 5C).

To understand the impacts of selective sweeps on DNA methylations, we performed a correlation analysis between WGBS and RNA‐seq data based on only these swept genes, all expressed genes, and randomly sampled genes, respectively. When comparing the results based on only these swept genes to those based on all expressed genes and randomly sampled genes, we found a reduced regulatory relationship in promoters with decreases in correlation coefficients (0.08 and 0.09 for LDR, and 0.14 for both values in TC), but enhanced patterns in gene bodies with increases in correlation coefficients (0.08 and 0.09 for LDR, and 0.09 and 0.10 for TC) (Figure S21). This finding highlights that sequence contexts under natural and artificial selection could reshape global expression patterns throughout the entire development due to DNA methylation variation. Compared with all expressed genes, promoter regions of putative swept genes generally have lower DNA methylation levels in both TC and LDR breeds, whereas gene bodies displayed overall higher levels of DNA methylation (Figure 5D, Figures S22 and S23). Based on the absolute distances between DMR domains and the nearest SNPs, we found that most DMRs (98.39%) were located within 50 kb of identified SNPs, and around 55% had proximity within 5 kb (Figure 5E). Furthermore, results indicated that these nearly fixed SNPs with ∆AF of more than 0.7 were more prone to being in the vicinity of DMRs, as opposed to SNPs with ∆AF below 0.7. To further clarify the importance of DNA methylation in pig domestication and its relationship with genetic selection, we first separated DMR into three groups regarding the number of stages with significant differences in prenatal and postnatal stages, respectively. We found that the category with persistent DNA methylation variations at almost all stages (11–15 stages in prenatal and 9–12 stages in postnatal) possessed a higher proportion of Hypo‐DMR domains than the other two groups (Figure 5F, upper), supporting our results mentioned earlier. Enrichment analysis, ascertained by permutation tests, showed that DMRs with sustained changes were prone to being covered by selective sweeps in both prenatal and postnatal periods (Figure 5F, lower).

### Genetic basis of candidate genes in skeletal muscle development and meat performance

**Figure 6:**
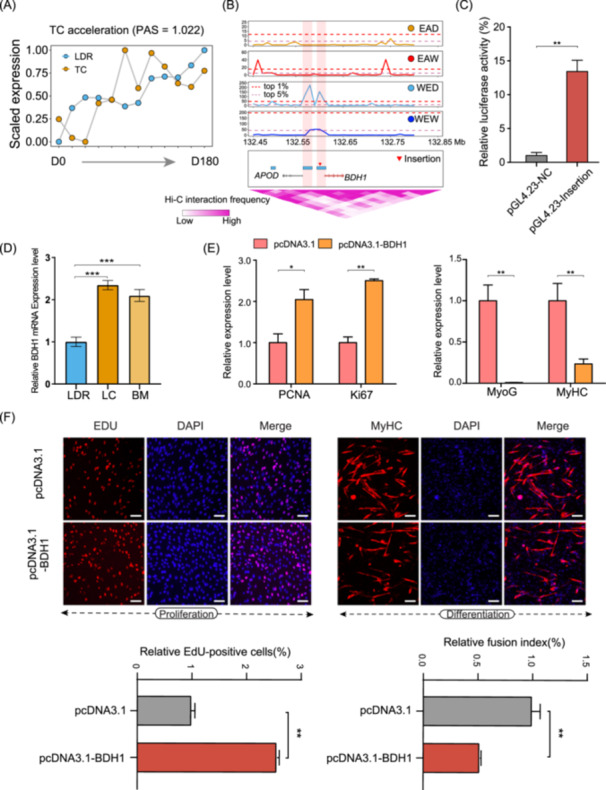
Comprehensive analysis of the *BDH1* gene related to skeletal muscle development. (A) TimeMeter analysis showing the accelerated expression of *BDH1* in the TC postnatal stage. (B) Selective sweeps regions in the four groups. Dashed purple and red lines represent thresholds of the top 5% and 1% CLR values in the *BDH1* region, respectively. (C) Luciferase reporter assays in HEK293T cells to compare enhancer activity between the insertion. The values were expressed as the mean ± SD. The statistical method used was the Student's *t* test. **p < 0.01. (D) Higher gene expression of *BDH1* in Chinese native pig breeds by qRT‐PCR. The expression of *BDH1* in each Chinese native pig breed was compared with that in LDR, respectively. The values were expressed as the mean ± SD. The statistical method used was the Student's *t* test. ***p < 0.001. (E) Cell proliferation assessment for the expression level of *PCNA*, *Ki67*, *MyoG*, and *MyHC* marker upon *BDH1 overexpression* by qRT‐PCR analysis in C2C12 cells. The values were expressed as the mean ± SD. The statistical method used was the Student's *t* test. *p < 0.05; **p < 0.01. (F) Proliferation and differentiation assays of murine C2C12 cells following *BDH1* overexpression. The values were expressed as the mean ± SD. The statistical method used was the Student's *t* test. **p < 0.01.

We first explored the regulatory roles of SVs on candidate genes. An intriguing candidate was the *BDH1* gene, which was mainly involved in the regulation of the ketone metabolic process in skeletal muscle, liver, and heart tissues. The *BDH1* gene showed a more accelerated expression pattern in TC skeletal muscle at the postnatal stage than in LDR (Figure 6A), and we identified a 272 bp insertion located upstream of this gene in Eastern pig breeds. The insertion was covered by a selective sweep with a strong signal (Figure 6B). We speculated that this mutation exerted a positive regulatory role in the *BDH1* gene by a potential chromatin interaction, according to the 3D interaction heatmap (Figure 6B). Luciferase reporter assays indicated increased enhancer activity of this insertion, and Chinese native pig breeds showed higher expression levels of *BDH1* than LDR breeds by quantitative reverse transcription polymerase chain reaction (qRT‐PCR) experiments (Figure 6C,D). Further gene overexpression/suppression analysis revealed enhanced proliferation capacity but weak myoblast differentiation rates (Figure 6E,F and Figure S24).

**Figure 7:**
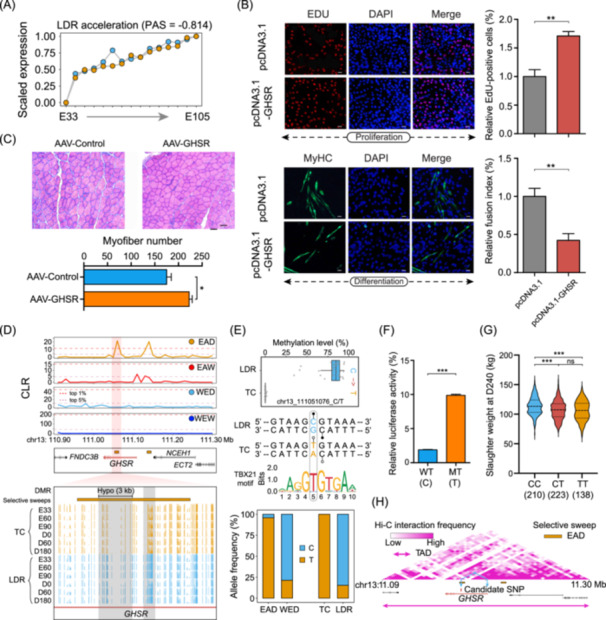
Comprehensive analysis of the *GHSR* gene related to skeletal muscle development and meat performance. (A) TimeMeter analysis showing the accelerated expression of *GHSR* in the LDR prenatal stage. (B) Proliferation and differentiation assays of murine C2C12 cells following *GHSR* overexpression. The values were expressed as the mean ± SD. The statistical method used was the Student's *t* test. **p < 0.01. (C) H&E staining and myofiber number of mice skeletal muscle at 10 days after in vivo injection of AAV‐mediated nontarget control and pcDNA3.1‐GHSR, respectively. Representative images are shown at 20× magnification (scale bars = 100 μm). The statistical method used was the Student's *t* test. *p < 0.05. (D) Selective sweeps reshaping DNA methylation levels across six representative developmental stages in LDR and TC pigs. Dashed purple and red lines represent thresholds of the top 5% and 1% CLR values in the *GHSR* region, respectively. (E) The fixed C to T substitution in *GHSR* resulting in the removal of DNA methylation levels and the creation of a new transcription factor binding site of TBX21. (F) Luciferase reporter assays in HEK293T cells to compare enhancer activity between the two alleles. The values were expressed as the mean ± SD. The statistical method used was the Student's *t* test. ***p < 0.001. (G) Higher slaughter weight at Day 240 of CC genotype. The statistical method used was the one‐way ANOVA. ***p < 0.001. (H) The putative topologically associated domain (TAD) indicating potential interaction between the *GHSR* locus and the candidate CpG‐SNP.

We next focused on the functional consequences of CpG‐SNP mutations from methylated CpG to TpG/CpA in the putative selective sweeps and explored their regulatory functions on heterochronic genes and skeletal muscle development. Furthermore, we found more advanced progressions (PAS = –0.814) of the *GHSR* gene in LDR than in TC during the embryonic period (Figure 7A). We selected this gene mainly based on the five points: (1) this gene is a swept gene; (2) this gene has DMRs between LDR and TC; (3) the mutation site in *GHSR* is close to the DMR; (4) delta allele frequency (ΔAF) of SNP in *GHSR* between EAD and WED groups is higher than 0.7; and (5) this gene is related to growth traits and meat production traits [38, 39]. This result suggested that *GHSR* should be a key contributor to accelerating myoblast proliferation. Multiple experiments supported that *GHSR* could promote myoblast proliferation but inhibit their differentiation and fusion in vivo and in vitro (Figure 7B,C, Figure S25). Meanwhile, a pronounced selection signal was detected in the intron of *GHSR*, which harbored two significant Hypo‐DMRs (Figure 7D). Further analysis in this region revealed a CpG‐SNP (chr13_111,051,076_C/T) almost perfectly fixed for the mutant allele in the EAD population and TC breed, which completely removed methylation status in TC due to the transition from C to T allele (Figure 7E). We discovered that the SNP created a new TF binding site of TBX21 via the alternate T allele by motif scan analysis. Subsequent luciferase reporter assays revealed higher enhancer activity for the T allele, which might be the critical contributor delaying the expression of *GHSR* in TC (Figure 7F). The estimated allele substitution effect of this SNP for SW at Day 240 was 4.22 kg, and the individuals carrying one or two copies of the dominant T allele showed a significantly lower SW (Figure 7G). Based on publicly available Hi‐C data of porcine skeletal muscle, we revealed that the CpG‐SNP was likely to regulate the expression of the *GHSR* gene since both are located in the same topologically associated domain (TAD) (Figure 7H). Additionally, we also proposed that the heterochronic candidates, *CD36* and *NCAPG*‐*LCORL* cluster [10, 40, 41, 42], were pivotal contributors to skeletal muscle development and SW in pigs based on similar strategies (Figures S25–S28).

## DISCUSSION

Following domestication, long‐term artificial selection contributed to the formation of many pig breeds with spectacular phenotypic diversity. In the past few decades, modern breeding approaches imposed intense selective pressures on pig breeds such as Duroc, Landrace, and Large White for the improvement of desirable traits. These commercial lean‐type breeds were primarily sculpted for superior carcass yield and fast growth rate, while Eastern pig breeds (mainly Chinese) were renowned for excellent meat quality, high‐fat content, and early maturity. However, the genetic architecture behind phenotypic variation, especially the effects on morphogenesis, was not yet fully understood. Our genome‐wide screen for selective sweeps based on nine high‐quality genomes and 1081 individuals, the most extensive data set so far, enabled exploiting the contributions of coding and regulatory signatures on several important traits, particularly skeletal muscle development. The current work not only establishes a rich resource to probe functional variants based on genomic sequences, gene expression, and DNA methylation data but also could advance our understanding of the mechanistic basis of how artificial selection determines phenotypic changes by reshaping the pig genome, transcriptome, and epigenome.

A growing number of studies have explored genome‐wide selective sweep signatures during domestication and breeding in pigs [2, 3, 4, 9, 43], but the number of breeds and sample sizes in these studies remained limited. With the nine assembled genomes and 1081 individuals, including 78 diverse domestic breeds presented here, we furnished a comprehensive pan‐genome and genetic variation resources, and significantly expanded the catalog of current genetic information. Our work provides further support for phenotypic and genetic divergence between Eastern and Western pig populations. Intensive breeding practice in several Western breeds consolidates more concentrated regions, whereas selective sweeps in the Eastern pigs showed more dispersive patterns across the genome, which might endured less selective pressure [2]. Besides, EAD and WED pigs only shared a very small number of swept regions and genes, supporting the relatively independent process of domestication and breeding of these two categories [1]. The rich repertoire of promising candidates provides a valuable resource for the research community and future genome‐designed breeding in pigs.

The enrichment/depletion analysis for all putative genetic variations exhibited a significant depletion within exons, indicating strong signals of purifying selection on coding polymorphisms, especially missense mutations with potentially deleterious effects. However, many coding loci were consciously kept and further dispersed in the populations by modern breeding procedures, benefiting from their favorable consequences on several traits of economic interests, like causative mutations in the *MC1R*, *NR6A1*, *PRKAG3*, *RYR1*, and *SERPINA6* genes [9, 27, 30, 44, 45]. In addition to these well‐known genes, we identified two promising candidate genes, *BRCA1* and *ABCA3*, by bioinformatic and experimental approaches. The *BRCA1* gene codes for a tumor suppressor whose mutations in humans were associated with a predisposition to breast and ovarian cancers due to abnormal cell cycle pathways [46, 47]. We observed remarkably strong signals of selective sweeps in the *BRCA1* gene in the WED group. Interestingly, *BRCA1* was also shown to be under selection during chicken domestication, especially in commercial broiler breeding [48]. This consistent finding reflected the convergent patterns in the human‐driven selection process on specific preferred breeding goals for farm animals, like higher meat production and faster muscle growth. Porcine enzootic pneumonia (PEP) is a chronic and highly contagious respiratory disease that causes considerable economic losses and is primarily engendered by the Mhp [34, 35]. Generally, Chinese local breeds showed a higher susceptibility to Mhp than Western lean‐type breeds [34]. Our results indicated that the mutant T allele results in a nonsynonymous substitution leading to reduced alveolar macrophage and further attenuated host defense, which was supported by in vitro Mhp infection experiments in cell culture. Besides, we also identified several swept genes with coding mutations, such as *ALS2*, *EXOC2*, *PRDM15*, and *SEMA3B/E*, which played an essential role in muscle growth, fat deposition, and neuronal development [10, 49, 50, 51]. Altogether, these functional coding variants constitute valuable information for future pig breeding.

Divergent artificial selection has resulted in a tremendous difference in meat production between Chinese native and Western lean‐type pig breeds, but the developmental programs underlying this discrepancy were unclear. We reasoned that the phenotypic consequence was attributed to the timing of the myogenesis process. Heterochrony was broadly defined as the genetically controlled changes in the rate or timing of developmental events in an organism compared with its ancestors or other organisms, which could lead to a tremendous difference in size, shape, and characteristics of certain organs and features [20, 37, 52]. A variety of studies have explored the molecular heterochrony, mainly in the brain and retina across several species and identified numerous hub genes driving organic development, morphogenesis, and evolution [53, 54, 55, 56, 57]. It has been widely accepted that muscle growth can be divided into two major periods: prenatal myogenesis determined by an increase in myofibers number (hyperplasia), and postnatal growth, which was achieved by augmenting the size (hypertrophy) of myofibers [58]. Despite the high similarity, our results demonstrated that LDR shows a relatively more prolonged timing of myofiber formation in the prenatal stages than TC, but a faster muscle growth and greater lean accretion in the postnatal period. We found more DPGs and greater overlap with putative‐swept candidates in the prenatal stages, implying that the effects of artificial selection on skeletal muscle development can be traced back to the very early stages of myogenesis. Together, our data suggested that the vast difference in pork production between fat‐ and lean‐type pig breeds was attributed to the heterochrony in skeletal muscle development, and artificial selection had served as the crucial driving force in muscle growth, especially for prenatal hyperplasia.

DNA methylation was an epigenetic mark frequently occurring at CpG dinucleotides which plays an important role in shaping phenotypic diversity in the domestication of animals [59, 60]. In line with previous findings [59, 60, 61, 62], we found that Landrace pigs displayed more hypermethylated regions than TC pigs, resulting from extensive breeding. It appears that selective sweeps are more likely to overlap with DMRs detected at multiple stages, implying that surrounding fixed alleles might have a persistent effect on DNA methylation variation. Additionally, heterochronic genes, especially those covered by selective sweeps, were prone to be modulated by DMRs, suggesting sites with differential methylation levels were vital for controlling developmental progression. This finding provided novel insight into the spatial‐temporal patterns behind how artificial selection sculpts organogenesis by reshaping the epigenome.

In general, methylated cytosines exhibited a higher mutation rate, and many studies have reported a significant association between CpG to TpG/CpA transitions and developmental disorders [63, 64]. The majority of genetic underpinnings under selection were located within noncoding regions and generally act as regulatory elements to govern gene expression [65, 66, 67]. We identified an insertion as a potential enhancer regulating *BDH1* in Eastern pigs, which resulted in the difference in myofiber proliferation and differentiation between Eastern and Western breeds. BDH1 was an important rate‐limiting enzyme responsible for ketone metabolism and ATP synthesis [68, 69]. The higher expression of *BDH1* induced by the insertion might play a key role in myofiber proliferation and differentiation. Besides SVs, the present study shows that (nearly) fixed SNPs were important sources for interbreed differences in methylation patterns and can serve as regulatory sequence variants correlated with traits of interest. As a strong candidate under selection, *GHSR* was commonly associated with growth and carcass traits in livestock [38, 39], which was also supported by our association results of the regulatory SNP (chr13_111,051,076_C/T). Furthermore, higher GHSR and Ghrelin concentrations during pregnancy would lead to higher fetal weight and postnatal weight gain [70, 71, 72]. Interestingly, we found that the *GHSR* gene displayed a more advanced expression pattern only in the prenatal stage in LDR pigs, which may be the result of altered DNA methylation status. This finding means that administration of ghrelin happens at the earlier pregnancy and lasts longer in LDR, hence resulting in greater muscle mass and body weight. These data demonstrate the importance of pigs as a biomedical model to probe molecular mechanisms underlying some human metabolic disorders such as obesity and diabetes.

Despite the comprehensive nature of our study, several limitations should be acknowledged. First, the reliance on a limited number of genomes, though high‐quality, may not fully capture the genetic diversity present in all pig breeds. Second, while we integrated multiple omics data, the complexity of gene regulation means that additional factors, such as environmental influences and epigenetic modifications beyond DNA methylation, may also play significant roles in phenotypic differentiation but were not addressed in detail. Furthermore, the functional validation of identified regulatory elements and their impact on traits remains to be explored in future studies.

## CONCLUSION

In summary, our study elucidates the genetic basis of complex traits and phenotypic differentiation in pigs, revealing critical insights into the roles of noncoding regions and specific genes such as *BDH1* and *GHSR*. We identified significant SVs and SNPs that contribute to interbreed differences in traits of interest, particularly in myofiber proliferation and differentiation. The integration of genomic, transcriptomic, and methylomic data highlights the regulatory mechanisms underlying these phenotypic variations. These findings not only enhance our understanding of pig genetics but also offer valuable resources for genetic improvement programs aimed at optimizing traits in livestock.

## METHODS

### Sample collection and sequencing

To capture the full genetic diversity of pigs around the world, we collected the ear tissues of 313 individuals from 30 distinct breeds, representing populations at the climatic and geographical extremes of China. Genomic DNA was extracted from the ear tissues of 313 samples using a standard phenol‐chloroform method. About 1.5 μg DNA was used to construct an approximately 350 bp insert‐size DNA library at Novogene. In brief, the DNA sample was fragmented, and then the ends were repaired and ligated to the adaptor. Next, adapter‐ligated DNA was selected by running a 2% agarose gel to recover the target fragments. Polymerase chain reaction (PCR) amplification and purification were then performed. According to the standard manufacturer's instructions, the quantified library was sequenced on the Illumina NovaSeq platform (Illumina). Sequencing data for a total of 98 individuals from 11 native breeds were shared by our collaborators [73, 74, 75, 76]. In addition, we downloaded 670 high‐depth whole‐genome sequencing data from the Sequence Read Archive (SRA) database (accessed on 2020/09/12) (Table S12) to establish the comprehensive pig genome sequence data set.

A total of 81 skeletal muscle (*longissimus dorsi*) samples were newly collected from TC pigs at 27 developmental stages, including embryonic days 33, 40, 45, 50, 55, 60, 65, 70, 75, 80, 85, 90, 95, 100, and 105 (abbreviated as E33, E40, E45, E50, E55, E60, E65, E70, E75, E80, E85, E90, E95, E100, and E105) and postnatal days 0, 9, 20, 30, 40, 60, 80, 100, 120, 140, 160, and 180 (abbreviated as D0, D9, D20, D30, D40, D60, D80, D100, D120, D140, D160, and D180). Total RNA for RNA‐seq and DNA for WGBS were extracted for the 81 samples using the standard procedures and were sequenced on the Illumina HiSeq X Ten platform (Illumina). Both WGBS and RNA‐seq were performed for three biological replicates for each developmental stage. The time‐course transcriptome (81 samples) and methylome (81 samples) data from LDR pigs were obtained from our previous study [77]. In total, 162 transcriptome and 162 DNA methylation data were used to study skeletal muscle growth and development at 27 stages. It is worth noting that the Landrace and Tongcheng pigs were raised in the same pig farm, with the same feeding environment, feeding feed, and management mode.

### Genome assembly

The genomic DNA libraries from seven individuals (one AW, three Chinese indigenous pig breeds [BMA, TT, and LT], and three Western pig breeds [LW, BKS, and PTR]) were constructed and sequenced using the PacBio Sequel platform with circular consensus sequencing (CCS) mode. Hifiasm (v0.16.1_r375) [78] was used to generate the assembly from HiFi CCS reads using default parameters. Hi‐C fragment libraries from the same samples were generated with insert sizes of 300–700 bp and sequenced on the Illumina platform. The enzyme used in the Hi‐C library was DpnII which cuts DNA at “GATC.” Adapter sequences of raw reads were trimmed, and low‐quality paired‐end reads were removed for clean reads by using the fastp (v0.19.5) [79] program. Bowtie2 (v2.2.9) [80] was used to align the clean reads to the assembled contigs. We filtered low‐quality reads using a HiC‐Pro pipeline (v3.1.0) [81] with the default parameters. The valid reads were used to anchor chromosomes with Juicer (v1.6) [82] and 3D‐DNA pipeline (v180419) [83]. The assembly completeness was assessed using the Benchmarking Universal Single‐Copy Orthologs (BUSCO) program (v5.3.2) [84] containing the Mammalia odb10 gene set (9226 BUSCO genes). The single‐copy plus duplicated complete BUSCO gene counts were reported. Using a similar method in the nearly complete sheep genome study [85], Merqury (v1.3) with 21‐mer was applied to calculate the quality value (QV) [86].

### Pan‐genome construction

Seven HiFi assemblies and our previous Luchuan genome were aligned to the Duroc pig reference genome (Sscrofa11.1) by employing minimap2 (‐a ‐x asm10) (v2.24‐r1122) [87]. A reliable alignment was defined as a continuous alignment longer than 300 bp with sequence identity higher than 90%. Sequences with no reliable alignments were kept as unaligned sequences. Next, MUMmer (v4.0.0rc1) [88] was used to map unaligned sequences to the Sscrofa11.1 genome using the parameters ‐maxmatch and then used the delta‐filter ‐I 90 ‐l 300 ‐1 parameter with the one‐to‐one alignment block option to filter the alignment results. The resulting sequences were aligned against the Sscrofa11.1 genome using blastn (with the parameters ‐word_size 20, ‐max_hsps 1, ‐max_target_seqs. 1, ‐dust no, ‐soft_masking false, and ‐evalue 0.00001) (v2.9.0) [89], and the sequences showing identity >90% to Sscrofa11.1 sequences were removed. The remaining sequences were merged according to the adjacent regions within 200 bp, and sequences of <500 bp in length were removed. Next, the generated sequences were aligned to the GenBank nt database with BLASTN (v2.9.0) [89] with parameters “‐evalue 1e‐5 ‐best_hit_overhang 0.25 ‐perc_identity 0.5 ‐max_target_seqs. 1.” Sequences with best hits from other species, or covered by known animal mitochondrial genomes, were possible contaminations and removed. Subsequently, the remaining sequences obtained from all the assemblies were combined. To remove redundancies, all‐versus‐all alignments with minimap2 (v2.24‐r1122) [87] (‐X ‐a ‐x asm10) were carried out.

### Annotation of TEs

The TEs were identified using two methods: de novo repeat identification and known repeat searching against existing databases. RepeatModeler (v2.0.1) was used to predict repeat sequences in the genome and RepeatMasker (v4.0.7) [90] was then used to search the genome against the de novo TE library. RepeatMasker (v4.0.7) [90] and the Repbase (v15.02) [91] database were used to identify known TE repeats in the assembled genome. RepeatMasker was applied for DNA‐level identification and RepeatProteinMasker was used to perform protein‐level identification. The annotation we used here is ensembl release‐94. We adopted a similar method described in the Landrace pig study to identify the telomeres within the seven newly created genomes [92]. The centromeric repeat regions in the expected chromosomal locations were obtained based on previous analyses [93]. In cases where the centromere regions could not be determined through homologous alignment, we employed the quartTeT (v1.1.8) method for identification [94].

### Gene prediction

For the homolog prediction method, the genome sequences and annotation files from six mammalian genomes, including humans, mice, cattles, dogs, goats, and Duroc pigs, were downloaded from the Ensembl database (Ensembl release 105). Besides, the Luchuan pig protein sequences were downloaded from the China National GenBank (CNGB; https://db.cngb.org/) under the accession of CNP0001159. These sequences were aligned to the genome assembly using GeMoMa (v1.8) [95] to detect homologous peptides. For the RNA‐seq‐based prediction approach, the raw Illumina short reads of nine tissues were used, as well as one pool RNA library (i.e., subcutaneous adipose, kidney, heart, lung, longissimus dorsi muscle, liver, psoas major muscle, spleen, ovary. Accession numbers SRR3160015, SRR3160012, SRR3160008, SRR3160011, SRR3160014, SRR3160009, SRR3160017, SRR3160013, SRR3160010, and SRR3160016 were downloaded from NCBI for further analyses. All raw reads were assessed using fastp (v0.19.5) [79] with the default setting. The clean reads were aligned to the genome assembly using HISAT2 (v2.0.1) [96] to identify putative exon regions and splice junctions. StringTie (v1.2.2) [97] was then used to assemble the mapped reads into gene models and validated by Program to Assemble Spliced Alignment (PASA) (v2.4.1) [98]. Genes that had PASA support with correct structure but lost in homology‐based prediction were added to the gene set. Finally, untranslated regions and alternative splicing regions were determined using PASA [99].

### Gene functional annotation

Five public databases, including NCBI nonredundant protein sequence database (accessed on 2022/02/14), SwissProt (accessed on 2022/10/12) [100], Kyoto Encyclopedia of Genes and Genomes (KEGG, 84.0) [101], Translation of European Molecular Biology Laboratory (accessed on 2022/10/12), and GO, were used for functional annotation of the reference gene set. Putative domains and GO terms of these genes were identified using InterProScan (v20221014‐5.59‐91.0) [102], while the Diamond program (v0.9.30) [103] was used to compare the protein sequences of the pig genome against the remaining four public databases with an *E*‐value cutoff of 1e‐05.

### SV detection

We employed a similar method as described in Munasinghe et al. [104] to detect SVs. Briefly, AnchorWave (v1.0.1) [22] was used to compare each of the eight pig genomes (AW, BMA, BKS, LT, LC, LW, PTR, and TT) to Duroc pig (Sscrofa11.1) via the “genoAli” command and “‐IV” parameter. The output *.maf* files were converted to *.sam* format with maf‐convert.py (https://gitlab.com/mcfrith/last/-/blob/main/bin/maf-convert). Finally, samtools mpileup (v1.10) [105] was applied to call SVs.

### Reads mapping of whole‐genome sequencing data to the pan‐genome

We merged putative nonredundant nonreference sequences and the Sscrofa11.1 reference assembly as the pig pan‐genome. The fastq reads were first trimmed by fastp (v0.19.5) [79] with default parameters. Next, all clean reads, including our newly generated samples, were aligned to the pan‐genome using the BWA‐MEM pipeline (v0.7.17‐r1188). The mapped reads were then sorted, and duplicates were removed by Picard tools (v2.23.3) and SAMtools (v1.10) [105].

### Genome‐wide screening of SNPs and INDELs

The genome‐wide variants were called for each sample by the GATK UnifiedGenotyper (v3.7‐0‐gcfedb67) [106] with *‐glm BOTH ‐rf BadCigar ‐‐sample_ploidy 2* option. To ensure high accuracy of variants calling, SNPs with *QD* < 2.0 | | *FS* > 60.0 | | *MQ* < 20.0 || *MQRankSum* < −12.5 || ReadPosRankSum < −8.0 were filtered. Gene‐based SNP annotation was performed according to the annotation of the pan‐genome using the package ANNOVAR (v2013‐06‐21) [107] and Ensembl VEP [108]. Based on the genome annotation, SNPs were categorized as occurring in exonic regions, 5′ or 3′ untranslated regions, intronic regions, splicing sites (within 2 bp of a splicing junction), upstream and downstream regions (within a 1 kb region upstream or downstream from the transcription start site [TSS]), or intergenic regions. SNPs in coding exons were further grouped as either synonymous SNPs or nonsynonymous SNPs. To assess the sequence conservation of missense mutations, we first downloaded the amino acid sequences of nine related mammals from the NCBI or Ensembl databases, including humans, mice, cattles, horses, goats, tigers, cats, dogs, and rabbits. Subsequently, multiple sequence alignments were performed by the MUSCLE program [109]. Meanwhile, the SWISS‐MODEL workspace [110] was used to predict protein structure models for wild‐type and mutant variants.

### Phylogenetic and population genetic analyses

To provide insight into phylogenetic relationships among different pig breeds, we performed a comprehensive genomic survey based on all autosomal high‐quality bi‐allelic SNPs with a call rate of ≥90% and a minor allele frequency of ≥5%. A neighbor‐joining tree was constructed using the program TreeBeST (http://treesoft.sourceforge.net/treebest.shtml) with 200 bootstrap replicates. The tree was displayed using Interactive Tree Of Life (iTOL) (v6) [111]. To infer the population structure, we used ADMIXTURE (v1.3.0) [112], which implements a block‐relaxation algorithm. We also filtered SNPs by testing Hardy–Weinberg equilibrium (HWE) violations (*p* > 10^−4^) and reconstructed the model‐based clustering analysis. To identify the best genetic clusters *K*, cross‐validation error was tested for each *K* value from 2 to 10. The PCA was conducted using the program GTAC (v1.92) [113].

### Selective sweeps and differentiation analysis

We first excluded the potential mixed samples between the Eastern and the Western pigs according to the results of population structure, to ensure the accuracy of the putative selective sweeps. When *K* = 2, if the lineage of a Western pig is more than 20% for an Eastern breed, it will be filtered out, and vice versa for Western breeds. SNPs with minor allele frequency below 5% were removed from this analysis. Subsequently, a computationally advanced CLR test was used to detect genome‐wide selective sweeps for all populations by SweeD software (v4.0.0) [114]. CLR scores were computed for each 10‐kb nonoverlapping window along all the autosomes. Potential selective sweeps between the wild population and the domesticated population were detected using the Pi ratio (πwild/πdomesticated, separate testing of Western and Eastern populations) and FST values between Eastern and Western pigs were obtained using VCFtools (v0.1.13) with a 50 kb sliding window and a step size of 10 kb. Windows containing less than 10 SNPs were excluded from further analysis. Windows in the top 1% values were considered to be candidate regions. For the top 1% CLR scores, overlapped windows between domesticated and wild populations will be removed. The results of each of the above methods were combined to study the domestication and differentiation of pigs. The linkage disequilibrium (LD) block sizes of candidate genes and regions were calculated by LDBlockShow (v1.30) [115].

To evaluate the association between candidate variants and traits of economic importance in pigs, we leveraged genotype and phenotype data of 589 F_2_ individuals from the previous study of our collaborator, which was derived from a cross between 5 Large White (European, WED) boars and 16 Min (Chinese, EAD) sows [32]. We first sorted the pedigree information according to the birth order by a custom Python script, and next established the relative kinship coefficients matrix. Finally, we compared the differences in the SW at 240 days and LMP using a mixed linear model (MLM) with the matrix as the covariate. The *p* value was corrected by the Bonferroni approach, and a corrected *p* value of less than 0.05 was regarded as significantly different. To classify functional categories of putative selective sweeps in detail, we estimated the Jaccard index between these swept regions and five genomic features (promoter, exon, intron, intergenic, and TE) with 1000 permutations. GO enrichment analysis of swept genes was implemented with the Metascape tool [116]. GO terms with corrected *p* values of <0.05 were considered significantly enriched. To explore enriched phenotypes driven by putative selection signatures, we performed trait/QTL enrichment analysis by a hypergeometric test against the pig QTL database [29]. We focused on the QTLs with confidence intervals less than 1 Mb, given that the QTL confidence intervals are too large to be used efficiently in the postprocessing.

### Transcriptome sequencing

Total RNA was extracted with TRIzol Reagent (Invitrogen). A total amount of 3 μg RNA per sample was used as input material for the RNA sample preparations. Ribosomal RNA was removed by Epicentre Ribo‐zeroTM rRNA Removal Kit (Epicentre), and rRNA‐free residue was cleaned up by ethanol precipitation. The RNA concentration was monitored with a Qubit Fluorometer (Invitrogen), and the RNA quality was evaluated by the Agilent Bioanalyzer 2100 system (Agilent Technologies) before library preparation. Subsequently, sequencing libraries were generated using the rRNA‐depleted RNA by NEBNext UltraTM Directional RNA Library Prep Kit for Illumina (NEB, MA) following the manufacturer's recommendations. Finally, paired‐end sequencing with 150 nucleotides at each end was implemented on the Illumina NovaSeq 6000 platform (Illumina).

### WGBS

A total amount of 100 ng genomic DNA spiked with 0.5 ng lambda DNA was fragmented by sonication to 200–300 bp with Covaris S220. These DNA fragments were treated with bisulfite using EZ DNA Methylation‐Gold™ Kit (Zymo Research), and the library was constructed by Novogene Corporation. Subsequently, library quality was assessed on the Agilent Bioanalyzer 2100 system, and 150 bp pair‐end sequencing of each sample was performed on the Illumina Novaseq 6000 platform (Illumina).

### RNA‐seq analysis

The raw data were filtered by fastp (v0.19.5) with default parameters [79]. The clean data were mapped to the constructed pan‐genome and annotation files with STAR aligner (v2.7.1) [117]. Gene‐level read counts were enumerated at the same time, and used as input for DESeq2 package (v1.38.3) [118]. Based on these normalized counts obtained using the rlogTransformation function in DESeq2 software, we conducted PCA, hierarchical clustering, and k‐means clustering. Subsequently, we applied the TimeMeter tool (v1.0.5) [37] to evaluate temporal gene expression similarity and detect DPGs between LDR and TC breeds. PAS indicated faster expression patterns in TC (TC acceleration genes), while negative means accelerated changes in LDR (LDR acceleration genes). GO term of biological process analysis predicted with differentially expressed genes (DEGs) was conducted with the Metascape [116].

### WGBS analysis

Our constructed pan‐genome was first transformed into a bisulfite genome using the Bismark tool (https://www.bioinformatics.babraham.ac.uk/projects/bismark/). Then the clean data after quality control were aligned to the bisulfite genome with Bismark (v0.22.0) based on the default parameters. The methylation information for each cytosine site was extracted after filtering the duplicate reads. Differentially methylated cytosine sites (DMCs) and DMRs were identified using MethylKit software (v0.99.2) [119]. For the DMC analysis, the cytosine sites with coverage of less than 10 were removed. A sliding window approach was used to calculate DMRs, in which both the window and the step were set to 1000 bp, cytosine sites with coverage less than five were removed and regions that contained at least three cytosine sites were left for the downstream analyses. Both the DMCs and DMRs were defined with the criterion of Bonferroni correction *q* value of <0.05 and meth.diff >30. We focused on the gene body region (from TSS to transcription end site [TES]), promoter region (upstream and downstream 2 kb from the TSS), and transposon element to assess the methylated information annotation.

### Correlation analysis between gene expression and DNA methylation

Before correlation analysis, we removed genes with rlog‐normalized counts less than zero and more than 20. Then, the DNA methylation levels in the gene body and promoter region for each expressed gene were computed. The Pearson's correlation coefficients (*r*) between DNA methylation levels and gene expression of different features were calculated in R software (v4.2.1).

### Motif scan analysis

Motifs scan analysis was performed with the findMotifsGenome.pl function under the default parameters in Homer software (v4.11). We took the SNPs and their upstream and downstream 10 kb as regions to make a bed file and then performed motif scan analysis based on the sequence in the genome.

### In vitro adipocyte proliferation assays

The subcutaneous adipose tissues from the neck and back of newborn pigs were collected under aseptic conditions and washed three times with PBS buffer containing high concentrations of penicillin and streptomycin. Visible blood vessels and connective tissue were removed. The adipose tissue was then cut into 1 mm³ pieces and digested with type I collagenase solution (800 U/mL) for 1 h at 37°C, with agitation every 5 min. The adipose tissue digestate was filtered through a double‐layer nylon sieve (100 μm and 25 μm), and the filtrate was centrifuged at 1500 r/min for 10 min to obtain preliminary pig adipocytes. The adipocytes were resuspended in serum‐free DMEM/F12 (Dulbecco's Modified Eagle Medium) medium and centrifuged again at 1000 r/min for 5 min to remove any residual digestate. Finally, the adipocyte pellet was resuspended in complete medium (DMEM/F12 90% + Fatal Bovine Serum [FBS, Gibco] 10% + Penicillin–Streptomycin [PS, Thermo Scientific]) and seeded into T25 culture flasks for further growth. For the proliferation analysis of adipocytes, two key methods were employed: EDU incorporation and qRT‐PCR. The EDU (5‐ethynyl‐2’‐deoxyuridine) assay was used to measure DNA synthesis, which serves as an indicator of cell proliferation. In addition to EDU, qRT‐PCR was employed to quantify the expression levels of proliferation‐related genes.

### Cell culture

Mouse C2C12 myoblasts and HEK‐293T cells were obtained from Peking Union Medical College Hospital and cultured in Dulbecco's Modified Eagle Medium (DMEM, Gibco) supplemented with 10% FBS and 1% PS (Thermo Scientific). In addition, porcine adipocyte was cultured in DMEM/F12 supplemented with 10% FBS and 1% PS (Thermo Scientific). All the cells were cultured at 37°C in a 5% CO_2_ incubator. Specifically, C2C12 myoblasts differentiation was induced by replacing the growth medium with differentiation medium (DMEM supplemented with 2% horse serum) when the cells reached approximately 80%–90% confluence. This medium change was performed every 48 h for 4–6 days. To monitor the effect of differentiation, we used both morphological and molecular markers. Morphological changes were assessed through the appearance of elongated, multinucleated myotubes, which is a key indicator of successful differentiation. At the molecular level, we monitored the expression of myogenic markers such as Myogenin and Myosin Heavy Chain (MHC) via qRT‐PCR, which are widely recognized as markers of C2C12 differentiation.

### Plasmid construction

The full‐length CDS regions of the mouse *CD36*, *GHSR*, and *NCAPG* genes were amplified by PCR. The total reaction volume is 50 µL, containing 2.5 µL template DNA, 2.5 µL forward and reverse primers containing BamH I and Xho I sites, 25 µL of 2× Vazyme LAmp Master Mix (Vazyme) and 17.5 µL nuclease‐free water. The PCR program includes an initial denaturation at 95°C for 5 min, followed by 35 amplification cycles consisting of 95°C for 30 s (denaturation), 56°C for 30 s (annealing), and 72°C for 1 min (extension). The reaction ends with a final extension at 72°C for 7 min to ensure complete synthesis of PCR products, which are then analyzed using agarose gel electrophoresis to confirm the correct size and presence of the amplified fragment.

After PCR amplification, the products were subjected to double digestion using BamH I and Xho I (Thermo Fisher Scientific). The digestion was performed in a total reaction volume of 50 µL, containing 10 µL of PCR product (approximately 0.5–1 µg), 1X reaction buffer compatible with both enzymes (as provided by the manufacturer), 1 µL (10 units) of each enzyme, and nuclease‐free water. The reaction mixture was incubated at 37°C for 2 h, according to the protocol of manufacturers. Following digestion, the products were purified using a MolPure® PCR Purification Kit (YESEN) before further cloning. After purification of the digested products and vector (pcDNA3.1(+) vector [Invitrogen]), a ligation reaction is set up with a molar ratio of 1:3 (vector:insert). The total reaction volume is 20 µL, containing approximately 50–100 ng of vector DNA, the corresponding amount of insert DNA calculated based on the molar ratio, 2 µL of 10X T4 DNA ligase buffer (containing ATP), and 1 µL (400 units) of T4 DNA ligase (Thermo Fisher Scientific). The reaction mixture is incubated at 4°C overnight. Following the ligation, the product is transformed into *Escherichia coli* cells for subsequent screening.

To verify the dual‐luciferase activity of candidate SNPs and insertion from the *CD36*, *GHSR*, *NCAPG*, and *BDH1* genes, 400‐bp DNA fragments centering on the SNPs and insertion were first amplified and then inserted into the PGL4.23 Dual‐Luciferase Expression Vector (Promega). Primers were designed using Primer Premier 5 and listed in Table S31.

### RNA interference

Based on the CDS region of the target gene provided by NCBI, siRNAs of the target gene were designed using the siDirect website (http://sidirect2.rnai.jp/) and synthesized at GenePharma. The siRNA sequences are listed in Table S32.

### Cell transfection and Dual‐Luciferase Reporter Assay

After 12 h of cell culture, the C2C12 myoblasts or HEK293T cells were transfected with the appropriate plasmids or oligos using Lipofectamine 3000 and Opti‐MEM according to the manufacturer's protocols. These cells were collected after a transfection period of 24 h. A Dual‐Luciferase Reporter Assay System (Promega) was used to quantify luciferase activities following the manufacturer's instructions. Firefly luciferase activity was normalized to Renilla luciferase activity.

### qRT‐PCR

For the expression analysis of *CD36*, *BRCA1*, *GHSR*, and *NCAPG*, total RNA was isolated from cultured cells. In the case of *BDH1* expression analysis, total RNA was isolated from different skeletal muscles (LDR, TC, and BM) of pigs as well as from cultured cells. Total RNA was isolated from cultured cells using TRIzol reagent (Invitrogen), and then reverse‐transcribed to cDNA using the PrimeScript™ RT Master Mix (Perfect Real Time) (Takara) according to the manufacturer's protocols. qRT‐PCR was performed on a StepONE Real‐Time PCR System (Applied Biosystems) according to the SYBR Premix Ex TaqTM instructions. Each reaction was performed in a 20 μL reaction mixture containing 10 μL of ChamQ SYBR qPCR Master Mix (Vazyme Q311‐02), 0.5 μL of gene‐specific primers, 2 μL of template cDNA, and 7 μL of sterile water. The qRT‐PCR was performed with the following conditions: initial denaturation at 95°C for 10 min, 40 cycles of denaturation at 95°C for 15 s, annealing at 60°C for 30 s, and extension at 72°C for 30 s, followed by a melt curve analysis. All reactions were repeated three times with cDNA from three independent individuals, and the results were analyzed using the $2^{-∆∆C_{T}}$ method, with normalization to a housekeeping gene.

### 5‐Ethynyl‐2′‐Deoxyuridine (EDU) assay

The C2C12 myoblasts and adipocytes were seeded in 12‐well plates. When the cells grew to a density of 50% confluence, they were transfected with overexpression plasmid, siRNA, or miRNA mimics. After transfection for 48 h, myoblasts were exposed to 50 μM EDU (RiboBio) for 2 h at 37°C. Subsequently, the cells were fixed in 4% paraformaldehyde for 30 min, neutralized using 2 mg/mL glycine solution, and then permeabilized by adding 0.5% Triton X‐100. A solution containing EDU (Apollo Reaction Cocktail; RiboBio) was added and the cells were incubated at room temperature for 30 min. The nuclear stain Hoechst 33342 was then added, and incubation was continued for another 30 min. A fluorescence microscope (DMi8, Leica) was used to capture three randomly selected fields to visualize the number of EDU‐staining cells.

### Immunofluorescence analysis

Cells were cultured and fixed in six‐well plates with paraformaldehyde, treated with 0.5% Triton, blocked with goat serum for 1 h, and incubated with anti‐MyHC (1:500, DSHB MF20) antibodies for 2 h. Next, cells were incubated with goat anti‐mouse secondary antibodies. Finally, DAPI (1:1000, Invitrogen D3571) was added, and the cells were observed using the Leica DMI3000 B microscope (Leica).

### AAV‐mediated in vivo overexpression of *GHSR* and *CD36* in mice

To validate in vivo the function of the *GHSR* and *CD36* genes, we first obtained adeno‐associated virus 9 (AAV9) serotypes of pcDNA3.1‐GHSR, pcDNA3.1‐CD36, or pcDNA3.1‐Control from HANBI. Meanwhile, 7‐week‐old mice were obtained from the company Huafukang. The tibialis anterior (TA) muscle of the right leg in each mouse was injected with AAV9 virus (100 µL at titer ≥1 × 10¹³ vg/mL) with pcDNA3.1‐GHSR or pcDNA3.1‐CD36, and the left TA muscle was injected with pcDNA3.1‐Control. The AAV9 vector‐treated mice in triplicate were anesthetized with isoflurane and killed by cervical dislocation to collect muscles. For *GHSR*, we collected TA muscles after 10 days of AAV9 injection. To verify accelerated muscle maturation regulated by *CD36*, we designed a muscle injury and regeneration model. We first injected the AAV9 virus overexpressing *CD36* or Control 10 days before cardiotoxin (CTX) injection and then collected TA muscles at Days 0, 1, 5, and 10 post‐CTX injections. After optimal cutting temperature embedding, frozen sections were fixed in 4% paraformaldehyde overnight, and H&E staining was carried out according to the Hematoxylin‐Eosin/HE Staining Kit (Solarbio).

### Mhp challenge experiments

The cell density of porcine alveolar macrophages (PAMs) was adjusted to 1  10^4^ cells per well in a 24‐well plate and cultured in 0.5 mL of RPMI 1640 medium supplemented with 10% FBS and 2% PS solution at 37°C. The cells were washed with PBS to remove any unattached cells after 12 h of culture and then transfected with 0.4 μg DNA using QIAGEN Transfection Reagent (Cat. No. 301005) according to the manufacturer's instructions. After 24 h transfection, the cells were transfected again. Subsequently, PAMs with wild‐type and mutant alleles were treated by Mhp. After 6 h incubation, these infected cells were harvested for qRT‐PCR. The PAMs and Mhp materials were from the Institute of Veterinary Medicine, Jiangsu Academy of Agricultural Sciences.

### Statistical analysis

Statistical analyses were performed using R (v4.2.1) and GraphPad Prism (v9). Permutation tests were performed to evaluate the enrichment of selection signatures across different genomic regions. Enrichment analysis was carried out using permutation tests to identify the association between DMRs with sustained changes and selective sweeps in both prenatal and postnatal periods. Pearson correlation analysis between gene expression levels and DNA methylation patterns was conducted using the “cor” function in R, with the “Pearson” method. The statistical method used for the expression difference between genes with and without structural variants in multiple tissues was the Wilcoxon test. For LMP and SW at Day 240, statistical comparisons among multiple groups were made using one‐way analysis of variance (ANOVA). For cell assays, qRT‐PCR results, EDU, and immunofluorescence, the significance of differences between the two groups was determined using the Student's *t* test. Statistical significance was determined at *p < 0.05, **p < 0.01, and ***p < 0.001.

## AUTHOR CONTRIBUTIONS

**Lei Liu**: Investigation; funding acquisition; writing—original draft; visualization; formal analysis; project administration; data curation. **Guoqiang Yi**: Conceptualization; funding acquisition; writing—original draft; visualization; formal analysis; project administration; data curation; supervision. **Yilong Yao**: Investigation; writing—original draft; validation; visualization; formal analysis. **Yuwen Liu**: Investigation; writing—review and editing; visualization; formal analysis. **Jiang Li**: Investigation; writing—original draft; visualization; formal analysis; data curation. **Yalan Yang**: Visualization; formal analysis. **Mei Liu**: Writing—review and editing. **Lingzhao Fang**: Writing—review and editing. **Delin Mo**: Investigation. **Longchao Zhang**: Investigation. **Yonggang Liu**: Investigation. **Yongchao Niu**: Writing—original draft; visualization; data curation. **Liyuan Wang**: Validation. **Xiaolu Qu**: Validation. **Zhangyuan Pan**: Formal analysis. **Lei Wang**: Formal analysis. **Muya Chen**: Formal analysis. **Xinhao Fan**: Formal analysis. **Yun Chen**: Validation. **Yongsheng Zhang**: Validation. **Xingzheng Li**: Formal analysis. **Zhen Wang**: Formal analysis. **Yijie Tang**: Investigation. **Hetian Huang**: Formal analysis. **Pengxiang Yuan**: Formal analysis. **Yuying Liao**: Investigation. **Xinjian Li**: Investigation. **Zongjun Yin**: Investigation. **Di Liu**: Investigation. **Dongjie Zhang**: Investigation. **Quanyong Zhou**: Investigation. **Wangjun Wu**: Investigation. **Jicai Jiang**: Writing—review and editing. **Yahui Gao**: Writing—review and editing. **George E Liu**: Writing—review and editing. **Lixian Wang**: Investigation. **Yaosheng Chen**: Investigation. **Kui Li**: Writing—review and editing. **Martien A. M. Groenen**: Writing—review and editing; supervision. **Zhonglin Tang**: Conceptualization; funding acquisition; writing—review and editing; project administration; resources; supervision.

## CONFLICT OF INTEREST STATEMENT

The authors declare no conflict of interest.

## ETHICS STATEMENT

All animals used in the present study were handled in compliance with the guidelines (AGIS2020.04.17) provided by the Biomedical Research Ethics Committee of the Agricultural Genomics Institute at Shenzhen, Chinese Academy of Agricultural Sciences.

## Supporting information


**Figure S1.** The comprehensive analysis pipeline used in this study.
**Figure S2.** Snail plots showing assembly statistics of six HiFi genomes.
**Figure S3.** Dotplots showing patterns of synteny and collinearity between assembled genomes and Sscrofa11.1 reference.
**Figure S4.** Genome‐wide structural variants.
**Figure S5.** Global map of genetic variations in the pig genome.
**Figure S6.** Examples of swept genes in EAD and WED pigs and functional enrichment results.
**Figure S7.** Functional exploration of *NR6A1* gene.
**Figure S8.** Functional study of the *BRCA1* variant.
**Figure S9.** Distinct genomic landscape and functions of the coding variant in the *ABCA3* gene.
**Figure S10.** Global transcriptome profiles across skeletal muscle development in Landrace (LDR) and Tongcheng (TC) pigs.
**Figure S11.** Integrative analysis of transcriptome data from Landrace (LDR) and Tongcheng (TC) pigs.
**Figure S12.** Gene expression modules affected by selective sweeps.
**Figure S13.** Comparison of skeletal muscle development in Landrace (LDR) and Tongcheng (TC) pigs.
**Figure S14.** Significant differences in the expression levels between all expressed genes and the swept genes in EAD and WED groups.
**Figure S15.** Distribution of DNA methylation profiles across the 27 skeletal muscle developmental stages in Landrace (LDR) and Tongcheng (TC) pigs.
**Figure S16.** Comparisons of DNA methylation levels between Landrace (LDR) and Tongcheng (TC) pigs.
**Figure S17.** Correlation between gene expression and DNA methylation levels of promoter regions in Landrace pig.
**Figure S18.** Correlation between gene expression and DNA methylation levels of gene body regions in Landrace pig.
**Figure S19.** Correlation between gene expression and DNA methylation levels of promoter regions in Tongcheng pig.
**Figure S20.** Correlation between gene expression and DNA methylation levels of gene body regions in Tongcheng pig.
**Figure S21.** Changed correlation patterns between gene expression and DNA methylation levels by selective sweeps.
**Figure S22.** Comparison of the DNA methylation levels in promoter regions for all genes and swept genes.
**Figure S23.** Comparison of the DNA methylation levels in gene body regions for all genes and swept genes.
**Figure S24.** Cell differentiation assessment for the expression levels of *PCNA*, *Ki67*, *MyoG*, and *MyHC* marker upon *BDH1* knockdown by qRT‐PCR analysis in C2C12 cells.
**Figure S25.** Enhanced capacity for cell proliferation of *GHSR* gene.
**Figure S26.** Comprehensive analysis of the *CD36* gene associated with skeletal muscle development and meat performance.
**Figure S27.** Enhanced capacity for cell differentiation of *CD36* gene.
**Figure S28.** Comprehensive functional analysis of *NCAPG*‐*LCORL* locus.

**Table S1.** Summary of sequencing data generated in this study.
**Table S2.** Statistics of the contigs generated by hifiasm software.
**Table S3.** Statistics for the individual pig genomes.
**Table S4.** Transposable elements in the seven individual pig genomes determined using various software.
**Table S5.** The identified telomeres in seven newly generated pig genomes.
**Table S6.** The characteristic of centromeres in seven newly generated pig genomes.
**Table S7.** The number of genes with homology or functional classification using each annotation method.
**Table S8.** The nine genomes used in constructing pan‐sequences.
**Table S9.** The identifications of QV in seven newly generated pig genomes.
**Table S10.** The characteristics of novel sequences in this study.
**Table S11.** The number of novel genes with homology or functional classification using each annotation method.
**Table S12.** The summary of publicly available whole‐genome sequencing data.
**Table S13.** The number of breeds and sample size per breed of 1081 pigs.
**Table S14.** The sequencing quality and read alignment statistic of whole‐genome sequencing data from 1081 individuals.
**Table S15.** The annotation summary of SNPs and INDELs detected in 1081 samples.
**Table S16.** List of genes in the EAD‐specific sweep regions.
**Table S17.** List of genes in the WED‐specific sweep regions.
**Table S18.** List of selected genes identified by fixation index (FST) method (EAS vs WES).
**Table S19.** List of selected genes identified by the nucleotide diversity (Pi) method (EAW vs EAD).
**Table S20.** List of selected genes identified by the nucleotide diversity (Pi) method (WEW vs WED).
**Table S21.** List of overlapped genes between Eastern and Western domestication using the CLR method.
**Table S22.** List of overlapped selected genes between EAW vs EAD and WEW vs WED using the nucleotide diversity (Pi) method.
**Table S23.** Enrichment analysis of QTLs in EAD group.
**Table S24.** Enrichment analysis of QTLs in WED group.
**Table S25.** Annotation summary of coding mutations by the Ensembl Variant Effect Predictor.
**Table S26.** Mapping statistics of time‐course RNA‐seq data in Landrace and Tongcheng.
**Table S27.** Differentially progressing genes between Tongcheng and Landrace during prenatal skeletal muscle development.
**Table S28.** Differentially progressing genes between Tongcheng and Landrace during postnatal skeletal muscle development.
**Table S29.** Summary statistics of time‐course methylation profiles.
**Table S30.** The number of differentially methylated cytosines and regions between Tongcheng and Landrace at each stage.
**Table S31.** Primers used in this study.
**Table S32.** siRNA sequences used in this study.

## Data Availability

Raw sequencing reads newly generated in this work, including whole‐genome sequencing of 313 individuals, 81 RNA‐seq, and 81 WGBS data for Tongcheng pigs at 27 developmental stages, as well as HiFi and Hi‐C data for seven pig breeds, were deposited in the National Center for Biotechnology Information (NCBI) database under the accession number PRJNA754250 (https://dataview.ncbi.nlm.nih.gov/object/PRJNA754250?reviewer=cvg3ve2gvba6ggsds5ck12me0t). The 81 RNA‐seq and 81 WGBS data for Landrace pigs at 27 developmental stages were obtained from our previous study under accession number GSE157045 in NCBI (https://www.ncbi.nlm.nih.gov/gds?LinkName=bioproject_gds&from_uid=660337). In addition, other sequencing data in this study are downloaded from NCBI Gene Expression Omnibus (https://www.ncbi.nlm.nih.gov/geo/), and all accession numbers are given in Table S12. The data and scripts used were saved in GitHub (https://github.com/liulei3566/Pig_pan_genome). Supplementary materials (figures, tables, graphical abstracts, slides, videos, Chinese translated versions, and updated materials) may be found in the online DOI or iMeta Science http://www.imeta.science/.
