# Supplementary material for: Multiomics analysis reveals signatures of selection and loci associated with complex traits in pigs
Source: Imeta. 2024 Dec 15;3(6):e250. doi: 10.1002/imt2.250 (PMC12625778; doi:10.1002/imt2.250)
Supplement: Supplementary file 1 — Figure S1. The comprehensive analysis pipeline used in this study. Figure S2. Snail plots showing assembly statistics of six HiFi genomes. Figure S3. Dotplots showing patterns of synteny and collinearity between assembled genomes and Sscrofa11.1 reference. Figure S4. Genome‐wide structural variants. Figure S5. Global map of genetic variations in the pig genome. Figure S6. Examples of swept genes in EAD and WED pigs and functional enrichment results. Figure S7. Functional exploration of NR6A1 gene. Figure S8. Functional study of the BRCA1 variant. Figure S9. Distinct genomic landscape and functions of the coding variant in the ABCA3 gene. Figure S10. Global transcriptome profiles across skeletal muscle development in Landrace (LDR) and Tongcheng (TC) pigs. Figure S11. Integrative analysis of transcriptome data from Landrace (LDR) and Tongcheng (TC) pigs. Figure S12. Gene expression modules affected by selective sweeps. Figure S13. Comparison of skeletal muscle development in Landrace (LDR) and Tongcheng (TC) pigs. Figure S14. Significant differences in the expression levels between all expressed genes and the swept genes in EAD and WED groups. Figure S15. Distribution of DNA methylation profiles across the 27 skeletal muscle developmental stages in Landrace (LDR) and Tongcheng (TC) pigs. Figure S16. Comparisons of DNA methylation levels between Landrace (LDR) and Tongcheng (TC) pigs. Figure S17. Correlation between gene expression and DNA methylation levels of promoter regions in Landrace pig. Figure S18. Correlation between gene expression and DNA methylation levels of gene body regions in Landrace pig. Figure S19. Correlation between gene expression and DNA methylation levels of promoter regions in Tongcheng pig. Figure S20. Correlation between gene expression and DNA methylation levels of gene body regions in Tongcheng pig. Figure S21. Changed correlation patterns between gene expression and DNA methylation levels by selective sweeps. Figure S22. Comparison of the [file IMT2-3-e250-s001.docx]

**Supporting information to**

**Multiomics analysis reveals signatures of selection and loci associated with complex traits in pigs**

**Running title:** Genetic basis of complex traits in pigs

Lei Liu^1,2,3#^, Guoqiang Yi^1,2^^,4#*^, Yilong Yao^1,2#^, Yuwen Liu^1,2,4#^, Jiang Li^5#^, Yalan Yang^1,2^, Mei Liu^6^, Lingzhao Fang^7^, Delin Mo^8^, Longchao Zhang^9^, Yonggang Liu^10^, Yongchao Niu^5^, Liyuan Wang^1^, Xiaolu Qu^1^, Zhangyuan Pan^9^, Lei Wang^1^, Muya Chen^1^, Xinhao Fan^1,2^, Yun Chen^1^, Yongsheng Zhang^1^, Xingzheng Li^1^, Zhen Wang^1^, Yijie Tang^1^, Hetian Huang^11^, Pengxiang Yuan^1^, Yuying Liao^12^, Xinjian Li^11^, Zongjun Yin^13^, Di Liu^14^, Dongjie Zhang^14^, Quanyong Zhou^15^, Wangjun Wu^16^, Jicai Jiang^17^, Yahui Gao^18^, George E. Liu^18^, Lixian Wang^9^, Yaosheng Chen^8^, Kui Li^1^, Martien A M Groenen^19*^, Zhonglin Tang^1,2,4*^

^1^Shenzhen Branch, Guangdong Laboratory of Lingnan Modern Agriculture, Key Laboratory of Livestock and Poultry Multi-omics of MARA, Agricultural Genomics Institute at Shenzhen, Chinese Academy of Agricultural Sciences, Shenzhen 518124, China.

^2^Kunpeng Institute of Modern Agriculture at Foshan, Agricultural Genomics Institute, Chinese Academy of Agricultural Sciences, Foshan 528226, China.

^3^Yazhouwan National Laboratory, Sanya 572024, China.

^4^Guangxi Engineering Centre for Resource Development of Bama Xiang Pig, Bama 547500, China.

^5^Biozeron Shenzhen, Inc., Shenzhen 518081, China.

^6^College of Animal Science and Technology, Hunan Agricultural University, Changsha 410128, China.

^7^Center for Quantitative Genetics and Genomics, Aarhus University, Aarhus 8000, Denmark.

^8^State Key Laboratory of Biocontrol, School of Life Sciences, Sun Yat-sen University, Guangzhou 510006, China.

^9^Institute of Animal Sciences, Chinese Academy of Agricultural Sciences, Beijing 100193,

China.

^10^Faculty of Animal Science and Technology, Yunnan Agricultural University, Kunming 650201, China.

^11^College of Animal Sciences and Technology, Henan Agricultural University, Zhengzhou 450046, China.

^12^Guangxi Veterinary Research Institute, Nanning 530001, China.

^13^College of Animal Science and Technology, Anhui Agricultural University, Hefei 230036, China.

^14^Institute of Animal Husbandry, Heilongjiang Academy of Agricultural Sciences, Haerbin 150086, China.

^15^Institute of Animal Husbandry and Veterinary Medicine, Jiangxi Academy of Agricultural Sciences, Nanchang 330200, China.

^16^Department of Animal Genetics, Breeding and Reproduction, College of Animal Science and Technology, Nanjing Agricultural University, Nanjing 210095, China.

^17^Department of Animal Science, North Carolina State University, Raleigh, NC 27695, USA.

^18^Animal Genomics and Improvement Laboratory, Henry A. Wallace Beltsville Agricultural Research Center, Agricultural Research Service, USDA, Beltsville, Maryland 20705, USA.

^19^Animal Breeding and Genomics Group, Wageningen University & Research, PO Box 338, 6700 AH, Wageningen, The Netherlands.

^#^These authors contributed equally to this work: Lei Liu, Guoqiang Yi, Yilong Yao, Yuwen Liu, Jiang Li

^*^Correspondence: [tangzhonglin@caas.cn](mailto:tangzhonglin@caas.cn) (Zhonglin Tang), [martien.groenen@wur.nl](mailto:martien.groenen@wur.nl) (Martien A M Groenen), [yiguoqiang@caas.cn](mailto:yiguoqiang@caas.cn) (Guoqiang Yi)


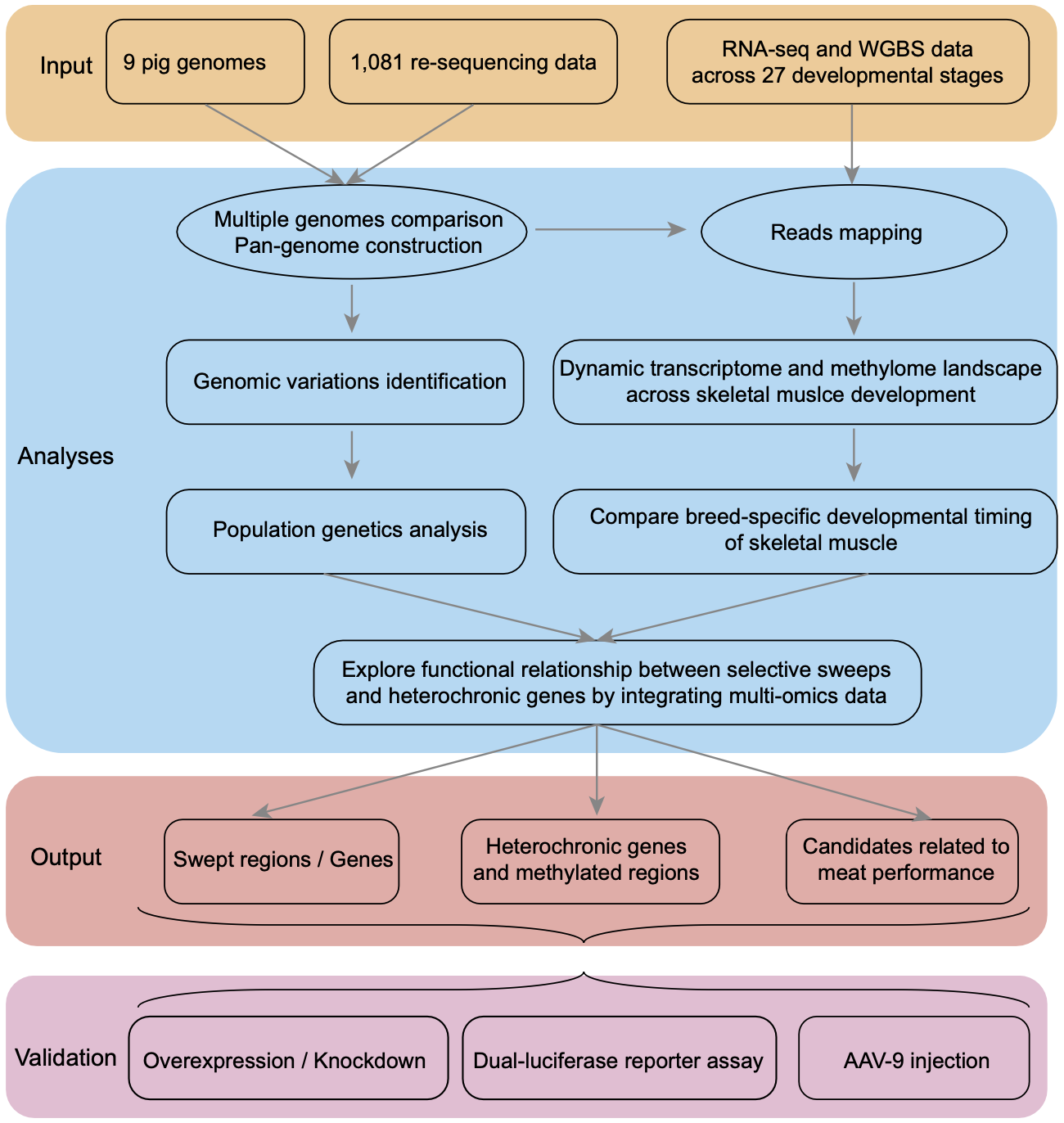


**Figure S1 The comprehensive analysis pipeline used in this work.**

**
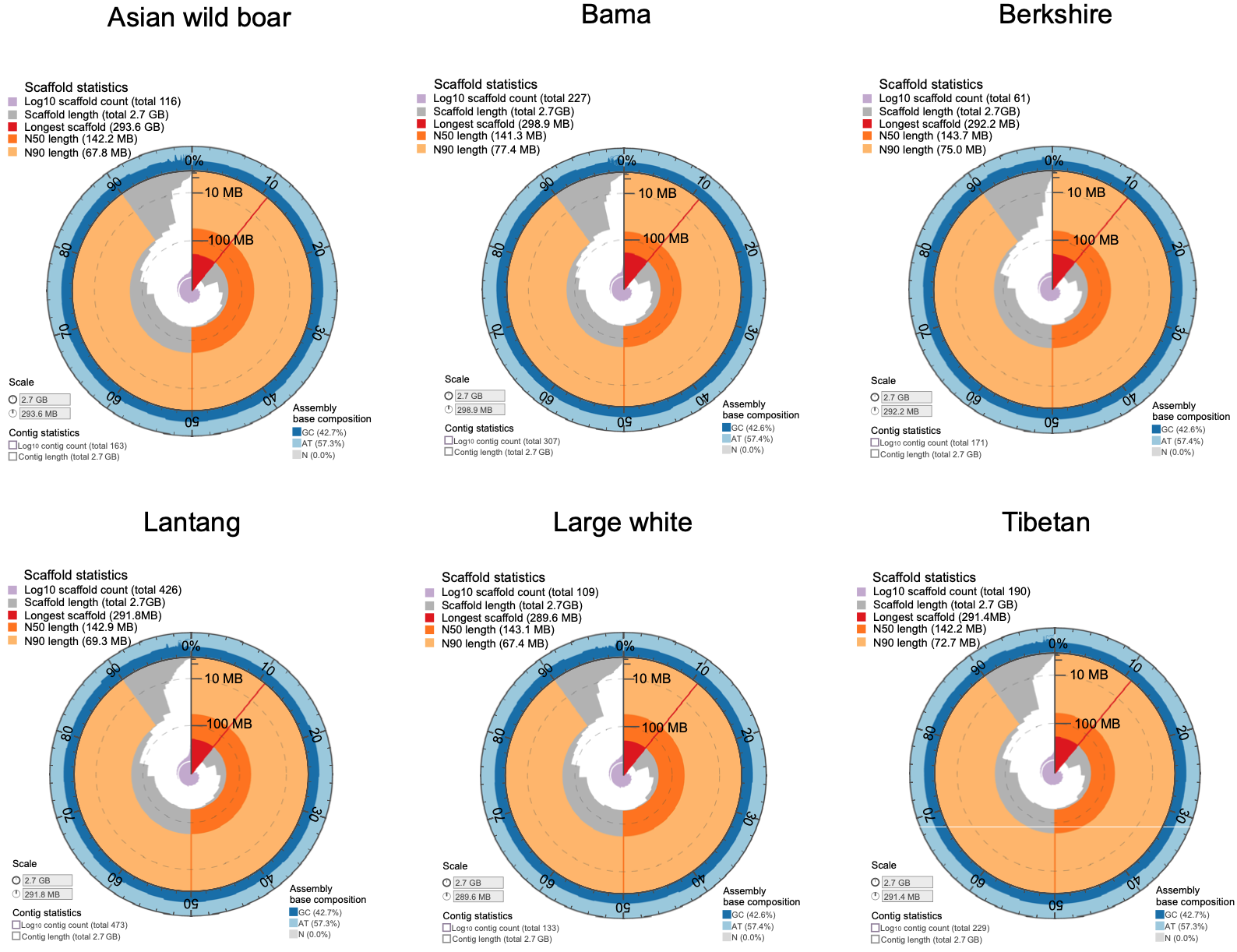
**

**Figure S2 Snail plots showing assembly statistics of six HiFi genomes.**

**
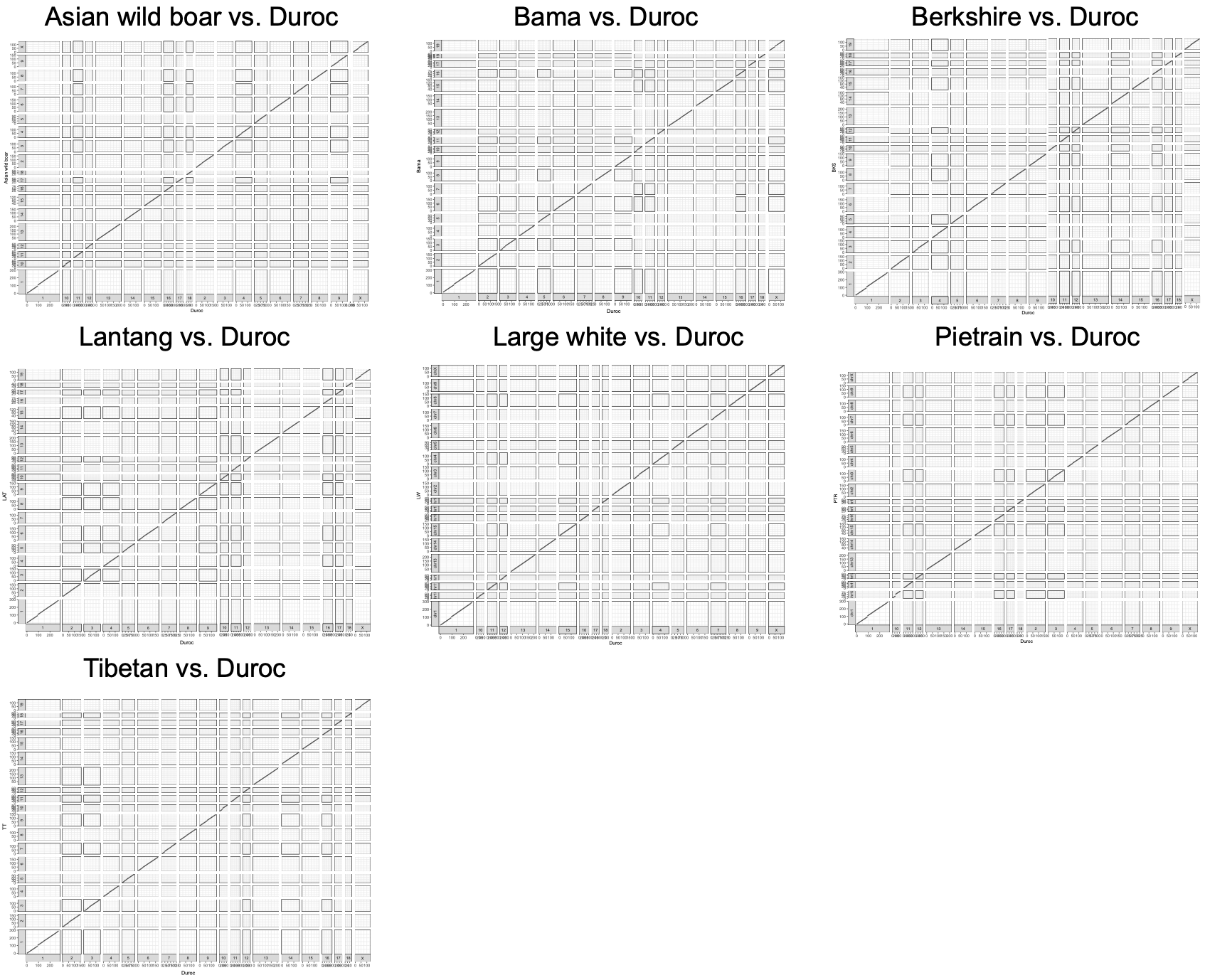
**

**Figure S3 Dotplots showing patterns of synteny and collinearity between assembled genomes and Sscrofa11.1 reference.**

**
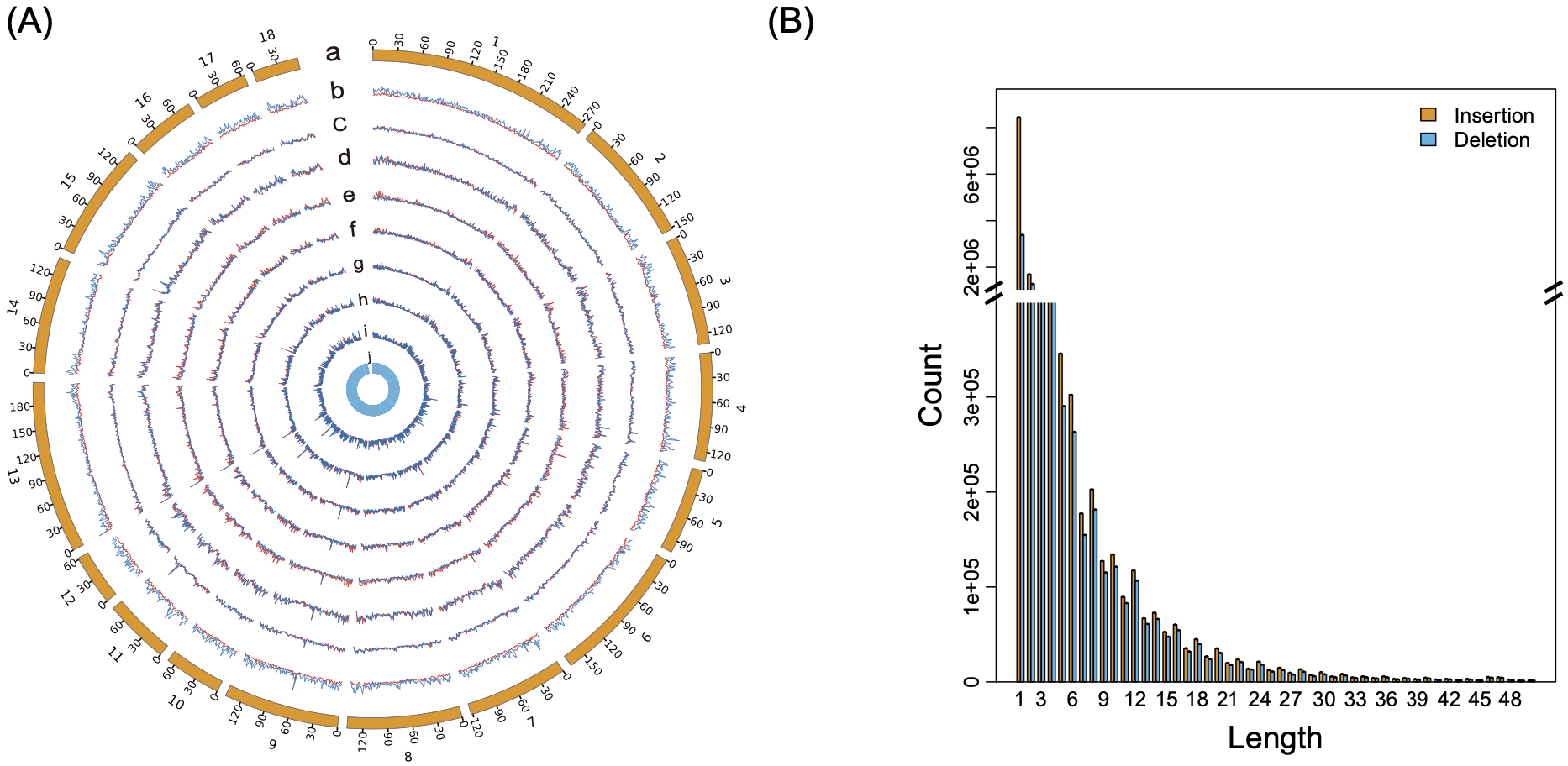
**

**Figure S4 Genome-wide structural variants.** (A) Circos plot of the presence-absence variations including SNPs (red) and SVs (blue) for each pig genome compared with Sscrofa11.1 reference. The innermost blue circle is gene density. The window size is 1Mb. (B) The length distribution of genomic variations shorter than 50 bp.

**
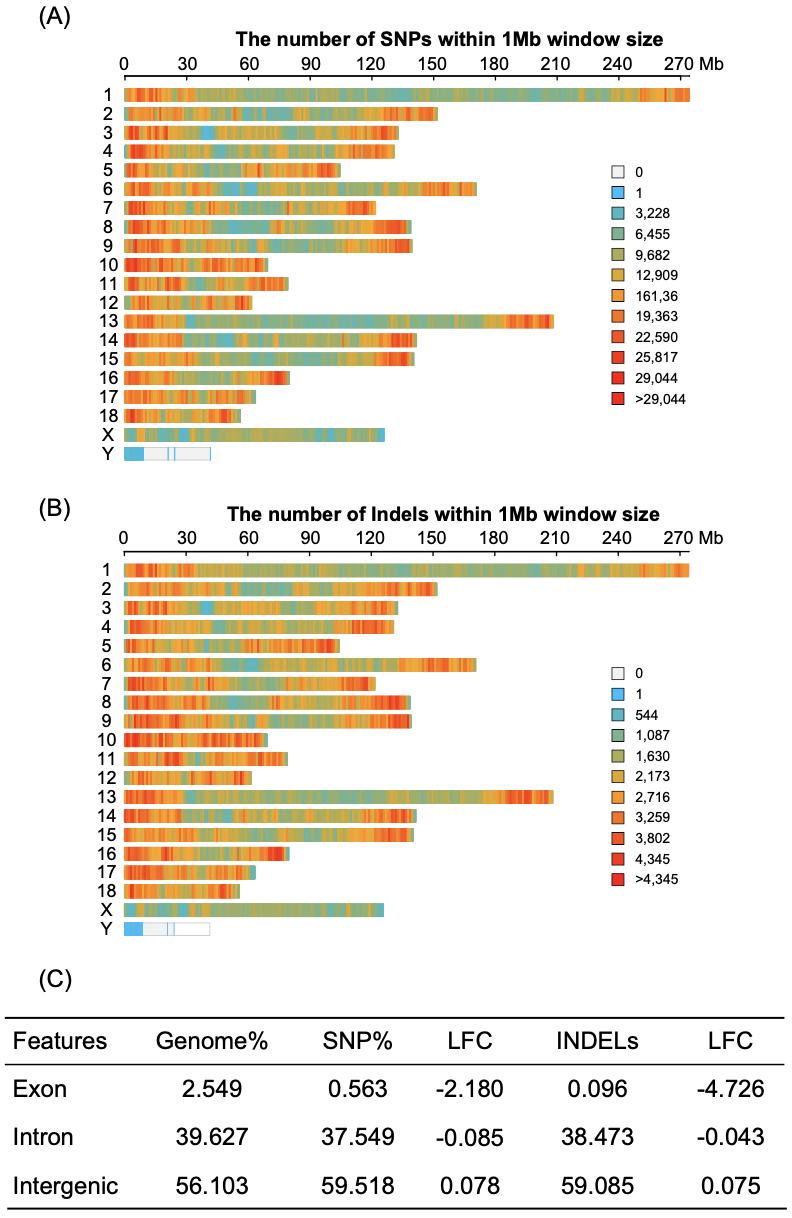
**

**Figure S5 Global map of genetic variations in the pig genome.** (A) Density map of SNPs within 1Mb window size. (B) Density map of INDELs within 1Mb window size. (C) Relative enrichment or depletion of SNPs and INDELs within exons, introns and intergenic regions. SNPs, single nucleotide polymorphisms. INDELs, insertions and deletions. LFC, log_2_fold change.


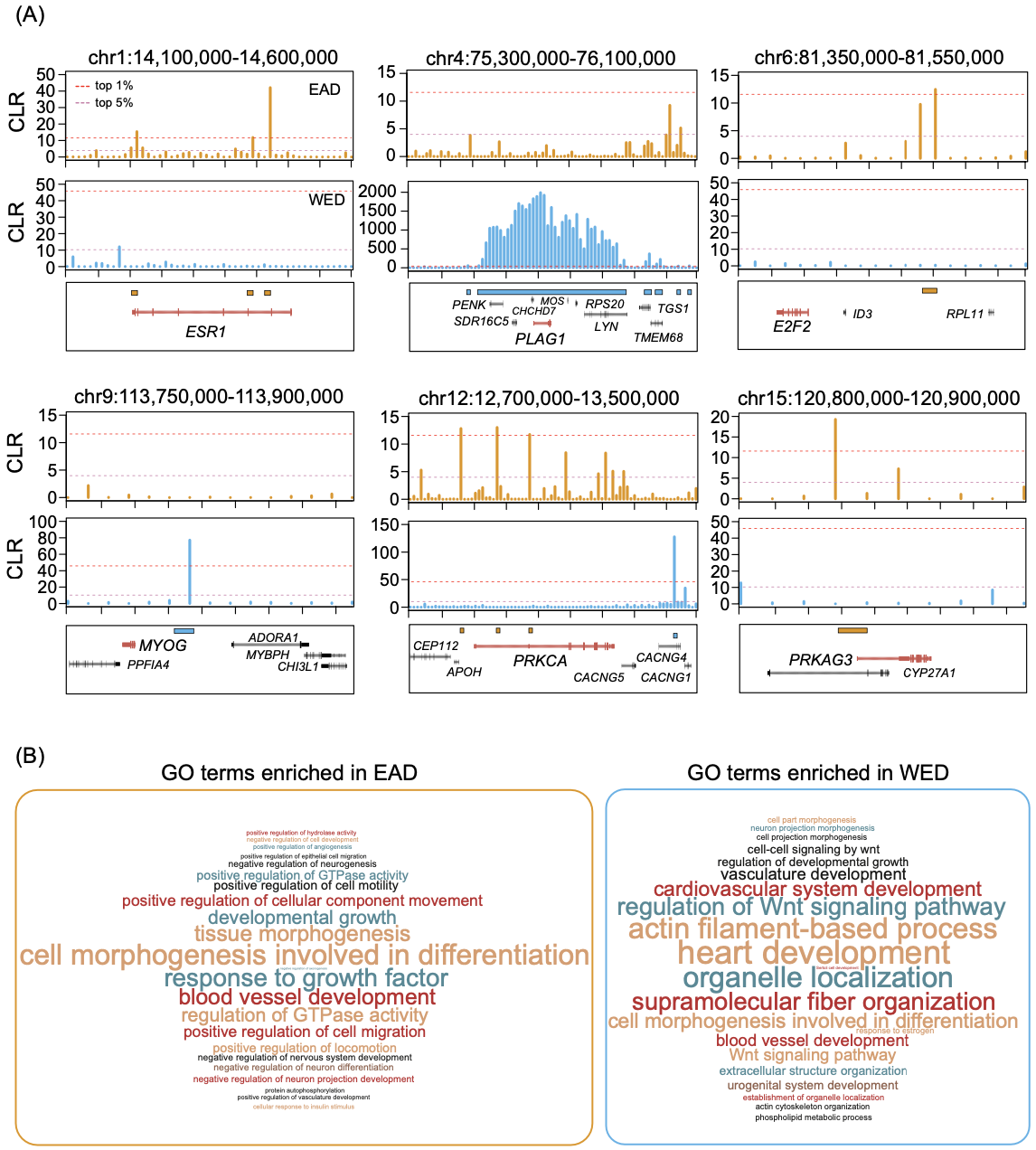


**Figure S6 Examples of swept genes in** **EAD and WED pigs and functional enrichment results.** (A) The composite likelihood ratio (CLR) values of several key genes in EAD and WED pigs. (B) Word cloud of significant biological process terms (*q*-value < 0.05) based on swept genes in EAD and WED populations. EAD, eastern domestic group. WED, western domestic group.


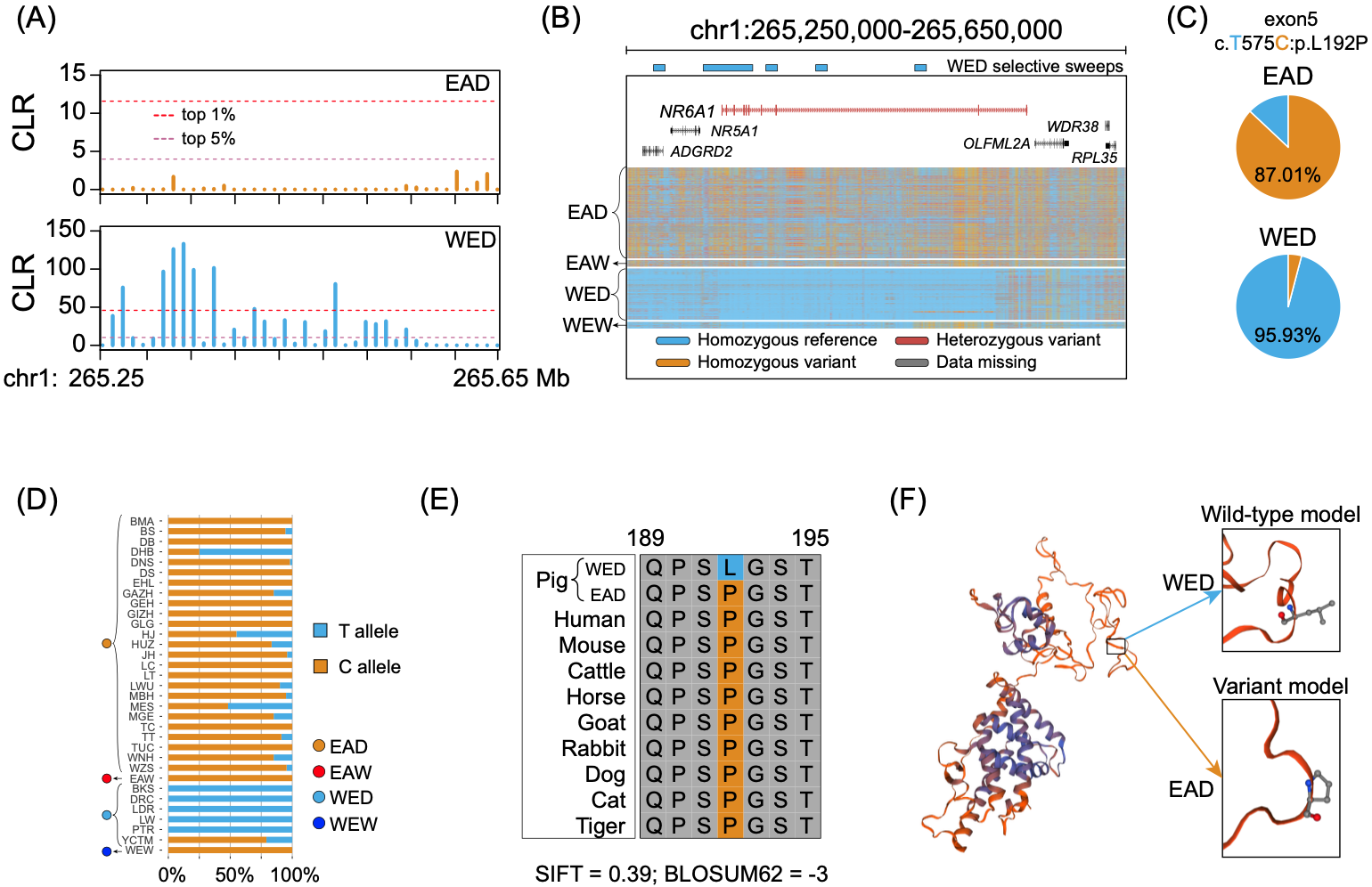


**Figure S7 Functional exploration of** ***NR6A1* gene.** (A) Comparison of CLR values in the *NR6A1* region between EAD and WED pigs. (B) Genotype spectrum around the *NR6A1* region among different pig populations. (C) Different allele frequencies of the coding mutation between EAD and WED breeds. (D) The allele frequency of this variant among different Eurasian pig breeds. BMA, Bama; BS, Baoshan; DB, Debao; DHB, Dahuabai; DNS, Diannan small ear; DS, Dongshan; EHL, Erhualian; GAZH, Guanzhuanghua; GEH, Guangdong litter ear hua; GIZH, Guizhonghua; GLG, Gaoligongshan; HJ, Huanjiang; HUZ, Huai; JH, Jinhua; LC, Luchuan; LT, Lantang; LWU, Laiwu black; MBH, Minbei hua; MES, Meishan; MGE, Mingguang little ear; TC, Tongcheng, TT, Tibetan; TUC, Tunchang; WNH, Wannan black; WZS, Wuzhishan; EAW, Eastern wild boar; BKS; Berkshire; DRC, Duroc; LDR, Landrace; LW, Large white; PTR, Pretriain; YCTM, Yucatan; WEW, Western wild boards. The number of pigs in each breed is greater than 10. (E) Multi-species alignment of NR6A1 protein for which domestic pigs are fixed or close to fixation for a derived amino acid substitution. This result indicates that Pro is more conserved than Leu. (F) Alteration in protein structure resulting from the mutation in the NR6A1 protein. This single amino acid substitution might exert an adverse effect in the protein folding.


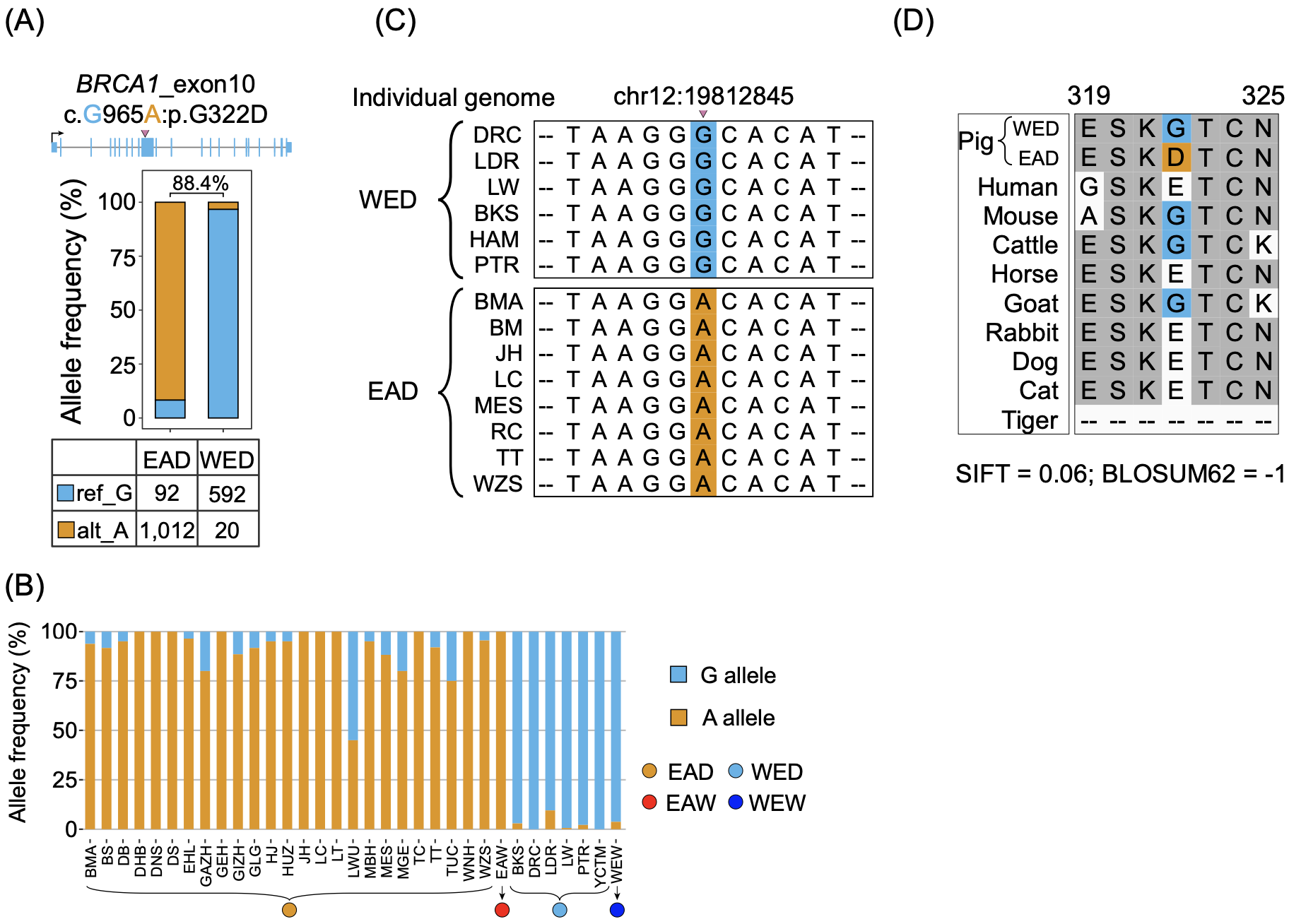


**Figure S8 Functional study of the *BRCA1* variant.** (A) The allele frequency of the coding mutation c.G965A in EAD and WED populations. (B) Comparisons of this variant among different breeds with a sample size greater than 10. The full name for each breed can be found in Table S12. (C) Comparisons of the sequences around this missense mutation across the 14 pig assemblies in pan-genome analysis. (D) Multiple sequence alignment of amino acids across ten representative mammalian species.


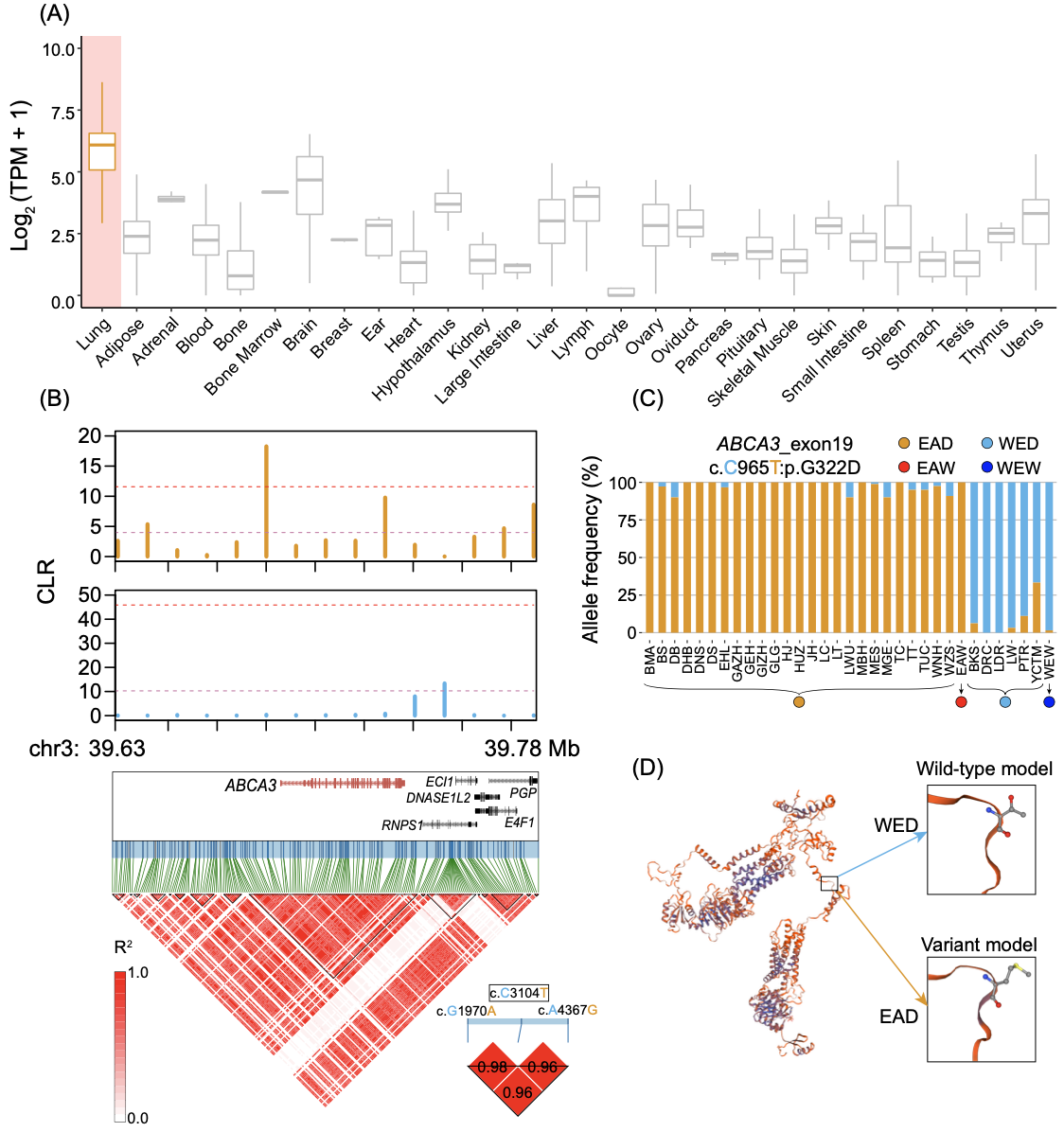


**Figure S9** **Distinct genomic landscape and functions of the coding variant in the *ABCA3* gene.** (A) The *ABCA3* gene shows predominant expression in lung tissues. (B) CLR comparison and LD pattern around the *ABCA3* region. (C) The allele frequency of the mutation c.C965T in *ABCA3* among different pig breeds. The full name for each breed can be found in Table S12. (D) Alteration in protein structure resulting from the mutation in ABCA3 protein.


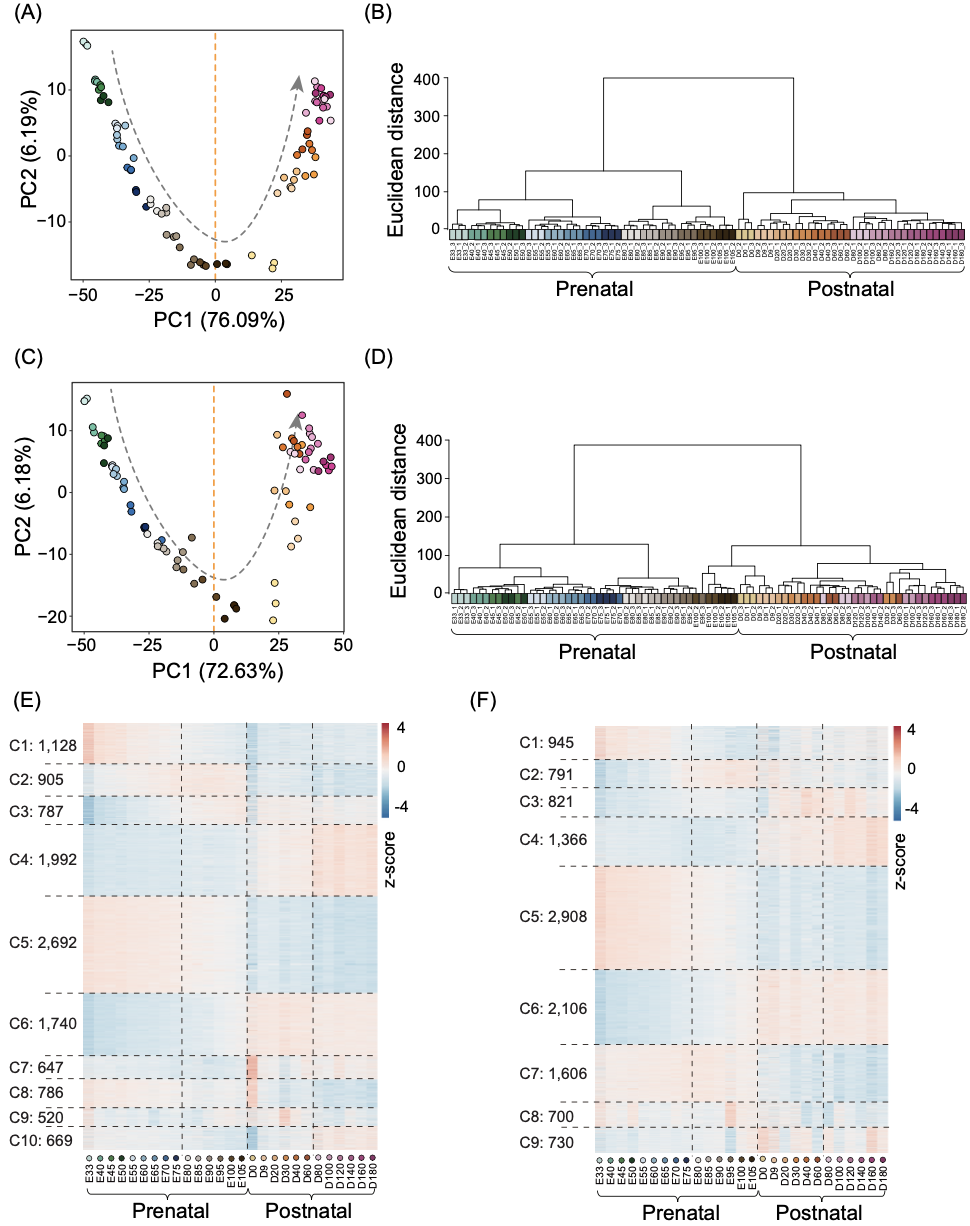


**Figure S10 Global transcriptome profiles across skeletal muscle development in Landrace (LDR) and Tongcheng (TC) pigs.** (A) PCA analysis of gene expression data according to top 1,500 variable genes in LDR. (B) Hierarchical clustering based on the expression levels of top 1,500 variable genes in LDR. Unsupervised PCA and hierarchical clustering results showed the excellent agreement among biological replicates, and a smooth transition from one stage to the neighbouring phase. (C) PCA analysis of gene expression data according to top 1,500 variable genes in TC. (D) Hierarchical clustering based on the expression levels of top 1,500 variable genes in TC. (E-F) Distinct expression patterns of those dynamic genes by k-means clustering among 27 developmental stages in LDR and TC pigs. By grouping differentially expressed genes (DEGs) with similar transcriptional patterns by k-means clustering approach, we defined nine and 10 clusters in TC and LDR, respectively.


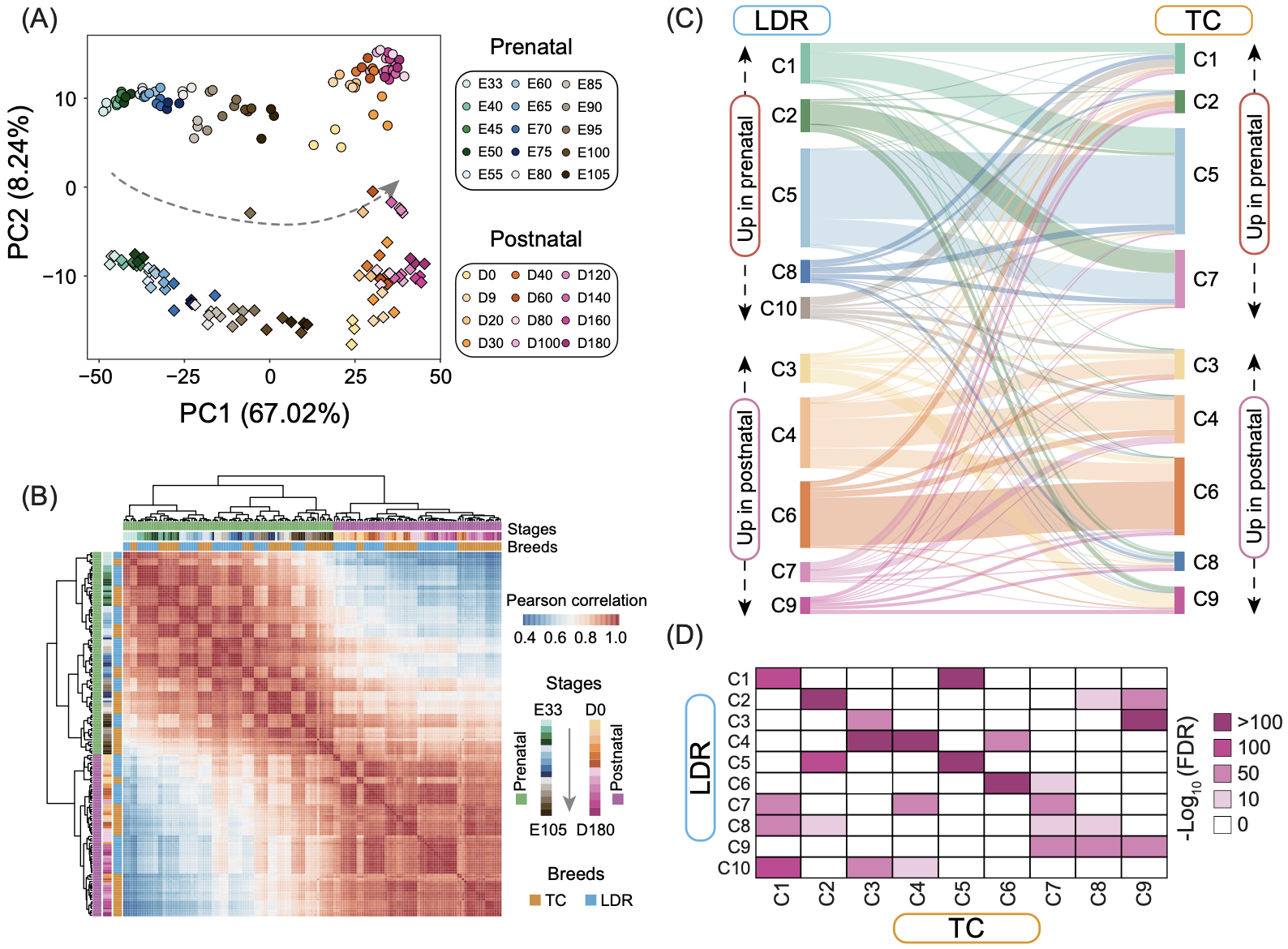


**Figure S11 Integrative analysis of transcriptome data from** **Landrace (LDR) and Tongcheng (TC) pigs.** (A) PCA analysis based on the expression levels of top 1,500 variable genes from all LDR and TC individuals. A combined analysis based on all RNA-seq data from two breeds. We found a clear separation along developmental trajectories by PC1 accounting for 67.02% of the observed variance, and also robust classification of breeds by PC2 despite that this component explained only 8.24% of the variation. The circular and diamond-shaped points represent LDR and TC samples, respectively. (B) Pearson correlation plots of all LDR and TC individuals based on gene expression. (C) Sankey plot shows the similarity of different clusters separately defined in LDR and TC pigs. The difference between the clusters of LDR and TC may be caused by differentially progressing genes (DPGs). (D) Hypergeometric test among different clusters from LDR and TC pigs. Our results confirmed statistically significant concordance between TC and LDR, suggesting that the majority of genes coordinated myogenesis in a similar manner regardless of breeds.


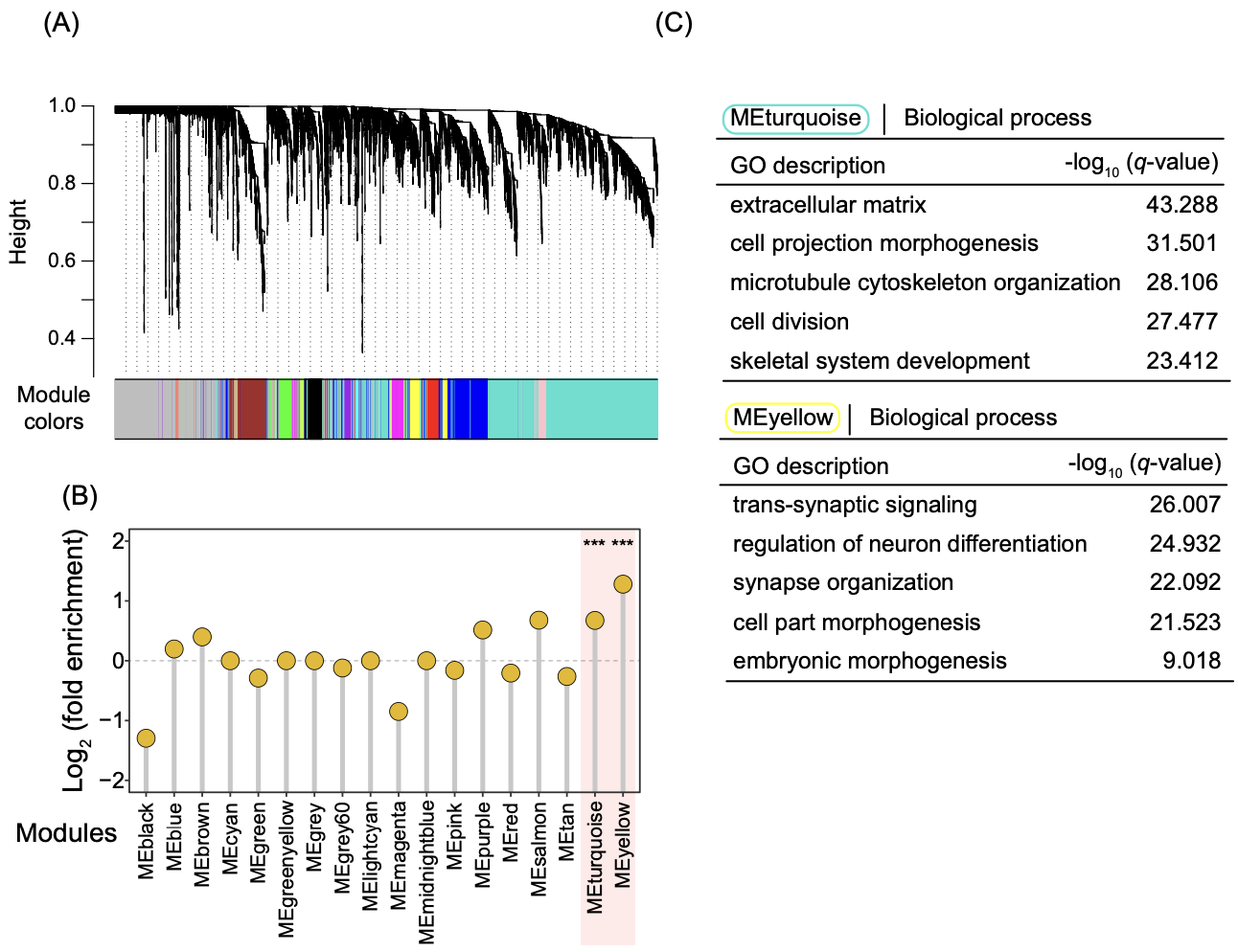


**Figure S12 Gene expression modules affected by selective sweeps.** (A) Eighteen different modules derived by weighted gene co-expression network analysis (WGCNA) based on all LDR and TC individuals. (B) Fold enrichment of different modules with putative swept genes. (C) Enriched biological processes with the genes in MEturquoise and MEyellow modules. The *p* value has been corrected for multiple testing by FDR. ^*^*p* < 0.05; ^**^*p* < 0.01; ^***^*p* < 0.001; ns, not significant.

**
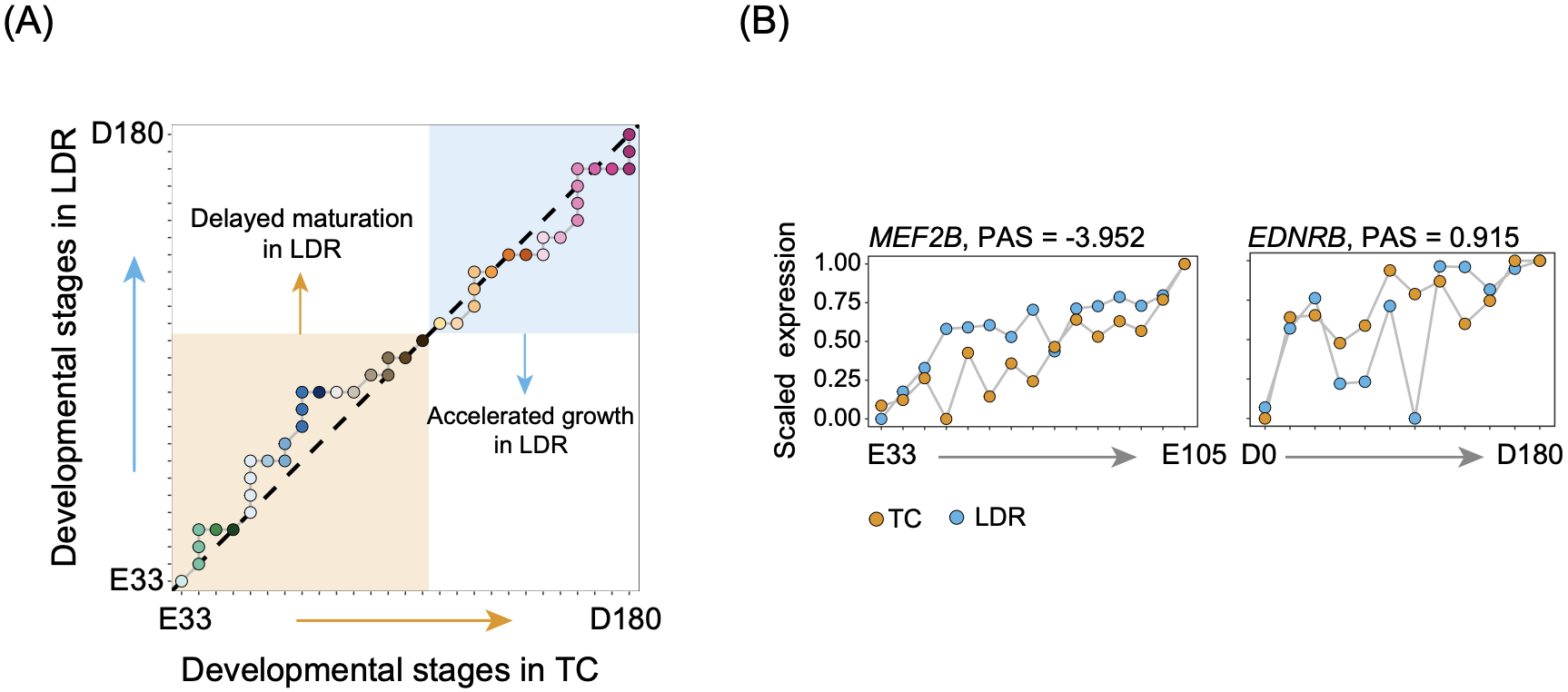
**

**Figure S13 Comparison of skeletal muscle development in Landrace (LDR) and Tongcheng (TC) pigs.** (A) Developmental stage correspondences between LDR and TC pigs during myogenesis. (B) Examples of developmental heterochrony for *MEF2B* and *EDNRB* genes. PAS, progression advanced score.


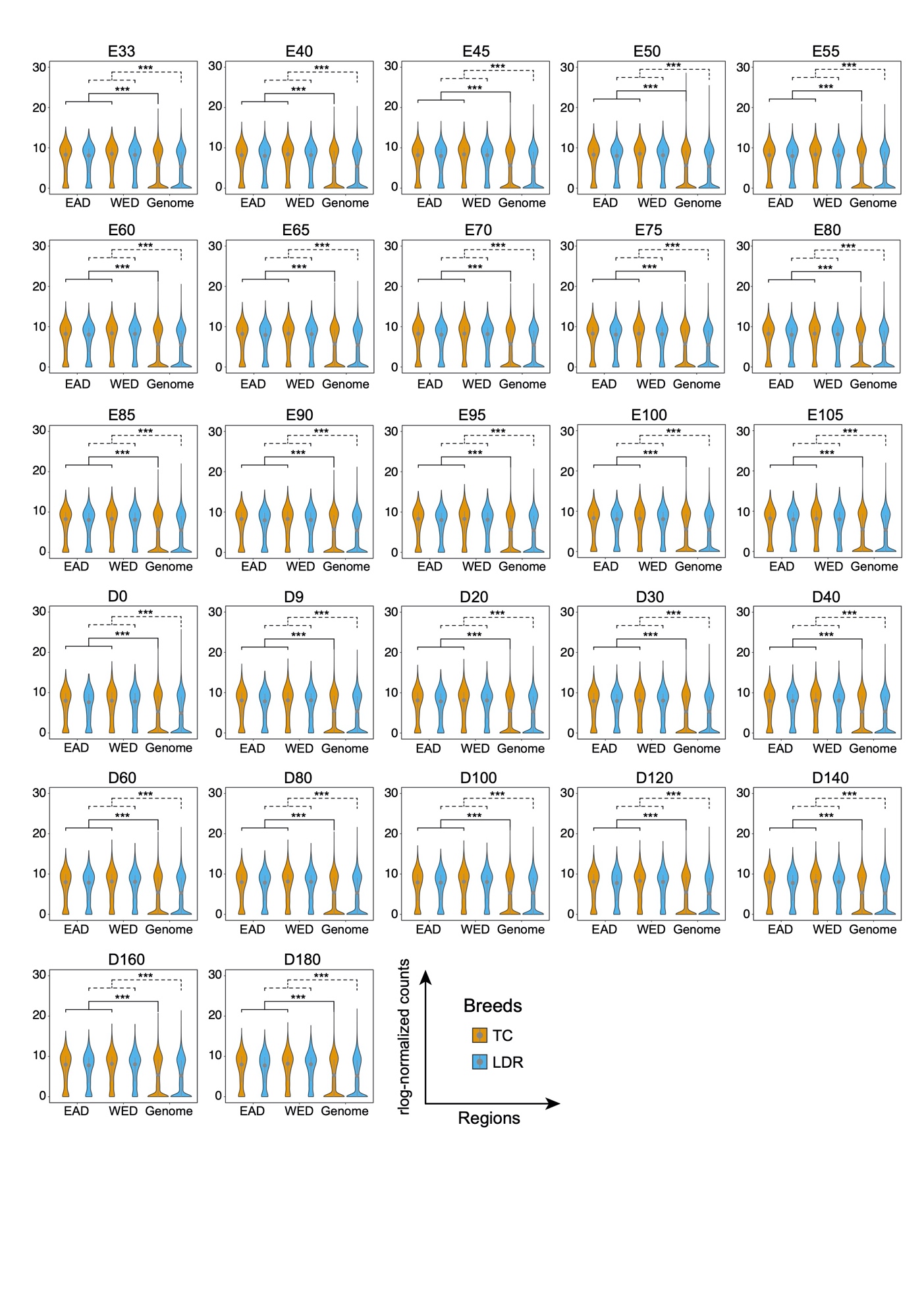


**Figure S14** **Significant differences in the expression levels between all expressed genes and the swept genes in EAD and WED groups**. ^***^*p* < 0.001.


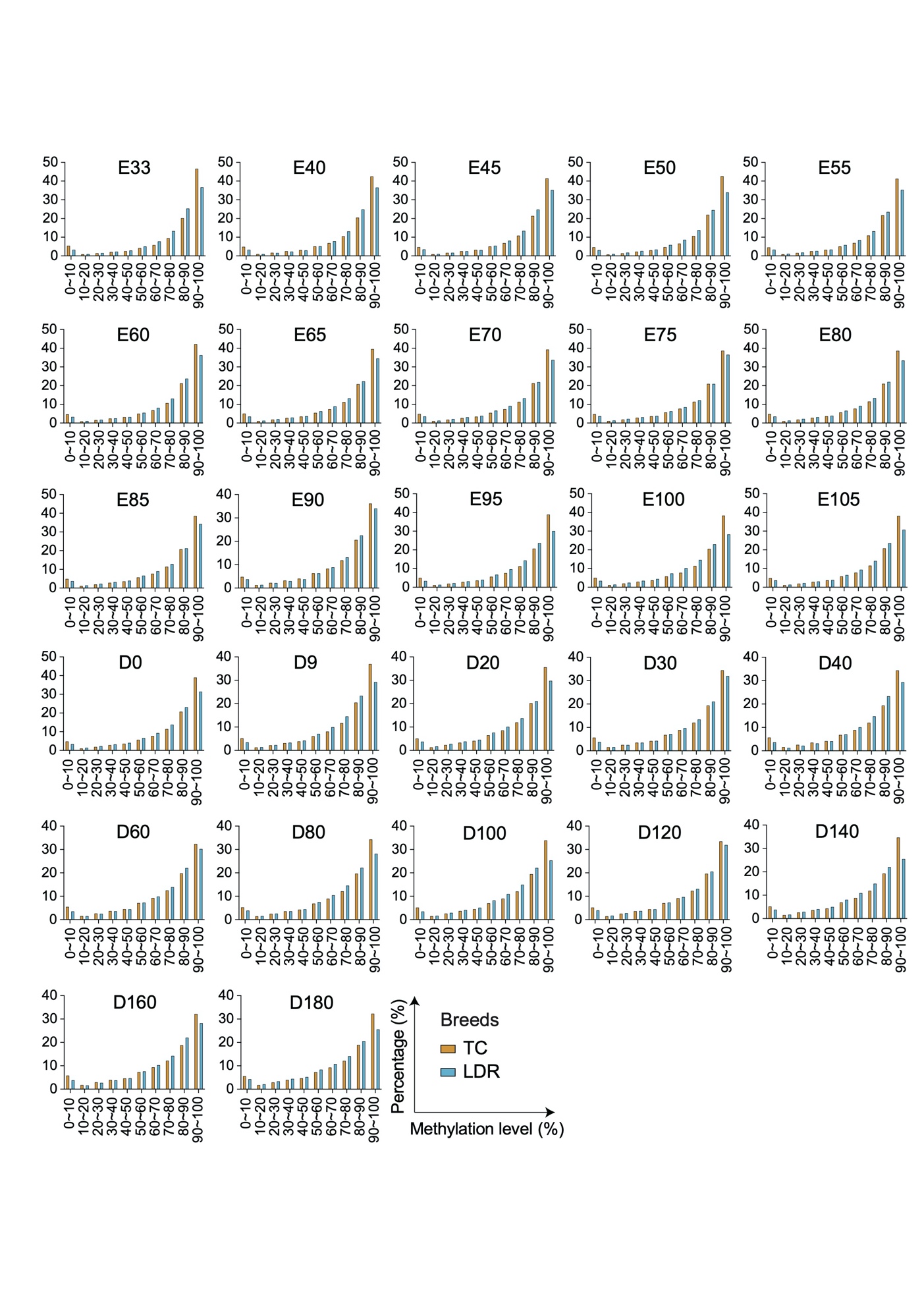


**Figure S15 Distribution of DNA methylation profiles across the 27 skeletal muscle developmental stages in Landrace (LDR) and Tongcheng (TC) pigs.** The DNA methylation levels are divided evenly into ten groups. We found an average of 72.61% and 71.63% methylation over all CpG sites in TC and LDR genomes respectively, and the majority of sites at each developmental stage showed very high methylation states of greater than 80%.

**
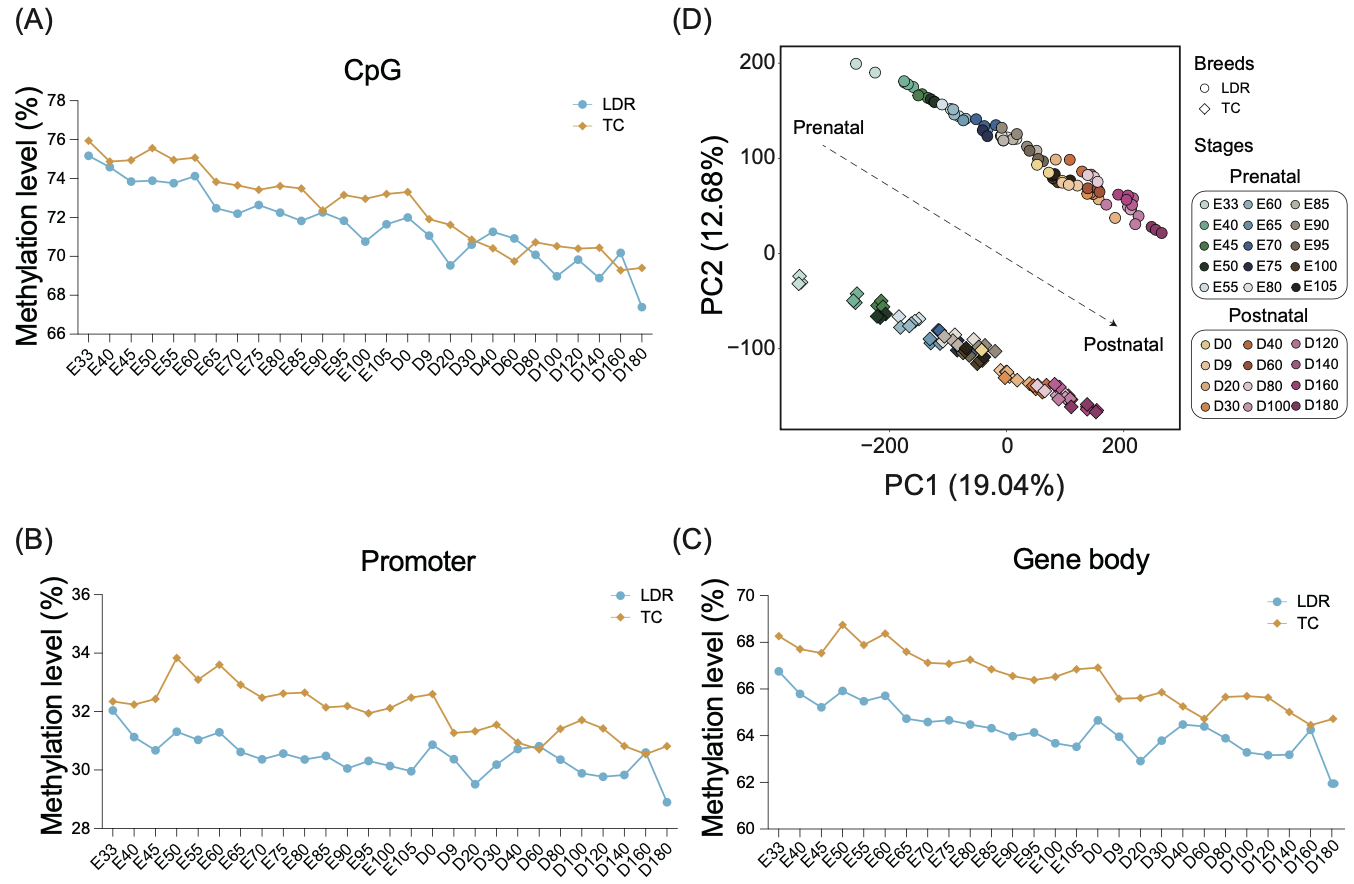
**

**Figure S16 Comparisons of DNA methylation levels between** **Landrace (LDR) and Tongcheng (TC) pigs.** (A-C) Dynamic DNA methylation landscape for al CpG sites (A), gene body (B) and promoter regions (C) in LDR and TC breeds. The gradual decrease of methylation levels in all CpG sites were detected throughout the whole development, with the biggest difference close to 10% from prenatal day 33 (E33) to postnatal day 180 (D180), which were similar between TC and LDR. Notably, the TC breed exhibited much heavier CpG methylation levels than LDR across the whole genome in almost all 27 developmental stages, and the same results were found in the promoters and gene bodies. (D) PCA analysis based on the DNA methylation levels of LDR and TC pigs.


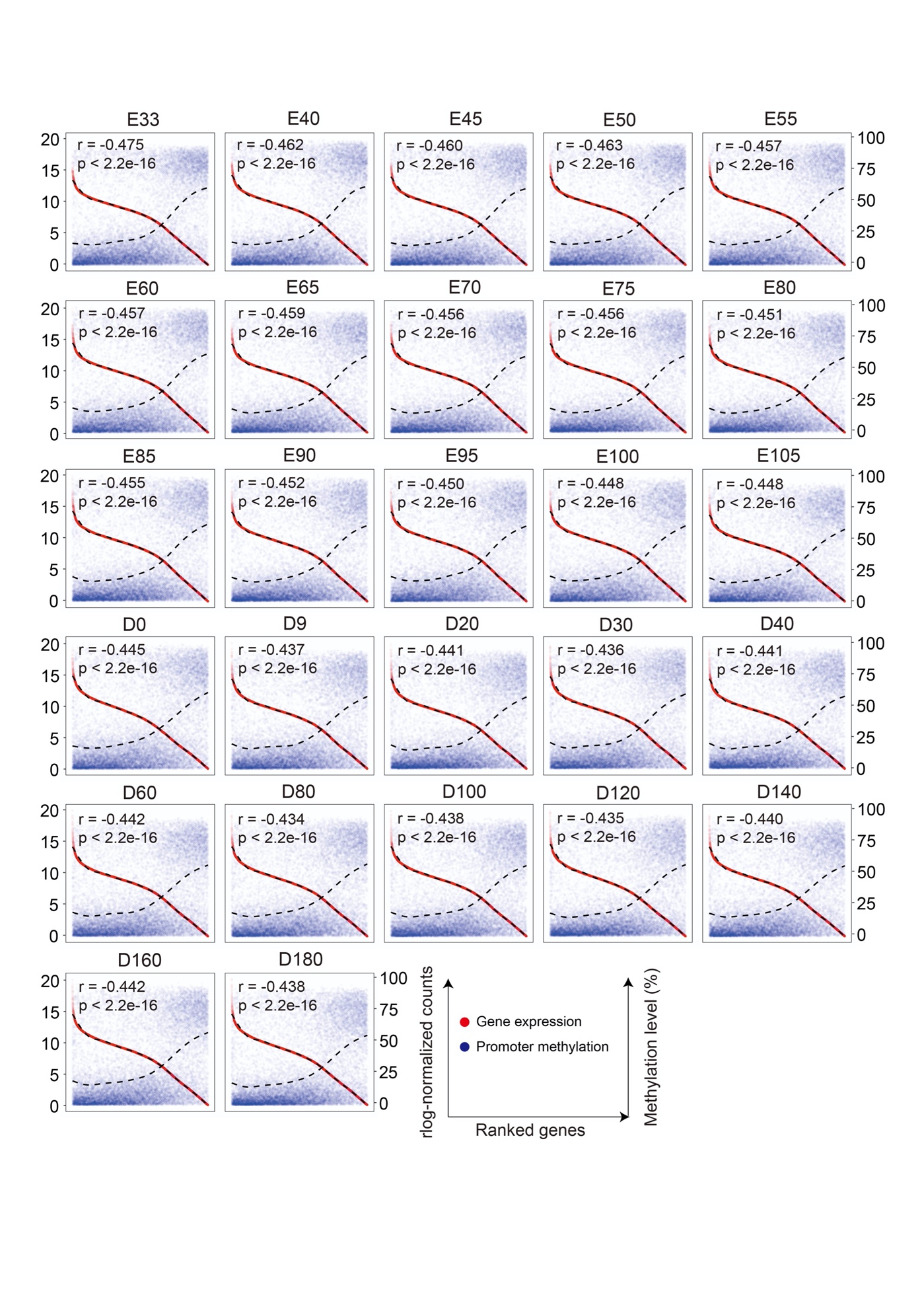


**Figure S17 Correlation between** **gene expression and DNA methylation levels of promoter regions in Landrace pig.** The Pearson correlation coefficients (*r*) between gene expression levels and DNA methylation levels were calculated. The dotted fitting curves separately represent gene expression levels and DNA methylation levels. The horizontal axis from left to right below each box represents the expression gene levels from high to low. The promoter-associated CpG islands have profound suppression effects in transcriptional levels with negative correlation coefficients close to -0.500.


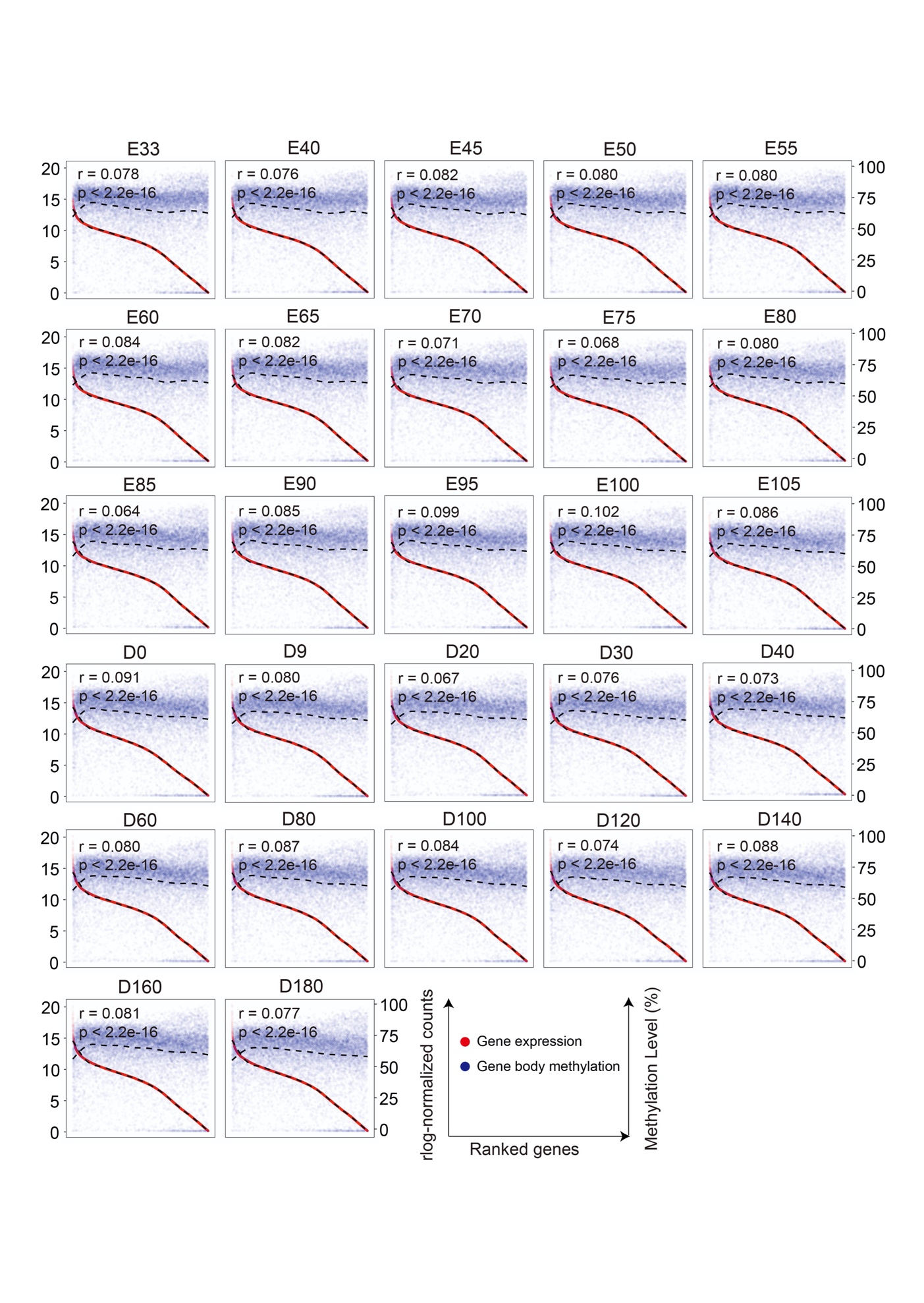


**Figure S18** **Correlation between gene expression and DNA methylation levels of gene body regions in Landrace pig.** The Pearson correlation coefficients (*r*) between gene expression levels and DNA methylation levels were calculated. The dotted fitting curves separately represent gene expression levels and DNA methylation levels. The horizontal axis from left to right below each box represents the gene expression levels from high to low. Only weak correlations between DNA methylation and gene expression were found across the gene body regions, suggesting a more complex regulatory manner of gene body DNA methylation.


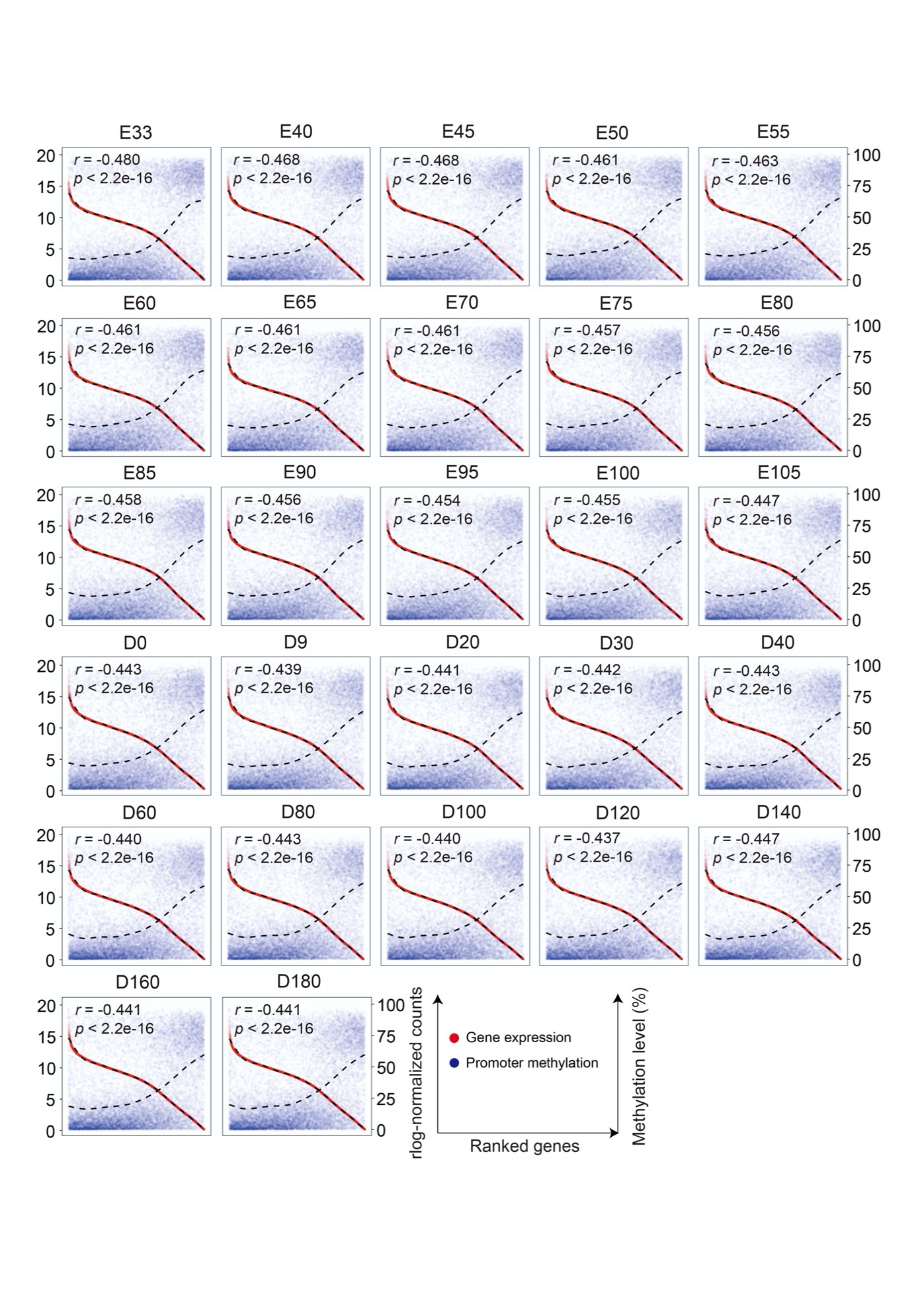


**Figure S19 Correlation between gene expression and DNA methylation levels of promoter regions in Tongcheng pig.** The Pearson correlation coefficients (*r*) between gene expression levels and DNA methylation levels were calculated. The dotted fitting curves separately represent gene expression levels and DNA methylation levels. The horizontal axis from left to right below each box represents the gene expression levels from high to low.


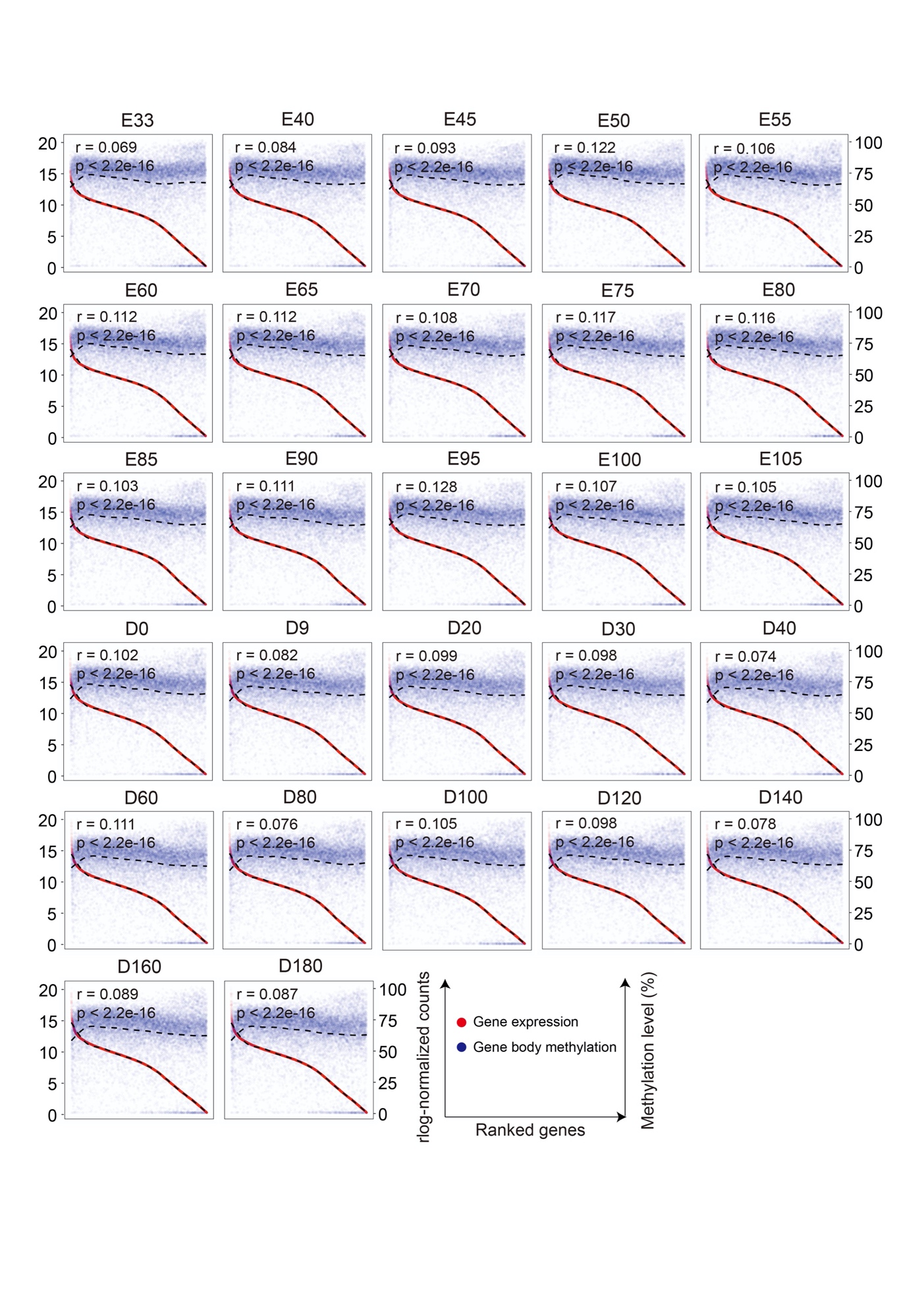


**Figure S20 Correlation between gene expression and DNA methylation levels of gene body regions in Tongcheng pig.** The Pearson correlation coefficients (*r*) between gene expression levels and DNA methylation levels were calculated. The dotted fitting curves separately represent gene expression level and DNA methylation level. The horizontal axis from left to right below each box represents the gene expression levels from high to low.


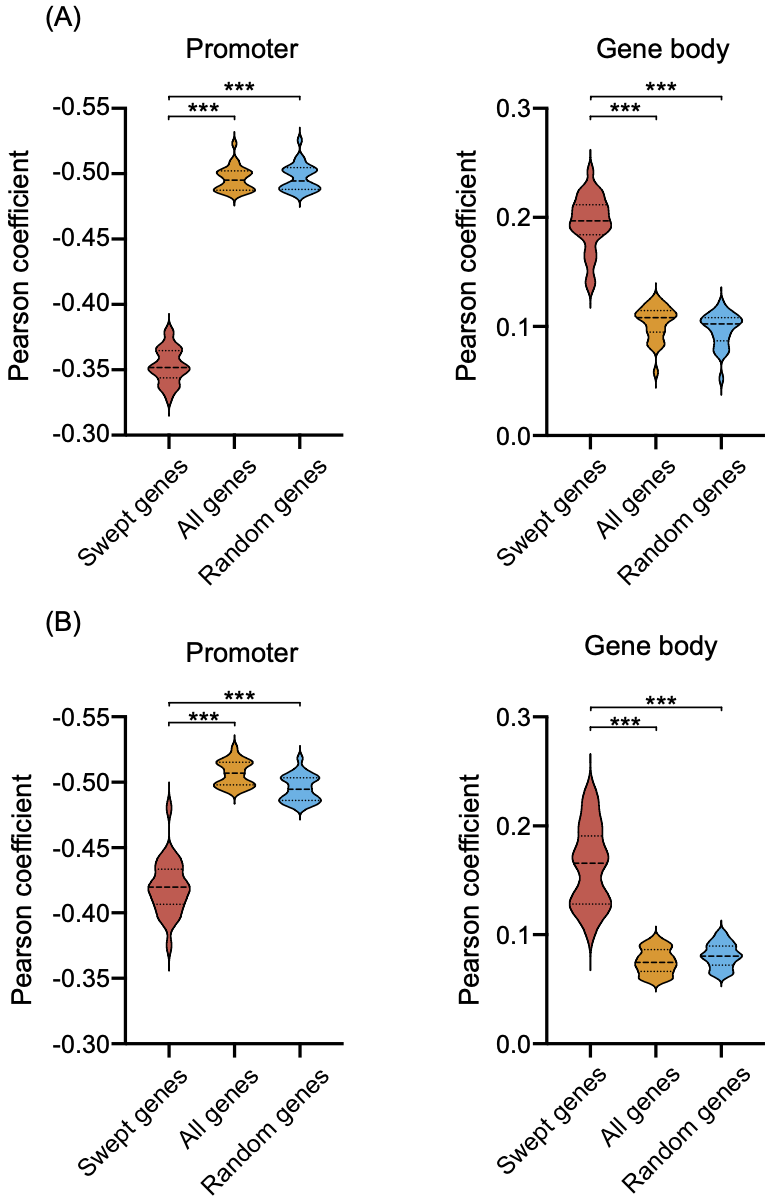


**Figure S21** **Changed correlation patterns between gene expression and DNA methylation levels by selective sweeps.** (A-B) Correlation coefficients of swept genes are lower for promoter regions, but higher for gene body regions in both TC (A) and LDR (B) pigs, compared with these of all genes and randomly selected genes. The number of random genes was matched to swept genes in EAD and WED groups, and the Pearson correlation coefficients are calculated based on 1,000 permutations. ^***^*p* < 0.001.


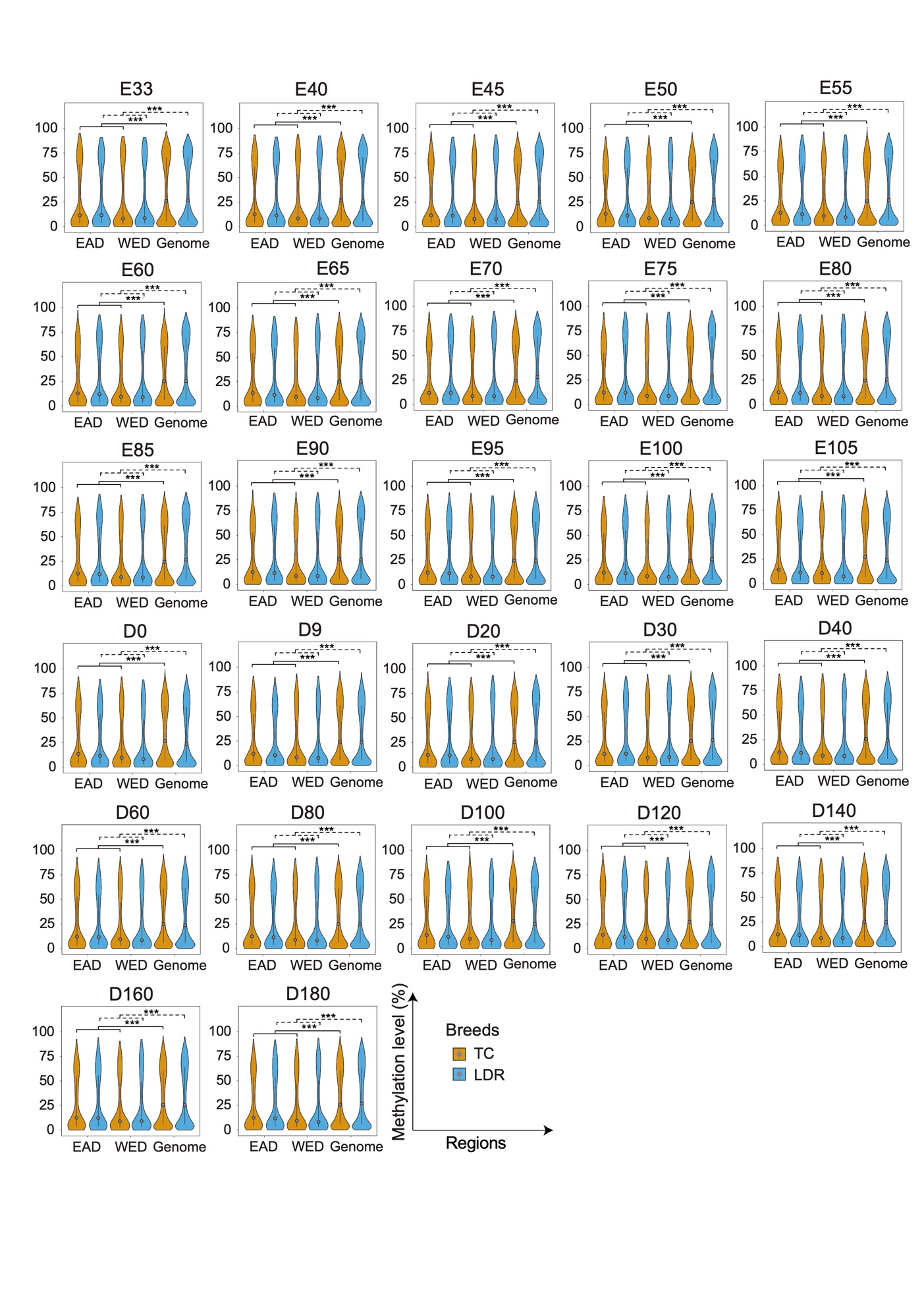


**Figure S22 Comparison of the DNA methylation levels in promoter regions for all genes and swept genes.** ^***^*p* < 0.001.

**
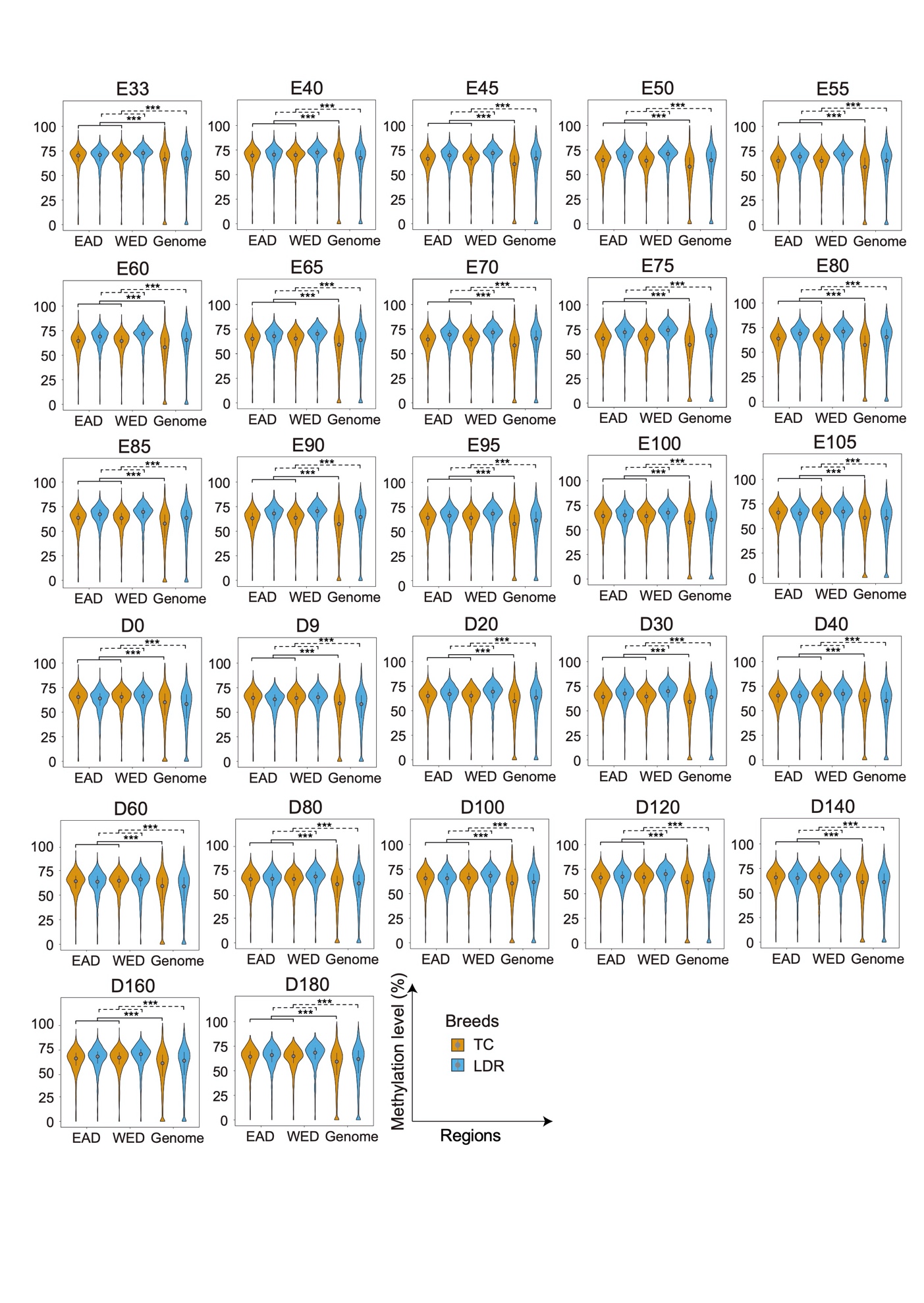
**

**Figure S23 Comparison of the DNA methylation levels in gene body regions for all genes and swept genes.** ^***^*p* < 0.001.**
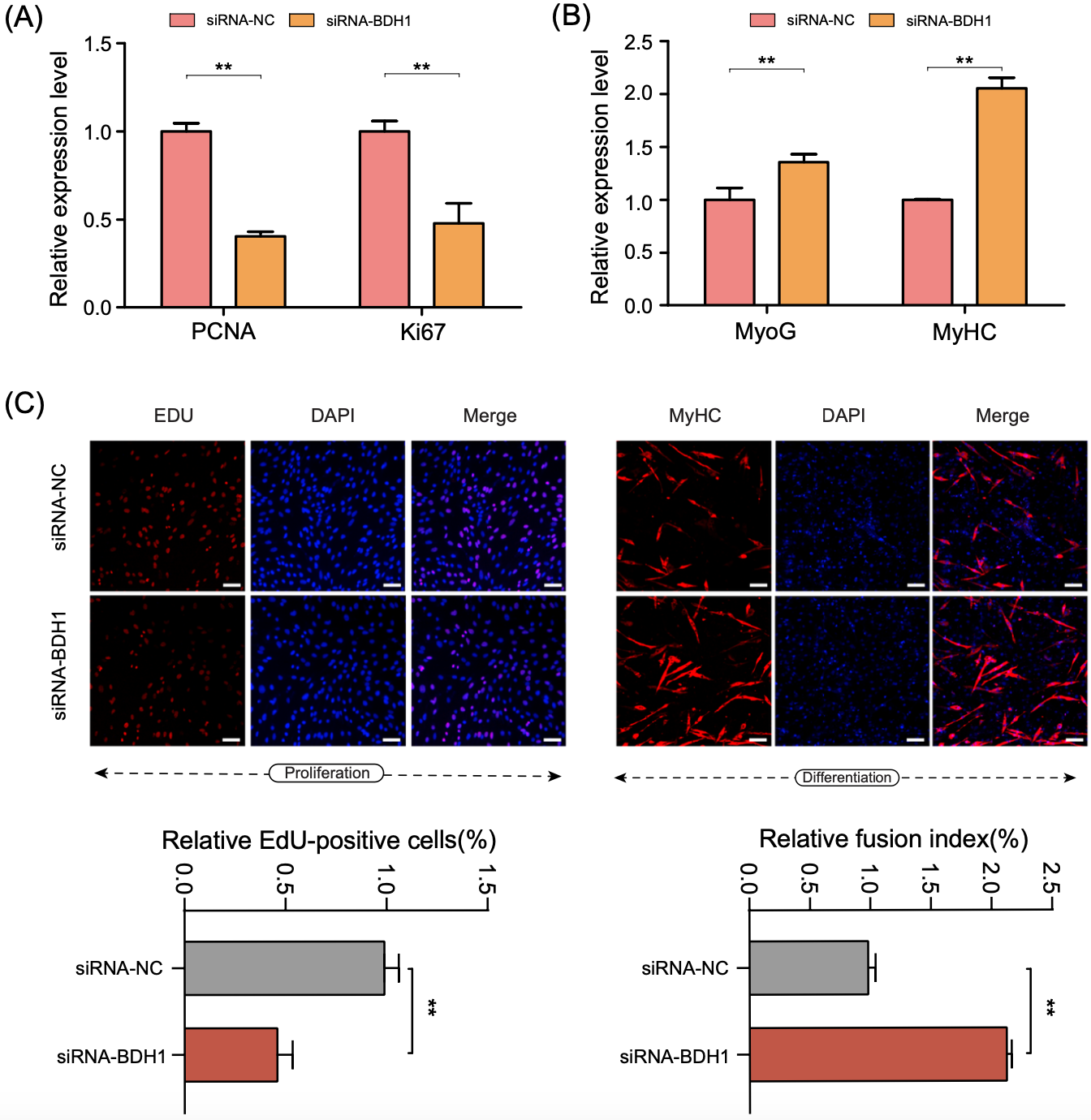
**

**Figure S24 Cell differentiation assessment for the expression levels of *PCNA*, *Ki67*, *MyoG*, and *MyHC* marker upon *BDH1* knockdown by qRT-PCR analysis in C2C12 cells.** (A) Cell proliferation assessment for the expression level of *PCNA* marker upon *BDH1* knockdown by qRT-PCR analysis in C2C12 cells. (B) Cell differentiation assessment for the expression levels of *MyHC* and *MYOD* marker upon *BDH1* knockdown by qRT-PCR analysis in C2C12 cells. (C) Cell proliferation and differentiation experiments measured by EDU and MyHC immunofluorescence upon *BDH1* knockdown. ^*^*p* < 0.05; ^**^*p* < 0.01; ^***^*p* < 0.001; ns, not significant.


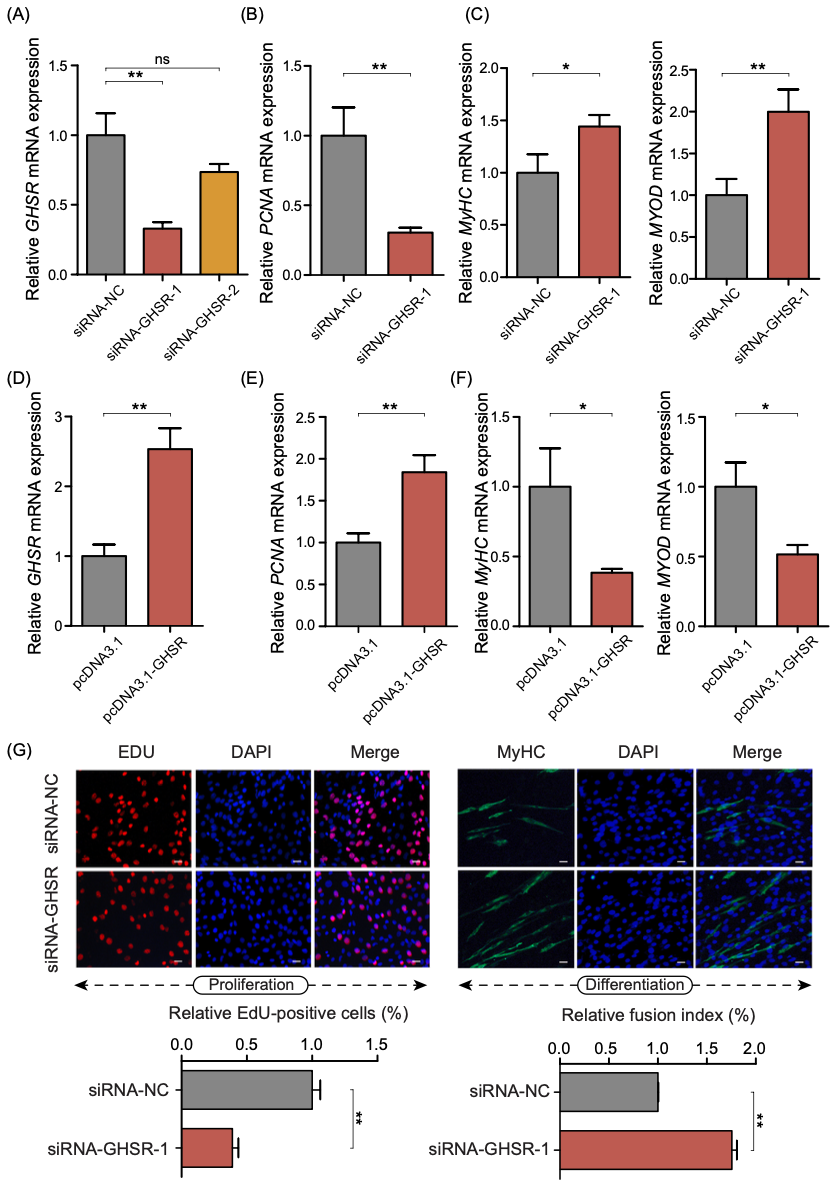


**Figure S25 Enhanced capacity for cell proliferation of *GHSR* gene.** (A) Knockdown efficiency of *GHSR* in C2C12 cells. (B) Cell proliferation assessment for the expression level of *PCNA* marker upon *GHSR* knockdown by qRT-PCR analysis in C2C12 cells. (C) Cell differentiation assessment for the expression levels of *MyHC* and *MYOD* marker upon *GHSR* knockdown by qRT-PCR analysis in C2C12 cells. (D) Overexpression efficiency of *GHSR* in C2C12 cells. (E) Cell proliferation assessment for the expression level of *PCNA* marker upon *GHSR* overexpression by qRT-PCR analysis in C2C12 cells. (F) Cell differentiation assessment for the expression levels of *MyHC* and *MYOD* marker upon *GHSR* overexpression by qRT-PCR analysis in C2C12 cells. (G) Cell proliferation and differentiation experiments measured by EDU and MyHC immunofluorescence upon *GHSR* knockdown. ^*^*p* < 0.05; ^**^*p* < 0.01; ^***^*p* < 0.001; ns, not significant.

**
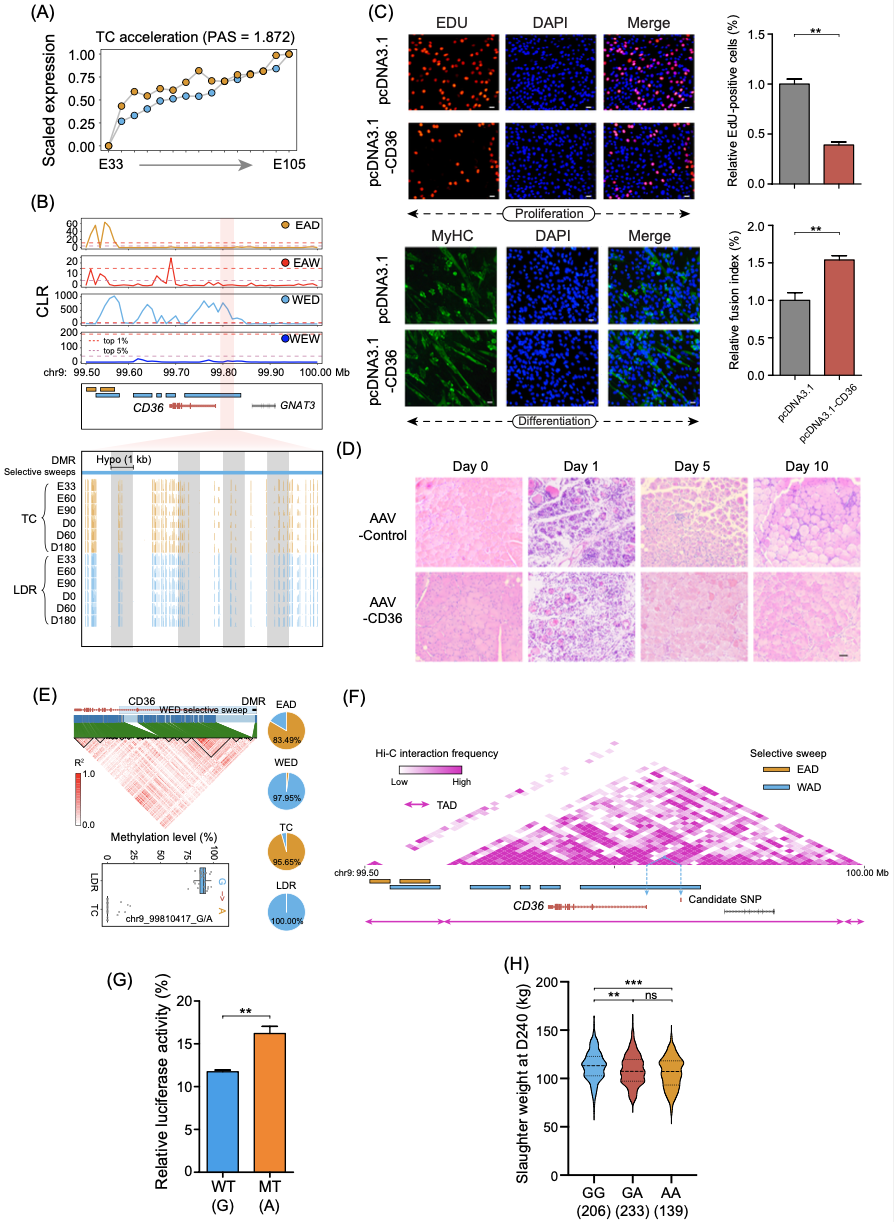
**

**Figure S26 Comprehensive analysis of the *CD36* gene associated with skeletal muscle development and meat performance.** (A) More advanced progression of *CD36* at the TC prenatal stage. (B) Visualization of methylation tracks around the swept *CD36* region. Dashed purple and red lines represent thresholds of the top 5% and 1% CLR values in the *CD36* region, respectively. (C) Cell proliferation and differentiation assays measured by EDU and MyHC immunofluorescence upon CD36 overexpression. (D) H&E staining for regenerating muscle on days 0, 1, 5 and 10 after cardiotoxin (CTX) injury in AAV-mediated non-target control and pcDNA3.1-CD36 groups, respectively. Representative images are shown at 20$\times$ magnification (scale bars = 100 μm). (E) Distinct methylation patterns and genotype spectrum between two alleles in the CpG-SNP site showing strong LD with the *CD36* gene. (F) The candidate SNP and the CD36 gene located in the same topologically associated domain (TAD) defined by Hi-C data. (G) Enhancer activities of two alleles by the luciferase reporter assay in the HEK293T cells. (H) Significant phenotypic difference in slaughter weight among three genotypes. ^*^*p* < 0.05; ^**^*p* < 0.01; ^***^*p* < 0.001; ns, not significant.


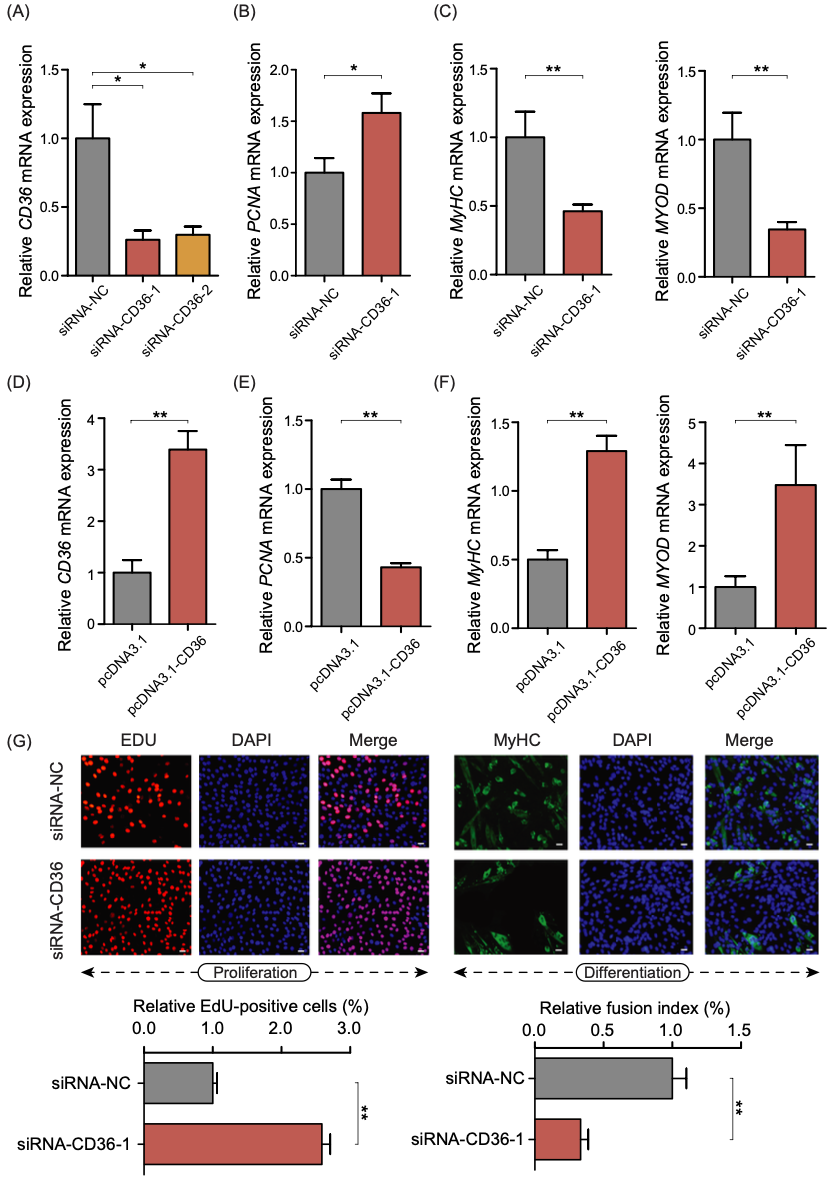


**Figure S27 Enhanced capacity for cell differentiation of *CD36* gene.** (A) Knockdown efficiency of *CD36* in C2C12 cells. (B) Cell proliferation assessment for the expression level of *PCNA* marker upon *CD36* knockdown by qRT-PCR analysis in C2C12 cells. (C) Cell differentiation assessment for the expression levels of *MyHC* and *MYOD* marker upon *CD36* knockdown by qRT-PCR analysis in C2C12 cells. (D) Overexpression efficiency of *CD36* in C2C12 cells. ^***^*p* < 0.001. (E) Cell proliferation assessment for the expression level of *PCNA* marker upon *CD36* overexpression by qRT-PCR analysis in C2C12 cells. (F) Cell differentiation assessment for the expression levels of *MyHC* and *MYOD* marker upon *CD36* overexpression by qRT-PCR analysis in C2C12 cells. (G) Cell proliferation and differentiation experiments measured by EDU and MyHC immunofluorescence upon *CD36* knockdown. ^*^*p* < 0.05; ^**^*p* < 0.01; ^***^*p* < 0.001; ns, not significant.


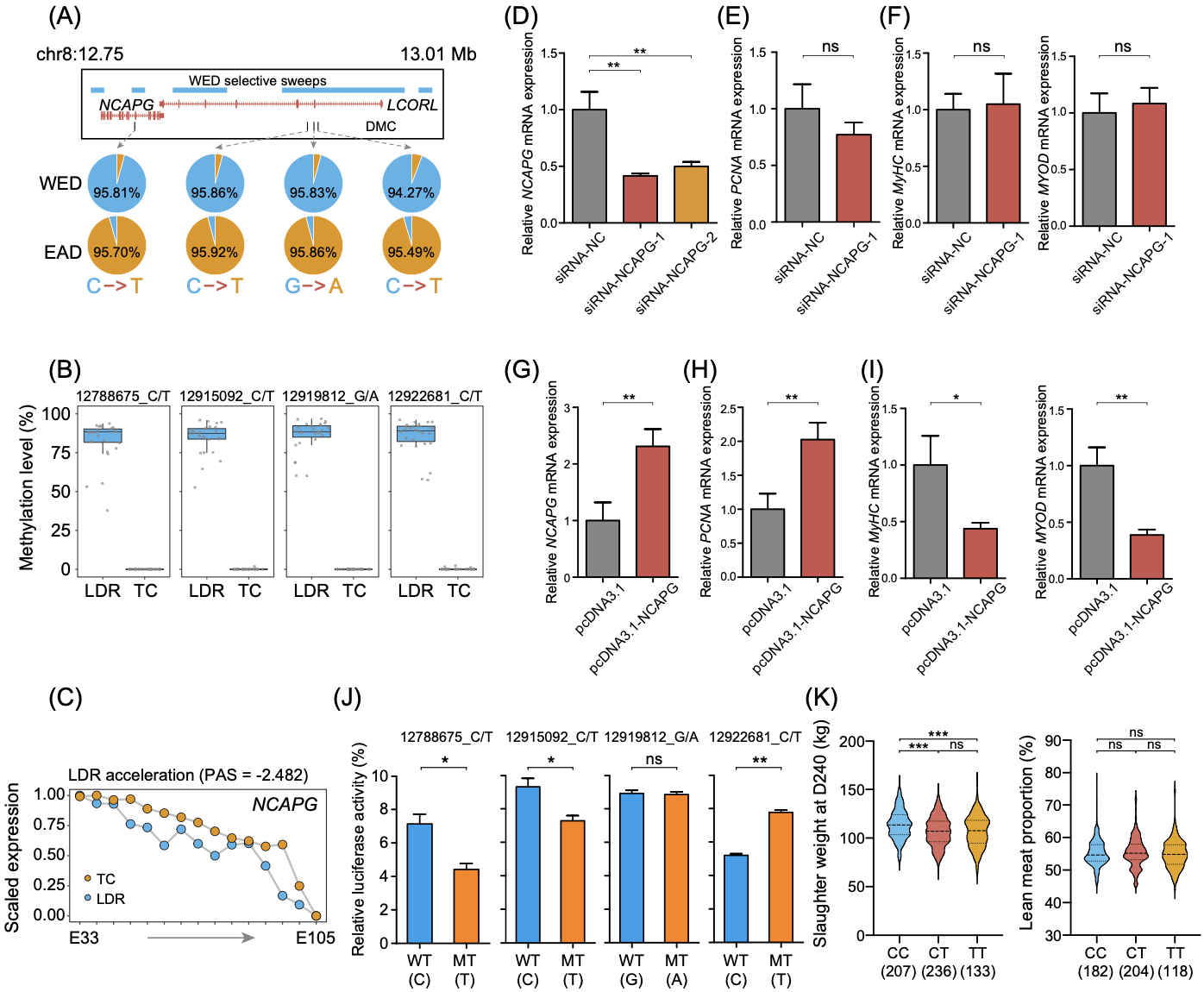


**Figure S28 Comprehensive functional analysis of *NCAPG*-*LCORL* locus.** (A) Four fixed SNPs from methylated CpG to TpG/CpA in the *NCAPG*-*LCORL* locus*.* (B) Methylation levels of the four CpG-SNPs in LDR and TC pigs. (C) Temporal advanced expression pattern of *NCAPG* gene in the LDR breed. (D) Knockdown efficiency of *NCAPG* in C2C12 cells. (E) Cell proliferation assessment for the expression level of *PCNA* marker upon *NCAPG* knockdown by qRT-PCR analysis in C2C12 cells. (F) Cell differentiation assessment for the expression levels of *MyHC* and *MYOD* marker upon *NCAPG* knockdown by qRT-PCR analysis in C2C12 cells. (G) Overexpression efficiency of *NCAPG* in C2C12 cells. (H) Cell proliferation assessment for the expression level of *PCNA* marker upon *NCAPG* overexpression by qRT-PCR analysis in C2C12 cells. (I) Cell differentiation assessment for the expression levels of *MyHC* and *MYOD* marker upon *NCAPG* overexpression by qRT-PCR analysis in C2C12 cells. (J) Comparisons of the effects of the four differential DNA variants on enhancer activity by luciferase reporter assays in HEK293T cells. (K) Phenotypic consequences of the intronic SNP in the *NCAPG* in the slaughter weight at day 240 and lean meat proportion. ^*^*p* < 0.05; ^**^*p* < 0.01; ^***^*p* < 0.001; ns, not significant.
